# Correction: Grey-box modeling and hypothesis testing of functional near-infrared spectroscopy-based cerebrovascular reactivity to anodal high-definition tDCS in healthy humans

**DOI:** 10.1371/journal.pcbi.1009734

**Published:** 2022-02-10

**Authors:** Yashika Arora, Pushpinder Walia, Mitsuhiro Hayashibe, Makii Muthalib, Shubhajit Roy Chowdhury, Stephane Perrey, Anirban Dutta

There is an error in the affiliation for authors Stephane Perrey and Makii Muthalib. The correct affiliation is: EuroMov Digital Health in Motion, Univ Montpellier, IMT Mines Ales, Montpellier, France.
