# Supplementary figures and images for: Grey-box modeling and hypothesis testing of functional near-infrared spectroscopy-based cerebrovascular reactivity to anodal high-definition tDCS in healthy humans
Source: PLoS Comput Biol. 2021 Oct 6;17(10):e1009386. doi: 10.1371/journal.pcbi.1009386 (PMC8494321; doi:10.1371/journal.pcbi.1009386)

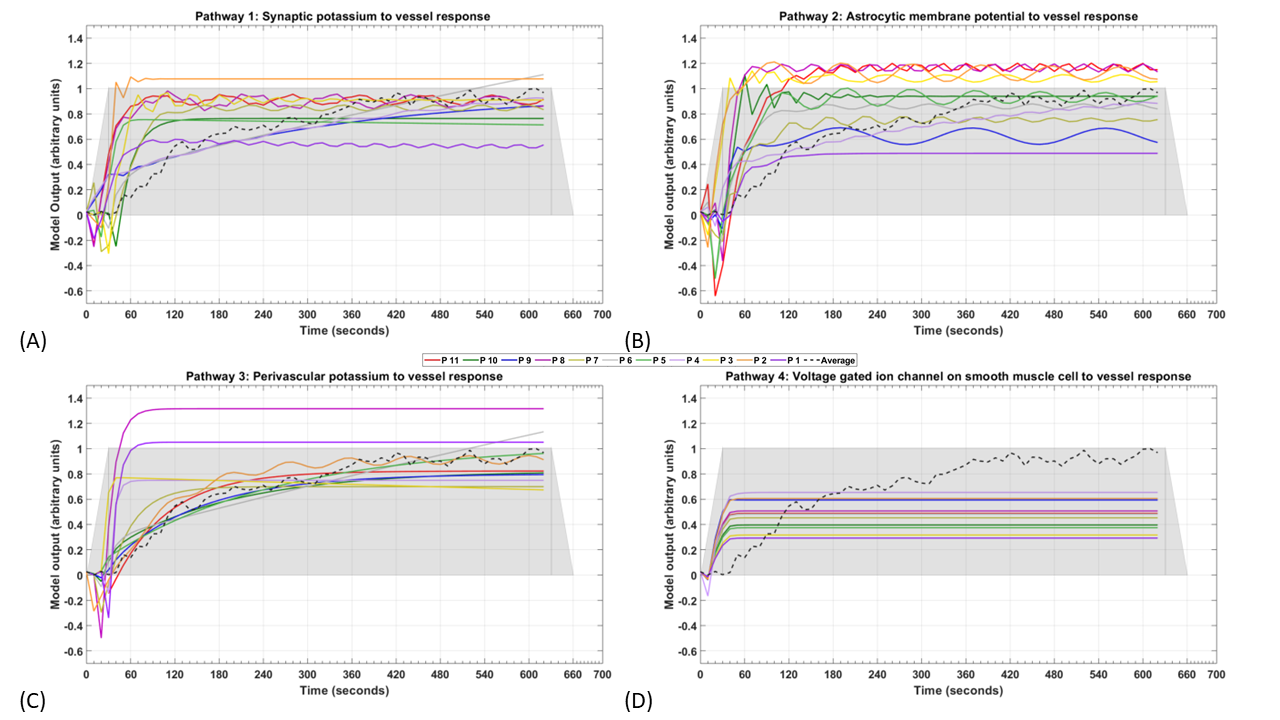

Supplement: S1 Fig — Averaged experimental fNIRS-tHb response across all subjects is also shown with a dashed line. (A) Pathway: 1: tDCS modulating vessel response through synaptic potassium pathway, (B) Pathway 2: tDCS modulating vessel response through astrocytic pathway, (C) Pathway 3: tDCS modulating vessel response through perivascular potassium pathway, (D) Pathway 4: tDCS modulating vessel response via the smooth muscle cell pathway. (TIF) [file pcbi.1009386.s001.tif]

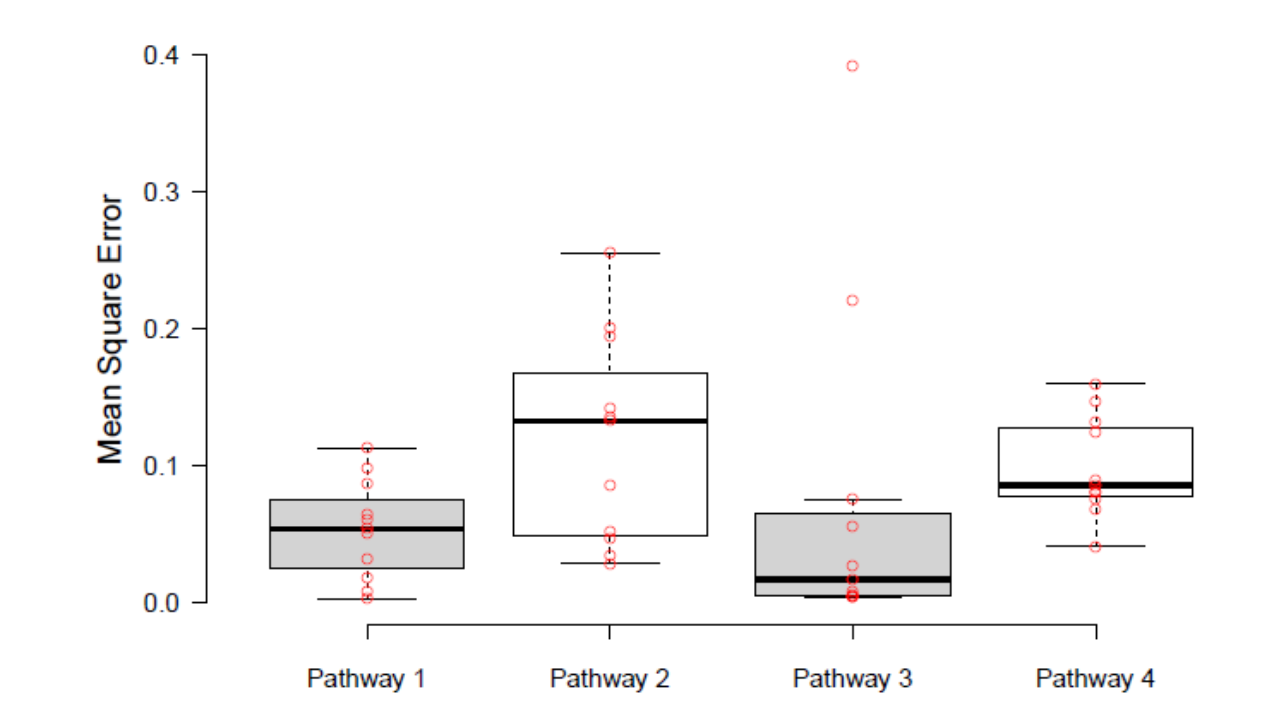

Supplement: S2 Fig — (TIF) [file pcbi.1009386.s002.tif]

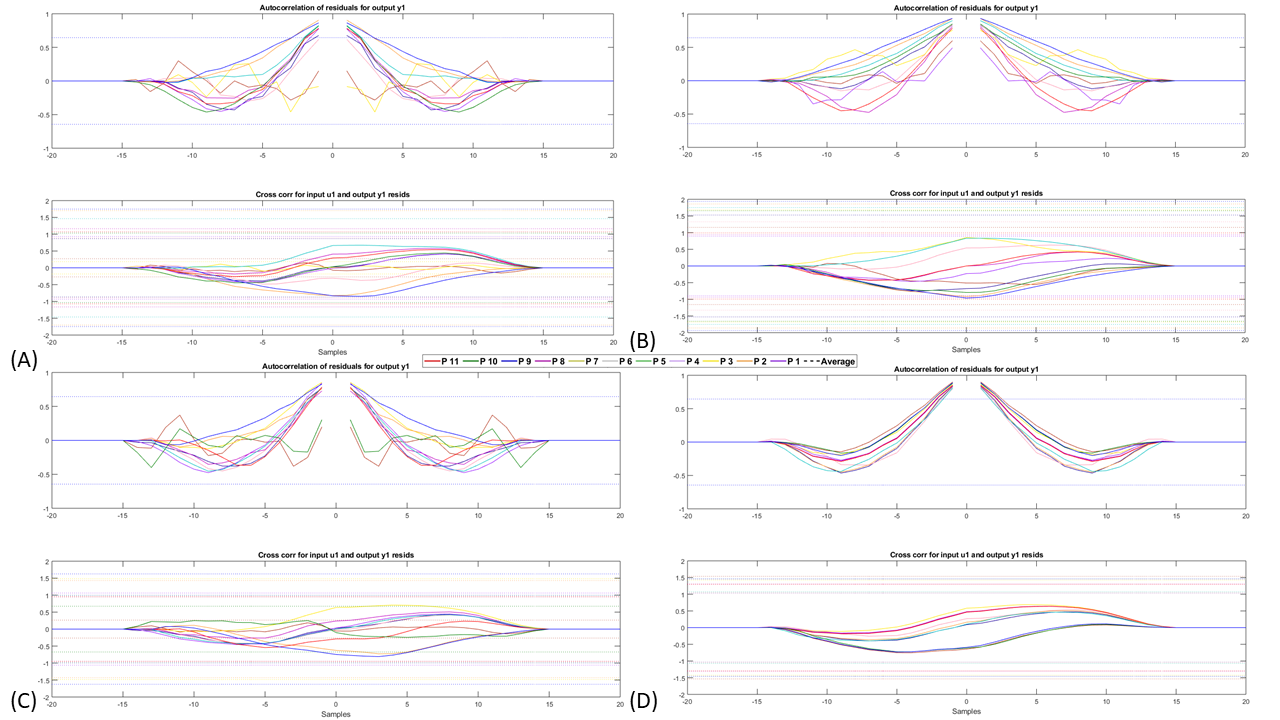

Supplement: S3 Fig — Plots show the residual analysis of the refined models obtained for the proposed pathways using grey-box model estimation data of 11 volunteers (fitted to initial 150 seconds of tDCS, model outputs presented in Fig 4 of the main manuscript). The plots display the autocorrelation curves for the residuals and cross-correlation curves between input and residuals for the proposed pathways. The confidence interval for the curves are shown by dashed lines. (A) Pathway 1: tDCS modulating vessel response through synaptic potassium pathway. (B) Pathway 2: tDCS modulating vessel response through astrocytic pathway. (C) Pathway 3: tDCS modulating vessel response through perivascular potassium pathway. (D) Pathway 4: tDCS modulating vessel response via the smooth muscle cell pathway. (TIF) [file pcbi.1009386.s003.tif]

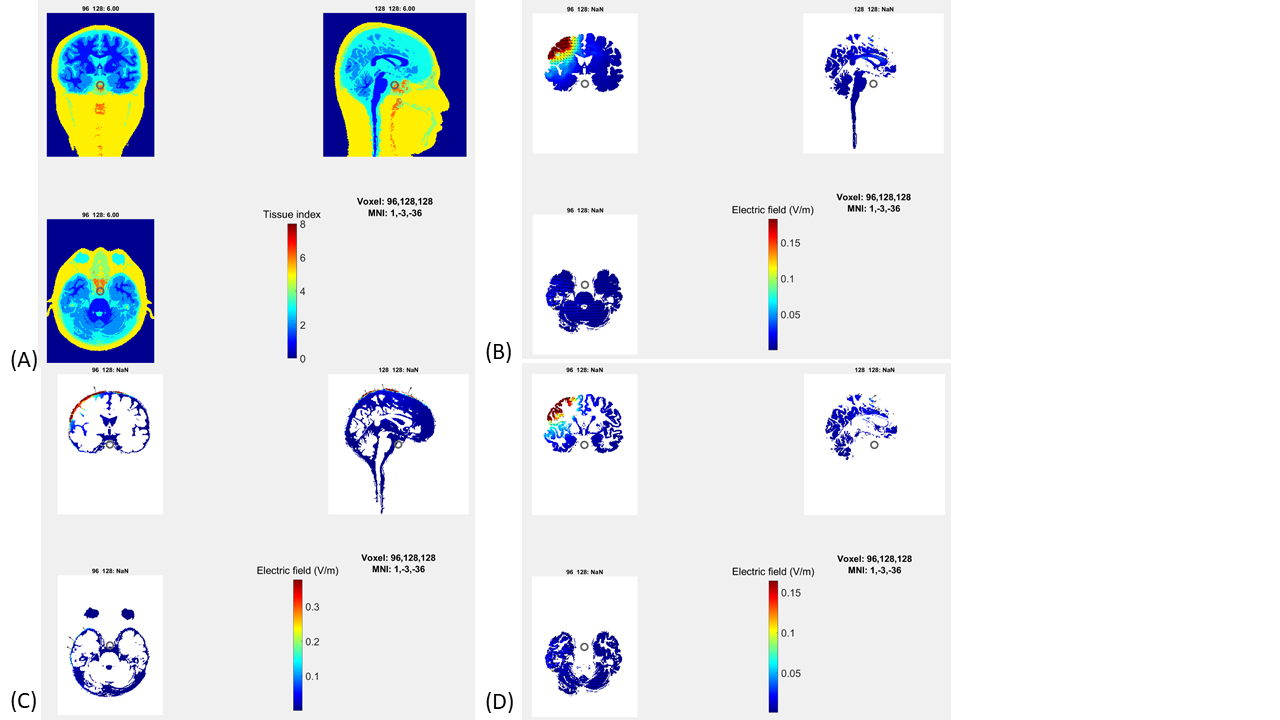

Supplement: S4 Fig — (A) Tissue segmentation for finite element modeling of the electric field using ROAST: An Open-Source, Fully-Automated, Realistic Volumetric-Approach-Based Simulator For TES. (B) Electric field (V/m) in the brain. (C) Electric field (V/m) in the cerebrospinal fluid (CSF)–note that the magnitude difference with the brain in the color scale. (D) Electric field (V/m) in the grey matter. (TIF) [file pcbi.1009386.s004.tif]

*
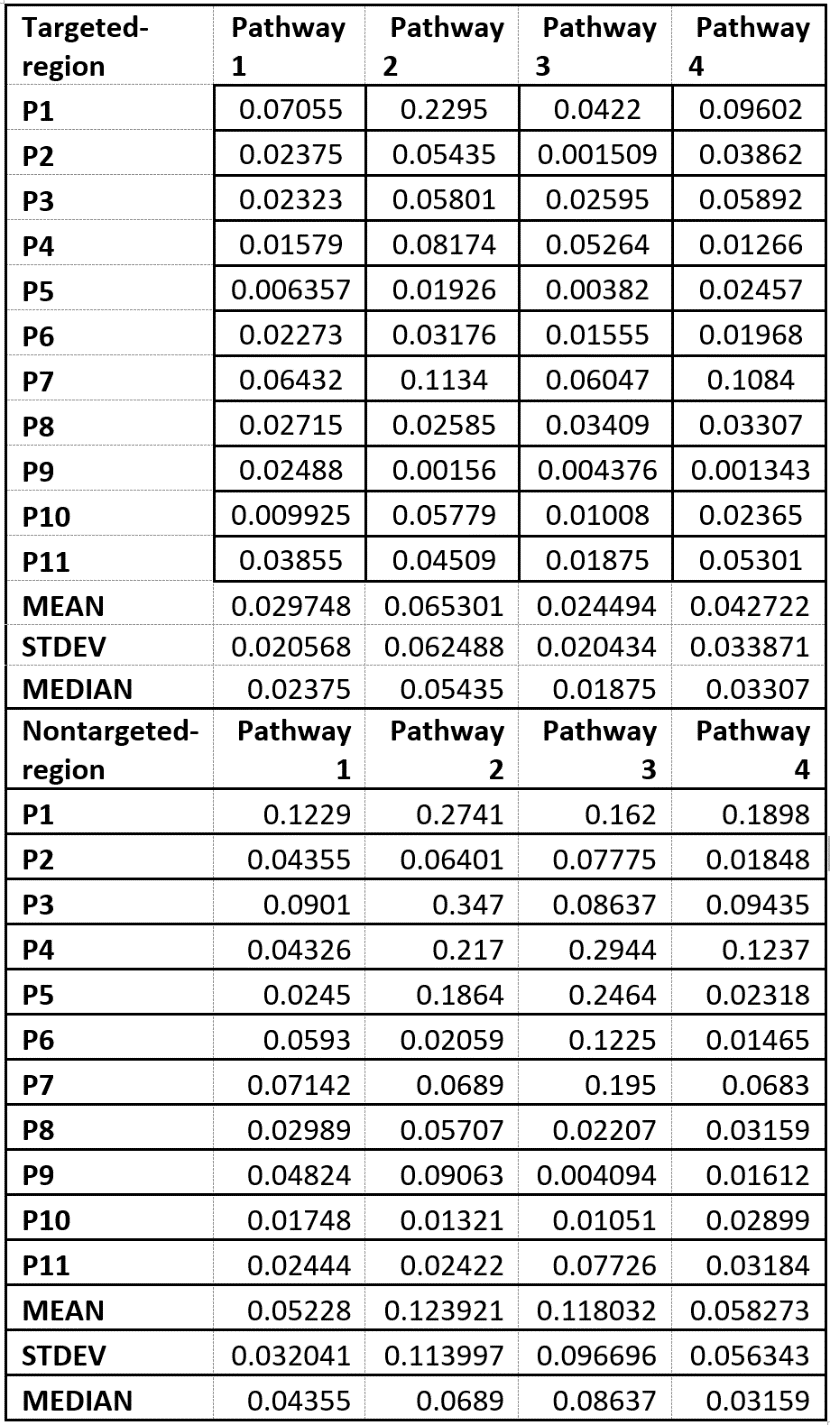
*

Supplement: S5 Table — Subjects P3, P4, P10 have >-0.5 Correlation Coefficient between Oxy-Hb & Dxy-Hb. (DOCX) [file pcbi.1009386.s011.docx]

*
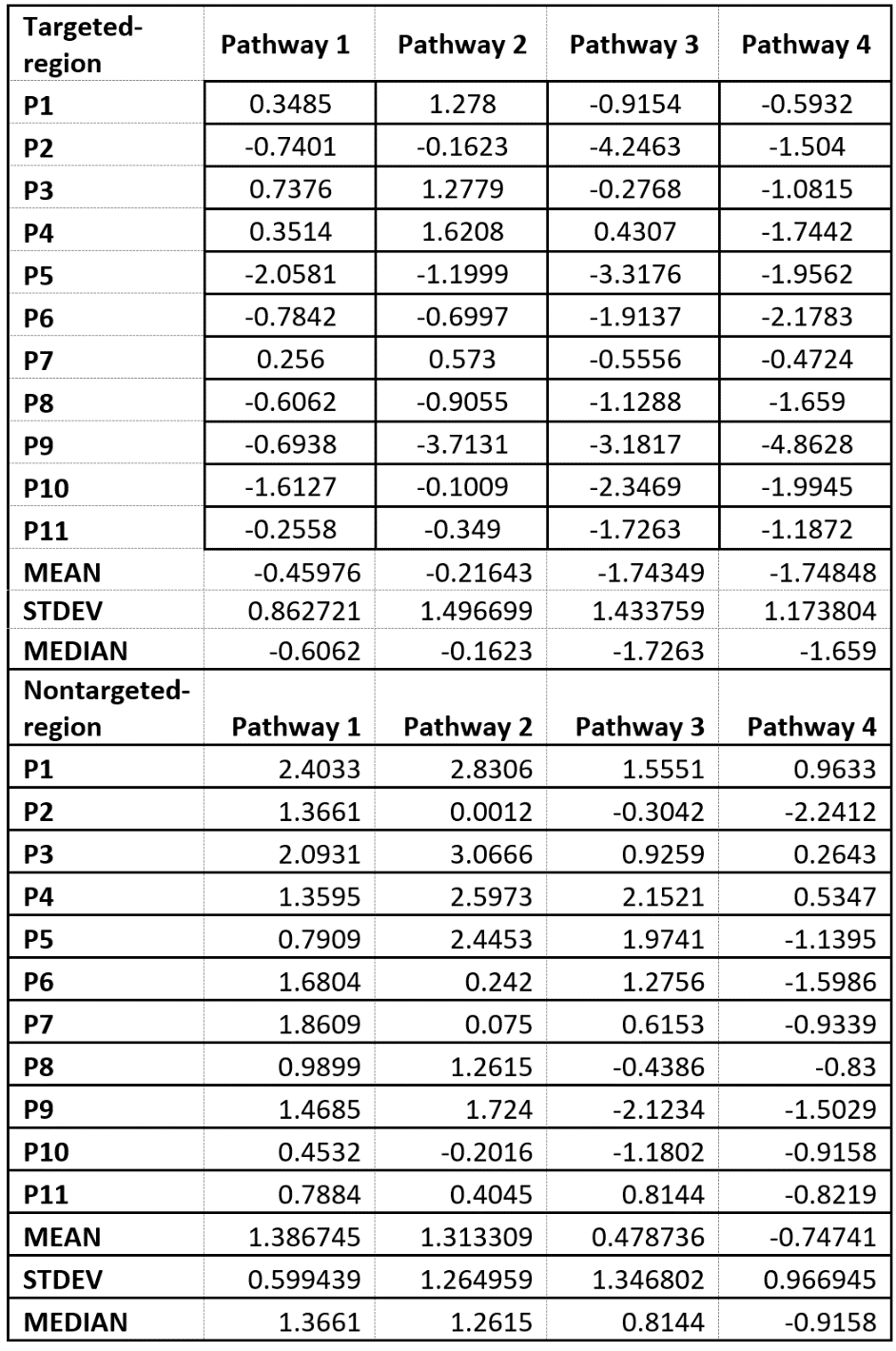
*

Supplement: S6 Table — Subjects P3, P4, P10 have >-0.5 Correlation Coefficient between Oxy-Hb & Dxy-Hb. (DOCX) [file pcbi.1009386.s012.docx]
