# Supplementary material for: Grey-box modeling and hypothesis testing of functional near-infrared spectroscopy-based cerebrovascular reactivity to anodal high-definition tDCS in healthy humans
Source: PLoS Comput Biol. 2021 Oct 6;17(10):e1009386. doi: 10.1371/journal.pcbi.1009386 (PMC8494321; doi:10.1371/journal.pcbi.1009386)
Supplement: S1 Text — (DOCX) [file pcbi.1009386.s005.docx]

**S1 Text: Model Equations for Neurovascular Compartmental Dynamics**

The following equations are obtained from published literature models’ implementation of neurovascular dynamics. Equations in bold font represent state variables of the compartments.

**1. Synaptic Space**

*(a) Potassium concentration in the synaptic space,* $\left[ K^{+} \right]_{s}$

$\frac{\boldsymbol{d}{\boldsymbol{[}\boldsymbol{K}^{\boldsymbol{+}}\boldsymbol{]}}_{\boldsymbol{s}}}{\boldsymbol{dt}}\boldsymbol{=}\boldsymbol{J}_{\boldsymbol{K}_{\boldsymbol{s}}}\boldsymbol{-}\boldsymbol{J}_{\boldsymbol{\Sigma Kmax}}\boldsymbol{k}_{\boldsymbol{Na}}\frac{{\boldsymbol{[}\boldsymbol{K}^{\boldsymbol{+}}\boldsymbol{]}}_{\boldsymbol{s}}}{{\boldsymbol{[}\boldsymbol{K}^{\boldsymbol{+}}\boldsymbol{]}}_{\boldsymbol{s}}\boldsymbol{+}\boldsymbol{KKO}_{\boldsymbol{a}}}$ **(1)**

$J_{K_{s}}$ is potassium release from active neurons. $J_{\Sigma Kmax}$ is maximum flux, $k_{Na}$ is a constant parameter that depends on extracellular sodium concentration, ${KKO}_{a}$is the threshold value for ${[K^{+}]}_{s}$.

**2. Astrocytic Intracellular Space**

*(a) Astrocytic Inositol trisphosphate,* $\left[ {IP}_{3} \right]$

$\frac{\boldsymbol{d}\left[ \boldsymbol{IP}_{\boldsymbol{3}} \right]}{\boldsymbol{dt}}\boldsymbol{=}\boldsymbol{r}_{\boldsymbol{h}}^{\boldsymbol{*}}\frac{\boldsymbol{\rho+\delta}}{\boldsymbol{K}_{\boldsymbol{G}}\boldsymbol{+\rho+\delta}}\boldsymbol{-}\boldsymbol{k}_{\boldsymbol{deg}}\left[ \boldsymbol{IP}_{\boldsymbol{3}} \right]$ **(2)**

$r_{h}^{*}$ is ${IP}_{3}$ production rate and $\rho$ is ratio of bound to unbound receptors and is given as

$\rho= \frac{[Glu]}{(K_{Glu}+\left[ Glu \right])}$ (3)

$[Glu]$ is input synaptic glutamate release, $K_{Glu}$ is Dissociation Constant, $k_{deg}$ is Degradation rate and $\delta$ is the ratio of the activities of bound and unbound receptors.

*(b) Astrocytic intracellular calcium concentration,*$\left[ {Ca}^{2+} \right]_{A}$

$\frac{\boldsymbol{d}\left[ {Ca}^{2+} \right]_{A}}{\boldsymbol{dt}}\boldsymbol{= \beta}\left( \boldsymbol{J}_{\boldsymbol{IP}_{\boldsymbol{3}}}\boldsymbol{-}\boldsymbol{J}_{\boldsymbol{pump}}\boldsymbol{+}\boldsymbol{J}_{\boldsymbol{leak}}\boldsymbol{+}\boldsymbol{J}_{\boldsymbol{TRPV}} \right)$ **(4)**

$\beta$ is ${Ca}^{2+}$buffering factor, $J_{{IP}_{3}}$is rate of $\left[ {Ca}^{2+} \right]_{A}$concentration change due to release through IP3 binded receptors (${IP}_{3}R$) on the endoplasmic reticulum (ER) channels, $J_{pump}$ is rate of $\left[ {Ca}^{2+} \right]_{A}$concentration change due to pump uptake into the endoplasmic reticulum (ER) and $J_{TRPV}$ is rate of $\left[ {Ca}^{2+} \right]_{A}$ concentration change due to extracellular space through transient receptor potential vanniloid-related 4 (TRPV4) channels

$J_{{IP}_{3}}= J_{max}\left[ \left( \frac{\left[ {IP}_{3} \right]}{\left[ {IP}_{3} \right]+K_{I}} \right)\left( \frac{\left[ {Ca}^{2+} \right]_{A}}{\left[ {Ca}^{2+} \right]_{A}+K_{act}} \right)h \right]^{3}$ (5)

$J_{max}$ is the maximum rate, $K_{I}$ is the dissociation constant for IP3 binding to an IP3R, $K_{act}$ is the dissociation constant for ${Ca}^{2+}$ binding to an activation site on an IP3R, $\left[ {Ca}^{2+} \right]_{ER}$ is the ${Ca}^{2+}$ concentration in the ER.

$J_{pump}= V_{max}\frac{{\left[ {Ca}^{2+} \right]_{A}}^{2}}{{\left[ {Ca}^{2+} \right]_{A}}^{2}+ K_{p}^{2}}$ (6)

$V_{max}$ is the maximum pump rate and K_p_ is the pump constant.

$J_{leak}= P_{L}\left( 1-\frac{\left[ {Ca}^{2+} \right]_{A}}{\left[ {Ca}^{2+} \right]_{ER}} \right)$ (7)

where $P_{L}$ is determined by the steady-state flux balance.

$J_{TRPV}= {I_{TRPV}}/{C_{astr}\gamma}$ (8)

$I_{TRPV}$is electrical current through the TRPV channel, $C_{astr}$ is the astrocyte cell capacitance, and $\gamma$ is a scaling factor for relating the net movement of ion fluxes to the membrane potential.

$I_{TRPV}= g_{TRPV}s\left( V_{k}-v_{TRPV} \right)$ (9)

$g_{TRPV}$is maximum channel conductance, $V_{k}$is membrane potential, $v_{TRPV}$ is TRPV channel reversal potential, s is TRPV4 channel open probability.

*(c) Gating variable h*

$\frac{\boldsymbol{dh}}{\boldsymbol{dt}}\boldsymbol{=}\boldsymbol{k}_{\boldsymbol{on}}\left[ \boldsymbol{K}_{\boldsymbol{inh}}\boldsymbol{-}\left( \left[ {Ca}^{2+} \right]_{A}\boldsymbol{+}\boldsymbol{K}_{\boldsymbol{inh}} \right)\boldsymbol{h} \right]$ **(10)**

$k_{on}$ is $\left[ {Ca}^{2+} \right]_{A}$binding rate at the inhibitory site on the IP3R and $K_{inh}$ is dissociation constant at the inhibitory site on the IP3R.

*(d) The TRPV4 channel open probability, s*

$\frac{\boldsymbol{ds}}{\boldsymbol{dt}}\boldsymbol{=}\frac{\boldsymbol{1}}{\boldsymbol{\tau}_{\boldsymbol{(}\left[ \boldsymbol{Ca}^{\boldsymbol{2+}} \right]_{\boldsymbol{P}}\boldsymbol{)}}}\left( \boldsymbol{s}_{\boldsymbol{\infty}}\boldsymbol{- s} \right)$ **(11)**

$\tau_{(\left[ {Ca}^{2+} \right]_{P})}$ is time constant and is a function of $\left[ {Ca}^{2+} \right]_{P}$

$\tau_{\left( \left[ {Ca}^{2+} \right]_{P} \right)}= {\tau_{TRPV}}/{\left[ {Ca}^{2+} \right]_{P}}$ (12)

$\left[ {Ca}^{2+} \right]_{P}$ is perivascular $\left[ {Ca}^{2+} \right]$ concentration expressed in mM, $s_{\infty}$is steady-state channel open probability and is a function of strain and $\left[ {Ca}^{2+} \right]$

$s_{\infty}= \left( \frac{1}{1+ e^{-(\varepsilon-\varepsilon_{1/2})/\kappa}} \right)\left[ \frac{1}{1+H_{Ca}}\left( H_{Ca}+\tanh\left( \frac{V_{k}- v_{1,TRPV}}{v_{2,TRPV}} \right) \right) \right]$ (13)

where, $1/{(1+ e^{-(\varepsilon-\varepsilon_{1/2})/\kappa})}$is material strain gating, ε is strain on the perivascular endfoot and is given as

$\varepsilon= {(x- x_{0})}/x$ (14)

x is vessel circumferential contraction and dilation variable, $\varepsilon_{1/2}$is strain required for half activation, $H_{Ca}$ is inhibitory factor and is given as

$H_{Ca}= \left( \frac{\left[ {Ca}^{2+} \right]_{A}}{\gamma_{{Ca}_{i}}}+ \frac{\left[ {Ca}^{2+} \right]_{P}}{\gamma_{{Ca}_{e}}} \right)$ (15)

where $\gamma_{{Ca}_{i}}$ and $\gamma_{{Ca}_{e}}$ are constants associated with intra and extra- cellular $\left[ {Ca}^{2+} \right]$, respectively.

*(e) Calcium dependent EET (Epoxyeicosatrienoic Acid) production in the cell,* $\left[ EET \right]$

$\frac{\boldsymbol{d[EET]}}{\boldsymbol{dt}}\boldsymbol{=}\boldsymbol{V}_{\boldsymbol{EET}}\left( \left[ {Ca}^{2+} \right]_{A}\boldsymbol{-}\left[ \boldsymbol{Ca}^{\boldsymbol{2+}} \right]_{\boldsymbol{min}} \right)\boldsymbol{-}\boldsymbol{k}_{\boldsymbol{EET}}\left[ \boldsymbol{EET} \right]$ **(16)**

$V_{EET}$ is EET production rate, $\left[ {Ca}^{2+} \right]_{min}$ is minimum $\left[ {Ca}^{2+} \right]$ required for EET production and $k_{EET}$ is EET decay rate.

*(f) Open BK (Big Potassium) channel probability,* $n_{BK}$

$\frac{\boldsymbol{d}\boldsymbol{n}_{\boldsymbol{BK}}}{\boldsymbol{dt}}\boldsymbol{=}\boldsymbol{\phi}_{\boldsymbol{BK}}\left( \boldsymbol{n}_{\boldsymbol{\infty}}\left[ \boldsymbol{BK} \right]\boldsymbol{-}\boldsymbol{n}_{\boldsymbol{BK}} \right)$ **(17)**

$\phi_{BK}$is time constant associated with the opening of astrocyte BK channels and is based on statistical considerations

$\phi_{BK}= \Psi_{BK}\cosh\left( \frac{V_{k}-v_{3,BK}}{2v_{4,BK}} \right)$ (18)

$n_{\infty}\left[ BK \right]=0.5 \left( 1+\tanh\left( \frac{V_{k}+{EET}_{shift}\left[ EET \right]- v_{3,BK}}{v_{4,BK}} \right) \right)$ (19)

$v_{3,BK}= -\frac{v_{5,BK}}{2}\tanh\left( \frac{\left[ {Ca}^{2+} \right]_{A}- {Ca}_{3,BK}}{{Ca}_{4,BK}} \right)+ v_{6,BK}$ (20)

$\Psi_{BK} is$characteristic time, $v_{3,BK}$is the voltage associated with the opening of half the population, $V_{k}$ is astrocyte membrane potential, $v_{4,BK}$is measure of the spread of the distribution, $v_{5,BK}$determines the range of the shift of $n_{\infty}\left[ BK \right]$ as calcium varies.

${EET}_{shift}$ determines the EET-dependent shift of the channel reversal potential, ${Ca}_{3,BK}$and ${Ca}_{4,BK}$ constants associated with calcium concentration/ calcium dependent constants

*(g) Astrocyte membrane potential,* $V_{k}$

$\frac{\boldsymbol{d}\boldsymbol{V}_{\boldsymbol{k}}}{\boldsymbol{dt}}\boldsymbol{=}\frac{\boldsymbol{1}}{\boldsymbol{C}_{\boldsymbol{astr}}}\left( \boldsymbol{-I}_{\boldsymbol{BK}}\boldsymbol{-}\boldsymbol{I}_{\boldsymbol{leak}}\boldsymbol{-}\boldsymbol{I}_{\boldsymbol{TRPV}}\boldsymbol{-}\boldsymbol{I}_{\boldsymbol{\Sigma K}} \right)$ **(21)**

$I_{BK}$ is current through BK channel

$I_{BK}= g_{BK}n_{BK}\left( V_{k}-v_{BK} \right)$ (22)

$J_{BK}= I_{BK}/(C_{astr}\gamma)$ (23)

$g_{BK}$is channel conductance, $v_{BK}$is reversal potential, $I_{\Sigma K}$ is the electrical current carried by the K^+^ influx at the perisynaptic process and $I_{leak}$is leak current

$I_{\Sigma K}= - J_{\Sigma K}C_{astr}\gamma$(24)

$I_{leak}= g_{leak}\left( V_{k}-v_{leak} \right)$ (25)

$g_{leak}$ is the leak conductance and $v_{leak}$is reversal potential.

**3. Perivascular Space**

*(a) Perivascular potassium concentration,* $\left[ \boldsymbol{K}^{\boldsymbol{+}} \right]_{\boldsymbol{P}}$

$\frac{\boldsymbol{d}\left[ \boldsymbol{K}^{\boldsymbol{+}} \right]_{\boldsymbol{P}}}{\boldsymbol{dt}}\boldsymbol{=}\frac{\boldsymbol{J}_{\boldsymbol{BK}}}{\boldsymbol{VR}_{\boldsymbol{pa}}}\boldsymbol{+}\frac{\boldsymbol{J}_{\boldsymbol{KIR}}}{\boldsymbol{VR}_{\boldsymbol{ps}}}\boldsymbol{-}\boldsymbol{R}_{\boldsymbol{decay}}\left( \left[ \boldsymbol{K}^{\boldsymbol{+}} \right]_{\boldsymbol{P}}\boldsymbol{-}\left[ \boldsymbol{K}^{\boldsymbol{+}} \right]_{\boldsymbol{P,min}} \right)$ **(26)**

${[K^{+}]}_{P,min}$ is the resting state equilibrium K^+^ concentration in the perivascular space. The potassium flow from the astrocyte and SMC are $J_{BK}$ and $J_{KIR}$corresponding to big potassium (BK) and inward rectifying potassium (KIR) respectively. And, ${VR}_{pa}$ and ${VR}_{ps}$ are the volume ratios of perivascular space to astrocyte and SMC, respectively. $R_{decay}$ is the rate at which perivascular K^+^ concentration decays to its baseline state.

$J_{BK}= I_{BK}/(C_{astr}\gamma)$(27)

$J_{KIR}= I_{KIR}/(C_{SMC}\gamma)$(28)

$C_{SMC}$ is the SMC (smooth muscle cell) capacitance.

$I_{KIR}= g_{KIR}k\left( V_{m}-v_{KIR} \right)$ (29)

Here $g_{KIR}$is channel conductance, $v_{KIR}$is reversal potential and k is open probability. These all depend on the perivascular K^+^ concentration

$g_{KIR}= g_{KIR,0}\sqrt{{[K^{+}]}_{P}}$ (30)

$g_{KIR,0}$ is the conductance when the perivascular K^+^ concentration is 1mM.

$v_{KIR}= v_{KIR,1}log{[K^{+}]}_{P}- v_{KIR,2}$ (31)

$v_{KIR,1}$ and $v_{KIR,2}$ are constants

*(b) Perivascular Ca^2+^ concentration,* $\left[ {Ca}^{2+} \right]_{P}$

$\frac{\boldsymbol{d}\left[ \boldsymbol{Ca}^{\boldsymbol{2+}} \right]_{\boldsymbol{P}}}{\boldsymbol{dt}}\boldsymbol{= -}\frac{\boldsymbol{J}_{\boldsymbol{TRPV}}}{\boldsymbol{VR}_{\boldsymbol{pa}}}\boldsymbol{-}\frac{\boldsymbol{J}_{\boldsymbol{Ca}}}{\boldsymbol{VR}_{\boldsymbol{ps}}}\boldsymbol{-}\boldsymbol{Ca}_{\boldsymbol{decay}}\left( \left[ \boldsymbol{Ca}^{\boldsymbol{2+}} \right]_{\boldsymbol{P}}\boldsymbol{-}\left[ \boldsymbol{Ca}^{\boldsymbol{2+}} \right]_{\boldsymbol{P,min}} \right)$ **(32)**

$J_{Ca}$ is calcium current from the arteriole SMC, ${Ca}_{decay}$is the decay rate of perivascular Ca^2+^ concentration

$J_{Ca}={{-I}_{Ca}}/{C_{SMC}\gamma}$ (33)

$I_{Ca}= g_{Ca}m_{\infty}\left( V_{m}-v_{ca} \right)$ (34)

$g_{Ca}$is channel conductance, $v_{Ca}$is reversal potential

$m_{\infty}=0.5 \left( 1+tanh\frac{V_{m}- v_{1}}{v_{2}} \right)$ (35)

$v_{1}$ and $v_{2}$are constants.

**4. Arteriole smooth muscle cell (SMC) intracellular space**

*(a) Open KIR (Inward Rectifying Potassium) channel probability, k*

$\frac{\boldsymbol{dk}}{\boldsymbol{dt}}\boldsymbol{=}\frac{\boldsymbol{1}}{\boldsymbol{\tau}_{\boldsymbol{k}}}\left( \boldsymbol{k}_{\boldsymbol{\infty}}\boldsymbol{-k} \right)$ **(36)**

$\tau_{k}=1/(\alpha_{k}+ \beta_{k})$ (37)

$k_{\infty}= \alpha_{k}/(\alpha_{k}+ \beta_{k})$ (38)

$\alpha_{k}= \alpha_{KIR}/\left( 1+\exp\left( \frac{V_{m}-v_{KIR}+a_{v_{1}}}{a_{v_{2}}} \right) \right)$ (39)

$\beta_{k}=\beta_{KIR}\exp\left( b_{v_{2}}\left( V_{m}-v_{KIR}+b_{v_{1}} \right) \right)$ (40)

$\alpha_{KIR},\beta_{KIR}, a_{v_{1}}, a_{v_{2}}, b_{v_{1}},b_{v_{2}}$are constants.

*(b) SMC membrane potential, V_SMC_*

$\frac{\boldsymbol{d}\boldsymbol{V}_{\boldsymbol{SMC}}}{\boldsymbol{dt}}\boldsymbol{=}\frac{\boldsymbol{1}}{\boldsymbol{C}_{\boldsymbol{SMC}}}\left( \boldsymbol{-}\boldsymbol{I}_{\boldsymbol{L}}\boldsymbol{-}\boldsymbol{I}_{\boldsymbol{K}}\boldsymbol{-}\boldsymbol{I}_{\boldsymbol{Ca}}\boldsymbol{-}\boldsymbol{I}_{\boldsymbol{KIR}} \right)$ **(41)**

$I_{K}= g_{K}n\left( V_{m}- v_{k} \right)$ (42)

$I_{L}= g_{L}\left( V_{m}- v_{l} \right)$ (43)

*(c) Open potassium channel probability, n*

$\frac{\boldsymbol{dn}}{\boldsymbol{dt}}\boldsymbol{=}\boldsymbol{\lambda}_{\boldsymbol{n}}\left( \boldsymbol{n}_{\boldsymbol{\infty}}\boldsymbol{- n} \right)$ **(44)**

$n_{\infty}=0.5 \left( 1+\tanh\frac{V_{m}- v_{3}}{v_{4}} \right)$ (45)

$\lambda_{n}= \phi_{n}\cosh\frac{V_{m}- v_{3}}{2v_{4}}$ (46)

$v_{3}= -\frac{v_{5}}{2}\tanh\frac{{[{Ca}^{2+}]}_{SMC}- {Ca}_{3}}{{Ca}_{4}}+ v_{6}$ (47)

$v_{3}, v_{4}, v_{5},v_{6}, {Ca}_{3}, {Ca}_{4}$ are constants.

*(d) Ca^2+^ concentration in SMC,* ${[{Ca}^{2+}]}_{SMC}$

$\frac{\boldsymbol{d}{\boldsymbol{[}\boldsymbol{Ca}^{\boldsymbol{2+}}\boldsymbol{]}}_{\boldsymbol{SMC}}}{\boldsymbol{dt}}\boldsymbol{= -}\boldsymbol{\rho}^{\boldsymbol{'}}\left( \boldsymbol{\alpha}\boldsymbol{I}_{\boldsymbol{ca}}\boldsymbol{+}\boldsymbol{k}_{\boldsymbol{ca}}\left[ \boldsymbol{Ca}^{\boldsymbol{2+}} \right]_{\boldsymbol{SMC}} \right)$ **(48)**

$\rho^{'}= \frac{{(K_{d}+\left[ {Ca}^{2+} \right]_{SMC})}^{2}}{{(K_{d}+\left[ {Ca}^{2+} \right]_{SMC})}^{2}+K_{d}B_{T}}$ (49)

$\alpha$ is the Faraday constant times cytosol volume, is the constant ratio of Ca^2+^ outflux to influx, $K_{d}$ is the rate constant in the calcium buffer reaction and $B_{T}$ is the total buffer concentration.

*(e) Fraction of attached cross bridges, ω*

For muscle mechanics, a given myosin-actin overlapping segment can have a fraction of cross bridges between myosin and actin filaments represented as ω and is given as

$\frac{\boldsymbol{d\omega}}{\boldsymbol{dt}}\boldsymbol{=}\boldsymbol{k}_{\boldsymbol{\Psi}}\left( \frac{\boldsymbol{\Psi}}{\boldsymbol{\Psi}_{\boldsymbol{m}}\boldsymbol{+\Psi}}\boldsymbol{- \omega} \right)$ **(50)**

$k_{\Psi}$ is rate constant and $\Psi_{m}$ is a constant, $\Psi$ is given as

$\Psi= \left[ {Ca}^{2+} \right]_{SMC}^{q}/({Ca}_{m}^{q}+\left[ {Ca}^{2+} \right]_{SMC}^{q})$ (51)

Ca_m_ and q and are constants.

*(f) Mean circumference of the vessel, x*

$\frac{\boldsymbol{dx}}{\boldsymbol{dt}}\boldsymbol{=}\frac{\boldsymbol{1}}{\boldsymbol{\tau}}\left( \boldsymbol{f}_{\boldsymbol{\Delta}_{\boldsymbol{p}}}\boldsymbol{-}\boldsymbol{f}_{\boldsymbol{x}}\boldsymbol{-}\boldsymbol{f}_{\boldsymbol{u}} \right)$ **(52)**

τ is time constant related with the wall internal friction and $f_{\Delta_{p}}$is force on the vessel due to transmural pressure $\Delta_{p}$ which is expressed as

$f_{\Delta_{p}}= \frac{1}{2}\Delta_{p}\left( \frac{x}{\pi}-\frac{A}{x} \right)$ (53)

Here, A is cross-sectional area of vessel given as,

$A= \pi\left( r_{o}^{2}- r_{i}^{2} \right)$ (54)

$r_{o}$is the outer radius of vessel, $r_{i}$ is the inner radius of vessel, $r_{m}$ is the mean radius of vessel $r_{m}={(r_{o}+r_{i})}/2$ and ${x=2\pi r}_{m}$.

Considering a segment of the vessel as a cylindrical element with thickness $\left( r_{o}-r_{i} \right),$ and unit length along the axis and having the longitudinal cross-sectional surface area of $S=1/(r_{o}-r_{i})$. The stresses on S are represented through a Maxwell model along the mean circumference (x) which consists of a contractile component of length y, a series elastic component of length u, a parallel elastic component of length $x=u+y$, and a parallel viscous component. The hoop forces on S are:

$f_{x}= w_{e}S\sigma_{x}^{'}\sigma_{0}^{\#}$ , and

$f_{u}= w_{m}S\sigma_{u}^{'}\sigma_{0}^{\#}$ (55)

$f_{x}$ is hoop force on S due to the visoelastic stress and $f_{u}$is hoop force on S due to the myogenic stress. $w_{e}$ is viscoelastic contributed weight, $w_{m}$ is myogenic hoop forces contributed weight, $\sigma_{x}^{'}$ is normalized hoop stresses associated with x (circumferential) component, $\sigma_{u}^{'}$ is normalized hoop stresses associated with u (series elastic component) component and $\sigma_{y}^{'}$is normalized hoop stresses associated with y (contractile component) component. For initial values, $x_{0}=2\pi r_{0}$($r_{0}$ is the initial mean radius of the vessel).

$\sigma_{x}^{'}= x_{3}^{'}\left( 1+\tanh\frac{x^{'}- x_{1}^{'}}{x_{2}^{'}} \right)+x_{4}^{'}\left( x^{'}- x_{5}^{'} \right)- x_{8}^{'}\left( \frac{x_{6}^{'}}{x^{'}-x_{7}^{'}} \right)^{2}- x_{9}^{'}$ (56) $\sigma_{u}^{'}= u_{2}^{'}\exp\left( u_{1}^{'}u^{'} \right)-u_{3}^{'}$ (57)

Normalized values are $x^{'}=x/x_{0}, y^{'}=y/x_{0},u^{'}=u/x_{0},y_{0}^{'}=y_{0}/x_{0}, \sigma_{x}^{'}= \sigma_{x}/\sigma_{0}^{\#}$, $\sigma_{y}^{'}= \sigma_{y}/\sigma_{0}^{\#}$,$\sigma_{u}^{'}= \sigma_{u}/\sigma_{0}^{\#}$

$\sigma_{y}^{'}= \frac{\sigma_{y_{0}}}{\sigma_{0}^{\#}}\frac{\exp\left[ \left( \frac{-\left( y^{'}- y_{0}^{'} \right)^{2}}{2{[y_{1}^{'}/(y^{'}+ y_{2}^{'})]}^{2y_{4}^{'}}} \right) \right]- y_{3}^{'}}{1- y_{3}^{'}}$ (58)

$\sigma_{0}^{\#}$ is normalization constant and $y_{0}^{'}, y_{1}^{'}, y_{2}^{'},y_{3}^{'},y_{4}^{'}, u_{1}^{'}, u_{2}^{'}, u_{3}^{'}, x_{0}, x_{1}^{'}, x_{2}^{'}, x_{3}^{'}, x_{4}^{'}, x_{5}^{'}, x_{6}^{'}, x_{7}^{'}$are constants.

*(g) Normalized contractile component of length, y’*

$\frac{\boldsymbol{d}\boldsymbol{y}^{\boldsymbol{'}}}{\boldsymbol{dt}}\boldsymbol{=}\left\{ \begin{matrix} \boldsymbol{-}\boldsymbol{v}_{\boldsymbol{ref}}^{\boldsymbol{'}}\frac{\boldsymbol{\Psi}}{\boldsymbol{\Psi}_{\boldsymbol{ref}}}\boldsymbol{a}^{\boldsymbol{'}}\frac{\boldsymbol{1-}\frac{\boldsymbol{\sigma}_{\boldsymbol{u}}^{\boldsymbol{'}}}{\boldsymbol{\sigma}_{\boldsymbol{y}}^{\boldsymbol{'}}}}{\boldsymbol{a}^{\boldsymbol{'}}\boldsymbol{+}\frac{\boldsymbol{\sigma}_{\boldsymbol{u}}^{\boldsymbol{'}}}{\boldsymbol{\sigma}_{\boldsymbol{y}}^{\boldsymbol{'}}}}\boldsymbol{, 0\leq}\frac{\boldsymbol{\sigma}_{\boldsymbol{u}}^{\boldsymbol{'}}}{\boldsymbol{\sigma}_{\boldsymbol{y}}^{\boldsymbol{'}}}\boldsymbol{\leq1} \\ \boldsymbol{c}^{\boldsymbol{'}}\left[ \boldsymbol{exp}\left( \boldsymbol{b}^{\boldsymbol{'}}\left( \frac{\boldsymbol{\sigma}_{\boldsymbol{u}}^{\boldsymbol{'}}}{\boldsymbol{\sigma}_{\boldsymbol{y}}^{\boldsymbol{'}}}\boldsymbol{-}\boldsymbol{d}^{\boldsymbol{'}} \right) \right)\boldsymbol{- exp(}\boldsymbol{b}^{\boldsymbol{'}}\left( \boldsymbol{1-}\boldsymbol{d}^{\boldsymbol{'}} \right)\boldsymbol{)} \right]\boldsymbol{, 1\leq}\frac{\boldsymbol{\sigma}_{\boldsymbol{u}}^{\boldsymbol{'}}}{\boldsymbol{\sigma}_{\boldsymbol{y}}^{\boldsymbol{'}}} \end{matrix} \right.$ (59)

$\sigma_{y_{0}}= \sigma_{y_{0}}^{\#}\omega/\omega_{ref}$ (60)

where $\omega_{ref}= \Psi({Ca}_{ref})/(\Psi_{m}+\Psi[{Ca}^{2+}]_{SMC,ref}))$

a’, b’, c’ and d’ are constants, v is velocity of contraction of the contractile component at zero load, and $v^{'}=v/x_{0}$, with $v_{ref}^{'}$ is $v^{'}$ at the reference muscle activation level.
