# Supplementary material for: Grey-box modeling and hypothesis testing of functional near-infrared spectroscopy-based cerebrovascular reactivity to anodal high-definition tDCS in healthy humans
Source: PLoS Comput Biol. 2021 Oct 6;17(10):e1009386. doi: 10.1371/journal.pcbi.1009386 (PMC8494321; doi:10.1371/journal.pcbi.1009386)
Supplement: S2 Text. Transcranial electrical stimulation induced current density in the neurovascular cortical tissue — (DOCX) [file pcbi.1009386.s006.docx]

**S2 Text: Transcranial electrical stimulation induced current density in the neurovascular cortical tissue**

Maxwell equations calculate and describe the electric and magnetic fields originated by distributions of electric charges and currents. The differentiation notation of Maxwell equations is as follows where the electric force fields are described by the electric field E and the electric flux density D = εE; that is how the electrical charges in a material become polarized in an electric field. The magnetic force fields are described by the magnetic field H and the magnetic flux density B = µH, which is accounting for the magnetization of a material. Here, ε is permittivity, µ is permeability, $J_{tDCS}$ is current density, and $\rho$ is electric charge density.

$\left\{ \begin{aligned} \nabla\times\vec{E}=-\frac{\partial\vec{B}}{\partial t} \\ \nabla.\vec{D}=\rho\\ \nabla\times\vec{H}=-\vec{J_{tDCS}}+\frac{\partial\vec{D}}{\partial t} \\ \nabla.\vec{B}=0 \\ \end{aligned} \right.$ (61)

For electrostatics in any medium, two following equation from Maxwell equations are applied:

$\nabla.\vec{D}=\rho$ (62)

$\nabla\times\vec{E}=0$ (63)

Because of the irrotational nature of E (Eq.3), we can define a scalar electric potential V as:

$\vec{E}=-\nabla V (\frac{V}{m})$ (64)

The negative sign shows that E and V are in the opposite direction.

On the other hand, the equation for current density ($J_{tDCS}$) in unit volume (units A/m^3^) based on the principle of conservation of charge is: (‘*equation of continuity’*)

$\nabla.\vec{J_{tDCS}}=-\frac{\partial\rho}{\partial t}$ (65)

For steady current ($\frac{\partial\rho}{\partial t}=0$) or when there is no flow source ($\rho=0$ ), the equation will be:

$\nabla.\vec{J_{tDCS}}=0$ (66)

For an Ohmic material, we have:

$\vec{J_{tDCS}}=\sigma\vec{E}$ (A/m^2^) (67)

Where, $\sigma\left( \frac{A}{V.m} \right)or \left( \frac{S}{m} \right)$ is the conductivity tensor of the Ohmic medium.
