# Supplementary material for: Grey-box modeling and hypothesis testing of functional near-infrared spectroscopy-based cerebrovascular reactivity to anodal high-definition tDCS in healthy humans
Source: PLoS Comput Biol. 2021 Oct 6;17(10):e1009386. doi: 10.1371/journal.pcbi.1009386 (PMC8494321; doi:10.1371/journal.pcbi.1009386)
Supplement: S1 Table — (DOCX) [file pcbi.1009386.s007.docx]

| Compartment | Parameter | Value | Unit |
| --- | --- | --- | --- |
| Synaptic Space | $J_{\Sigma Kmax}*k_{Na}$ | 0.6 | mM/s |
|  | ${KKO}_{a}$ | 1.5 | mM |
|  | $K_{G}$ | 8.82 | - |
|  | $\delta$ | 0.001235 | - |
| Astrocytic Intracellular Space | $r_{h}$ | 4.8 | μM |
|  | $k_{deg}$ | 1.25 | s^-1^ |
|  | $K_{Glu}$ | 1 | mM |
|  | $\beta$ | 0.0244 | - |
|  | $J_{max}$ | 2880 | μM/s |
|  | $K_{I}$ | 0.03 | μM |
|  | $K_{act}$ | 0.17 | μM |
|  | $V_{max}$ | 20 | μM/s |
|  | K_p_ | 0.24 | μM |
|  | $P_{L}$ | 5.2 | μM |
|  | $\left[ {Ca}^{2+} \right]_{ER}$ | 400 | μM |
|  | $C_{astr}$ | 40 | pF |
|  | $g_{TRPV}$ | 200 | pS |
|  | $v_{TRPV}$ | 6 | mV |
|  | $k_{on}$ | 2 | μM^-1^s^-1^ |
|  | $K_{inh}$ | 0.1 | μM |
|  | $\tau_{TRPV}$ | 0.9 | s^-1^ |
|  | $\varepsilon_{1/2}$ | 0.16 | - |
|  | $\kappa$ | 0.04 | - |
|  | $\gamma_{{Ca}_{i}}$ | 0.2 | μM |
|  | $\gamma_{{Ca}_{e}}$ | 0.2 | mM |
|  | $v_{1,TRPV}$ | 120 | mV |
|  | $v_{2,TRPV}$ | 13 | mV |
|  | $V_{EET}$ | 72 | s^-1^ |
|  | $\left[ {Ca}^{2+} \right]_{min}$ | 0.1 | μM |
|  | $k_{EET}$ | 7.1 | s^-1^ |
|  | $\Psi_{BK}$ | 2.664 | s^-1^ |
|  | $v_{4,BK}$ | 14.5 | mV |
|  | $v_{5,BK}$ | 8 | mV |
|  | $v_{6,BK}$ | -15 | mV |
|  | ${EET}_{shift}$ | 2 | mV/μM |
|  | ${Ca}_{3,BK}$ | 400 | nM |
|  | ${Ca}_{4,BK}$ | 150 | nM |
|  | $g_{BK}$ | 225.6 | pS |
|  | $v_{BK}$ | -95 | mV |
|  | $g_{leak}$ | 78.54 | pS |
|  | $v_{leak}$ | -70 | mV |
| Perivascular Space | $\left[ {Ca}^{2+} \right]_{P,min}$ | 2000 | μM |
|  | ${[K^{+}]}_{P,min}$ | 3 | mM |
|  | ${Ca}_{decay}$ | 0.5 | s^-1^ |
|  | $R_{decay}$ | 1 | s^-1^ |
|  | ${VR}_{pa}$ | 3.2e-5 | - |
| Arteriole Smooth Muscle Cell (SMC) Intracellular Space | $\tau$ | 0.2 | dyne/cm |
|  | $\Delta_{p}$ | 60 | mmHg |
|  | $w_{e}$ | 0.9 | - |
|  | A | 502.65 | μm^2^ |
|  | $S$ | 40000 | μm^2^ |
|  | $w_{m}$ | 0.7 | - |
|  | $\sigma_{0}^{\#}$ | 3e+6 | dyne/cm^2^ |
|  | $x_{0}$ | 188.5 | μm |
|  | $x_{1}^{'}$ | 1.2 | - |
|  | $x_{2}^{'}$ | 0.13 | - |
|  | $x_{3}^{'}$ | 2.2443 | - |
|  | $x_{4}^{'}$ | 0.71182 | - |
|  | $x_{5}^{'}$ | 0.8 | - |
|  | $x_{6}^{'}$ | 0.01 | - |
|  | $x_{7}^{'}$ | 0.32134 | - |
|  | $x_{8}^{'}$ | 0.88977 | - |
|  | $x_{9}^{'}$ | 0.0090463 | - |
|  | $u_{1}^{'}$ | 41.76 | - |
|  | $u_{2}^{'}$ | 0.047396 | - |
|  | $u_{3}^{'}$ | 0.0584 | - |
|  | $y_{0}^{'}$ | 0.928 | - |
|  | $y_{1}^{'}$ | 0.639 | - |
|  | $y_{2}^{'}$ | 0.35 | - |
|  | $y_{3}^{'}$ | 0.78847 | - |
|  | $y_{4}^{'}$ | 0.8 | - |
|  | $C_{SMC}$ | 19.635 | pF |
|  | $g_{KIR,0}$ | 145 | pS |
|  | $v_{KIR,1}$ | 57 | mV |
|  | $v_{KIR,2}$ | 130 | mV |
|  | $\gamma$ | 1970 | mV/μM |
|  | $g_{Ca}$ | 157 | pS |
|  | $v_{Ca}$ | 80 | mV |
|  | $v_{1}$ | -21 | mV |
|  | $v_{2}$ | 25 | mV |
|  | $\alpha_{KIR}$ | 1020 | S |
|  | $a_{v_{1}}$ | 18 | mV |
|  | $a_{v_{2}}$ | 10.8 | mV |
|  | $\beta_{KIR}$ | 26.9 | S |
|  | $b_{v_{1}}$ | 18 | mV |
|  | $b_{v_{1}}$ | 0.06 | mV |
|  | $g_{K}$ | 251.33 | pS |
|  | $v_{k}$ | -80 | mV |
|  | $g_{L}$ | 62.832 | pS |
|  | $v_{l}$ | -70 | mV |
|  | $\phi_{n}$ | 2.664 | - |
|  | ${Ca}_{3}$ | 400 | nM |
|  | ${Ca}_{4}$ | 150 | nM |
|  | $v_{4}$ | 14.5 | mV |
|  | $v_{5}$ | 8 | mV |
|  | $v_{6}$ | -15 | mV |
|  | $K_{d}$ | 1000 | nM |
|  | $B_{T}$ | 10000 | nM |
|  | $\alpha$ | 4.3987e+15 | nM/C |
|  | $k_{ca}$ | 1.3568e+2 | s^-1^ |
|  | $k_{\Psi}$ | 3.3 | - |
|  | Ca_m_ | 500 | nM |
|  | $[{Ca}^{2+}]_{SMC,ref}$ | 510 | nM |
|  | $\Psi_{m}$ | 0.3 | - |
|  | Q | 3 | - |
|  | $\sigma_{y_{0}}^{\#}$ | 2.6e+6 | dyne/cm^2^ |
|  | v_ref_ | 0.24 | - |
|  | $a^{'}$ | 0.28125 | - |
|  | $b^{'}$ | 5 | - |
|  | $c^{'}$ | 0.03 | - |
|  | $d^{'}$ | 1.3 | - |
