# Supplementary material for: Grey-box modeling and hypothesis testing of functional near-infrared spectroscopy-based cerebrovascular reactivity to anodal high-definition tDCS in healthy humans
Source: PLoS Comput Biol. 2021 Oct 6;17(10):e1009386. doi: 10.1371/journal.pcbi.1009386 (PMC8494321; doi:10.1371/journal.pcbi.1009386)
Supplement: S2 Table — Normalized Oxy-hemoglobin (oxy-Hb), Deoxy-hemoglobin (deoxy-Hb) and Total Hemoglobin (tHb) obtained from NIRS channel in tDCS stimulated region for all the 11 participants. *subjects with >-0.5 Correlation Coefficient between Oxy-Hb & Dxy-Hb. (DOCX) [file pcbi.1009386.s008.docx]

The changes in oxy-, deoxy- and total hemoglobin obtained through fNIRS channel placed on the tDCS stimulated region for every participant is presented in the following table. Here, pre-tDCS baseline (mean value) was subtracted and then the time course was normalized with the maximum (the maximum value gets transformed into a 1) for the changes in the oxy, deoxy and total hemoglobin concentration during the entire stimulation. The normalized time courses are presented for comparison during tDCS stimulation period as the continuous wave (CW)-fNIRS system used in the experimental setup provided relative values.

| Sub # | Normalized oxy-hemoglobin and deoxy-hemoglobin | Correlation Coefficient between oxy-Hb & deoxy-Hb | Total Hemoglobin, tHb (Normalized) |
| --- | --- | --- | --- |
| 1 | 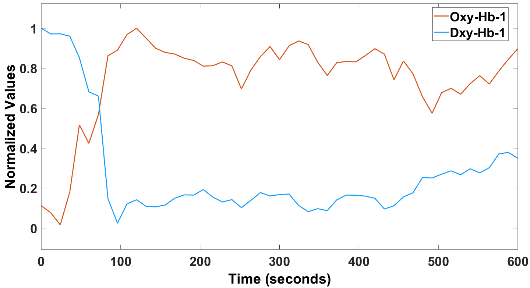 | -0.9007 | 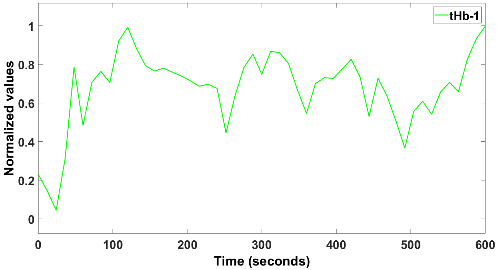 |
| 2 | 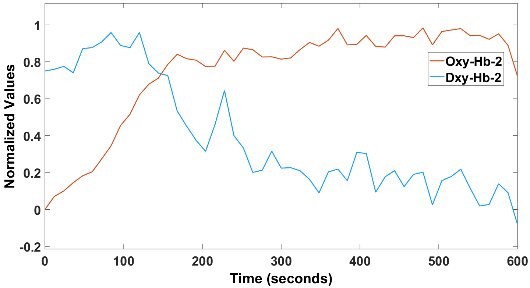 | -0.7923 | 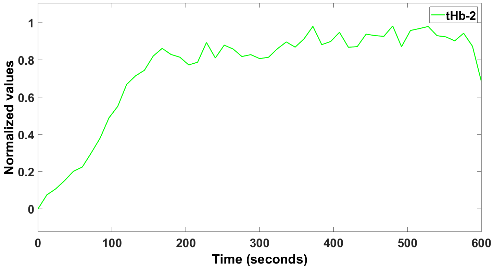 |
| 3* | 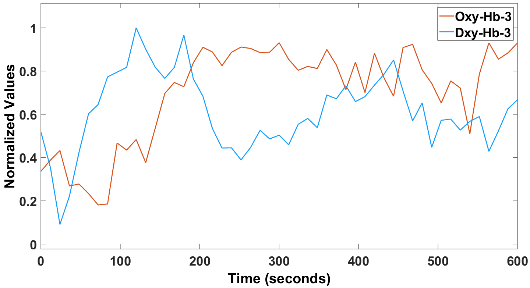 | -0.0692 | 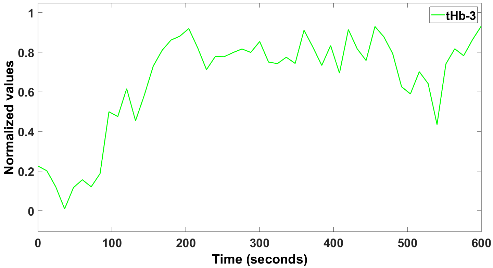 |
| 4* | 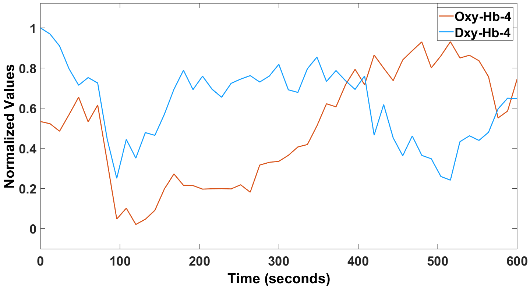 | -0.1900 | 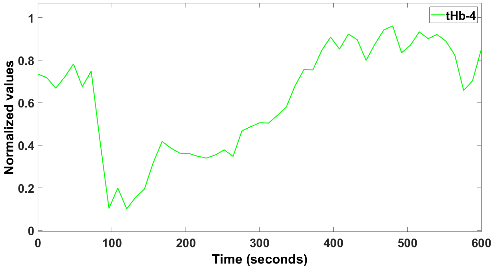 |
| 5 | 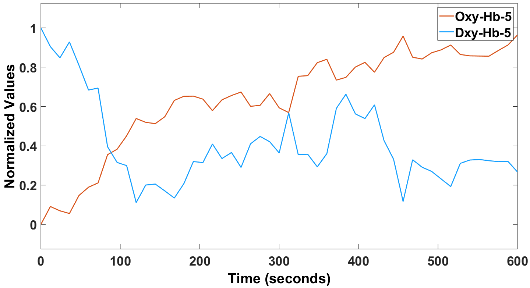 | -0.6735 | 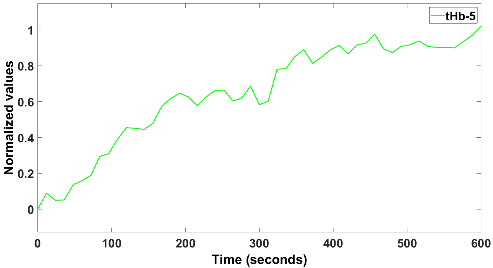 |
| 6 | 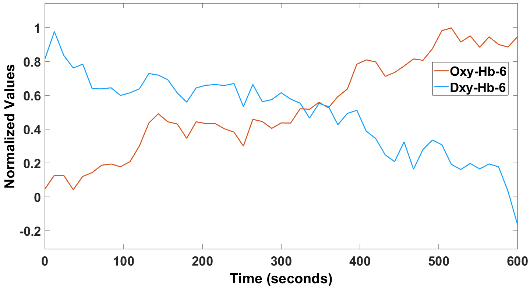 | -0.8683 | 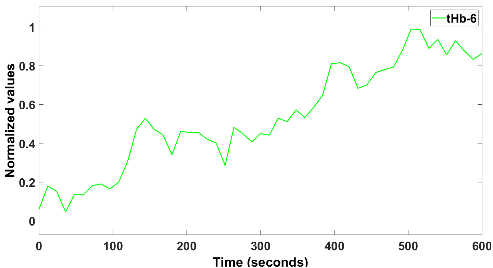 |
| 7 | 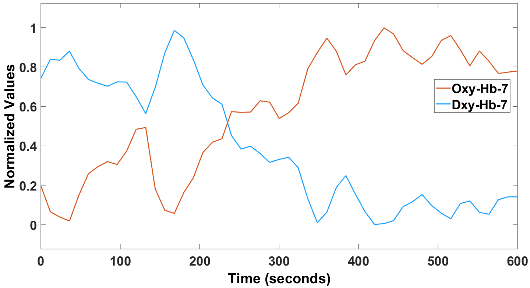 | -0.9759 | 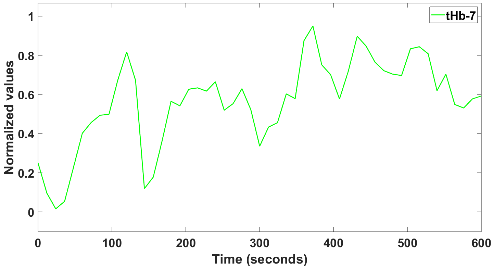 |
| 8 | 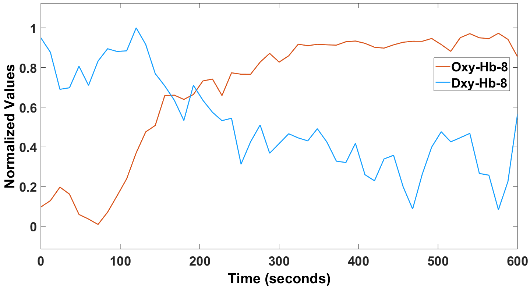 | -0.8390 | 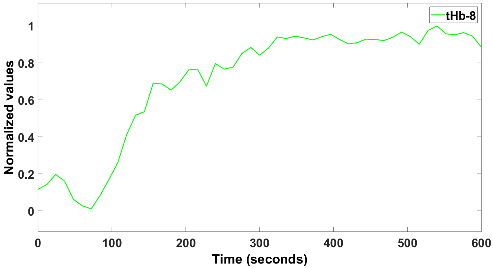 |
| 9 | 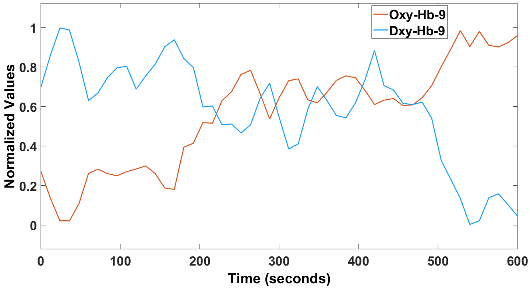 | -0.8542 | 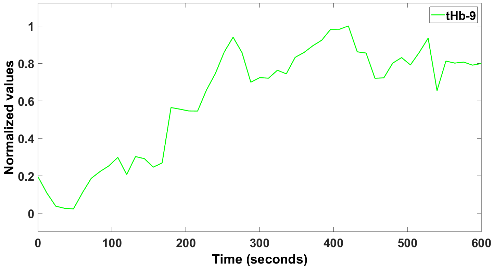 |
| 10* | 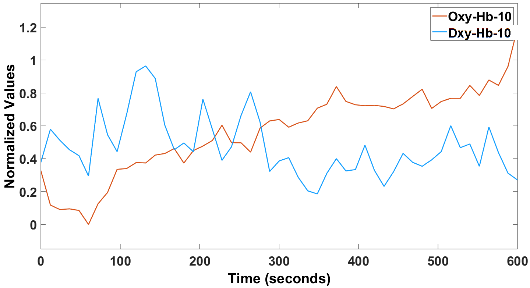 | -0.3768 | 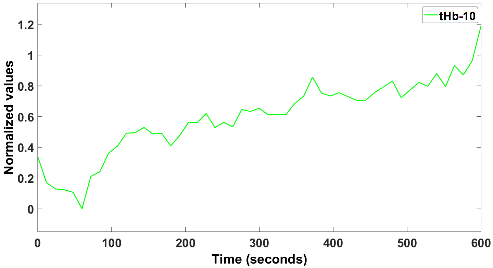 |
| 11 | 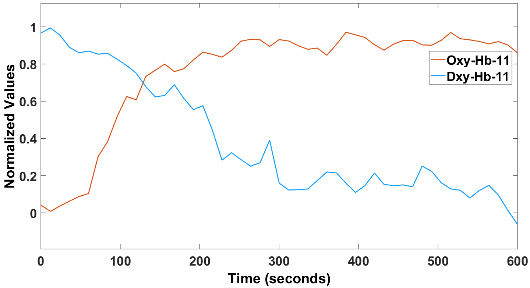 | -0.8691 | 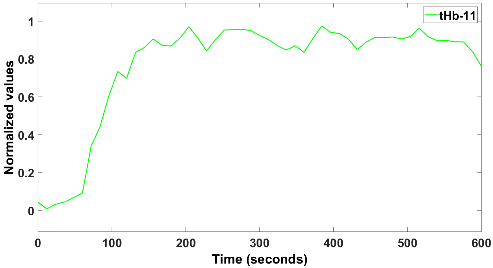 |
