# Supplementary material for: Grey-box modeling and hypothesis testing of functional near-infrared spectroscopy-based cerebrovascular reactivity to anodal high-definition tDCS in healthy humans
Source: PLoS Comput Biol. 2021 Oct 6;17(10):e1009386. doi: 10.1371/journal.pcbi.1009386 (PMC8494321; doi:10.1371/journal.pcbi.1009386)
Supplement: S3 Table — System identification using experimental data (total hemoglobin changes from stimulated hemisphere) from 11 subjects (Sub). * subjects with >-0.5 Correlation Coefficient between Oxy-Hb & Dxy-Hb. (DOCX) [file pcbi.1009386.s009.docx]

System identification toolbox (MathWorks, Inc., USA) was used with the time-domain input-output data from eleven healthy participants. The values of the free parameters (poles and zeros) of the parameterized grey-box linear model was updated using the “Refine Existing Model” approach in the System identification toolbox to improve the fit to the estimation data. In this method, linear grey-box model obtained following linearization of physiologically detailed model using Simulink’s (MathWorks, Inc., USA) linear analysis tool was used to fit the experimental fNIRS-tDCS data from each healthy participant. The identified systems for the four pathways using each healthy participant data are given in the following table.

| **Pathway** | **Sub#** | **System identification using experimental data** |
| --- | --- | --- |
| 1: Synaptic Potassium → vessel circumference | 1 | 7.2083e-09 (s+5.055e07) (s+9.594e06) (s+2.974e04) (s+1648) (s+50) (s+20.69)(s+15.08) (s+3.836) (s+0.003298) (s^2^ + 9.803s + 95.24) /  (s+9.594e06) (s+2.974e04) (s+50) (s+20.69) (s+15.08) (s+6.852) (s+0.05234)(s+0.005084) (s^2^ + 2.415e-08s + 15.47)(s^2^ + 9.805s + 95.26) |
|  | 2 | 4.822e-09 (s+9.796e07) (s+9.594e06) (s+2.974e04) (s-1237) (s+50) (s+20.69) (s+15.08) (s+3.857) (s-0.7089) (s^2^ + 9.803s + 95.24) /  (s+9.594e06) (s+2.974e04) (s+50) (s+20.69) (s+15.08) (s+0.1084)  (s^2^ + 6.595s + 11.29) (s^2^ + 0.2092s + 2.423) (s^2^ + 9.802s + 95.29) |
|  | 3* | 5.9575e-08 (s+9.594e06) (s+7.211e06) (s+2.974e04) (s+5472) (s+50) (s+20.69) (s+15.08) (s+3.588) (s+2.638) (s^2^ + 9.804s + 95.24) /  (s+9.594e06) (s+2.974e04) (s+50) (s+20.69) (s+15.08) (s+0.07776)  (s^2^ + 0.01736s + 12.99) (s^2^ + 1.421s + 44.43) (s^2^ + 15.2s + 103.7) |
|  | 4* | 4.6202e-08 (s+9.594e06) (s+9.058e06) (s+2.974e04) (s+596) (s+50) (s+20.69) (s+15.08) (s+3.648) (s+0.01155) (s^2^ + 9.804s + 95.24) /  (s+9.594e06) (s+2.974e04) (s+50) (s+20.69) (s+15.08) (s+0.002497)  (s^2^ + 8.485e-07s + 0.4953) (s^2^ + 6.908s + 15.38) (s^2^ + 9.804s + 95.24) |
|  | 5 | 1.0846e-08 (s+3.876e07) (s+9.594e06) (s+2.974e04) (s+80.58) (s+50) (s+20.69) (s+15.08) (s+3.823) (s-7.563e-05) (s^2^ + 9.803s + 95.24) /  (s+9.594e06) (s+2.974e04) (s+50) (s+20.69) (s+15.08) (s+1.722e-05)  (s^2^ + 0.276s + 0.02161) (s^2^ + 6.635s + 14.04) (s^2^ + 9.804s + 95.24) |
|  | 6 | 8.4064e-09 (s+4.164e07) (s+9.594e06) (s+2.974e04) (s+507.6) (s+50) (s+20.69) (s+15.08) (s+3.828) (s+0.009477) (s^2^ + 9.803s + 95.24) /  (s+9.594e06) (s+2.974e04) (s+50) (s+20.69) (s+15.08) (s+6.532) (s+0.06082) (s+0.0003971) (s^2^ + 0.4731s + 22.34)(s^2^ + 9.649s + 85.98) |
|  | 7 | 8.2796e-10 (s+4.772e08) (s+9.594e06) (s+2.974e04) (s+1616) (s+50) (s+20.69) (s+15.08) (s+3.875) (s+0.04567) (s^2^ + 9.803s + 95.24) /  (s+9.594e06) (s+2.974e04) (s+50) (s+20.69) (s+15.08) (s+6.809)  (s^2^ + 0.09585s + 0.002608) (s^2^ + 2.217e-08s + 14.98) (s^2^ + 9.81s + 95.48) |
|  | 8 | 1.1834e-08 (s+3.708e07) (s+9.594e06) (s+2.974e04) (s-4498) (s+50) (s+20.69) (s+15.08) (s+3.8) (s-1.758) (s^2 + 9.803s + 95.24) / (s+9.594e06) (s+2.974e04) (s+50) (s+20.69) (s+15.08) (s+0.07725)  (s^2^ + 0.007259s + 12.8) (s^2^ + 0.001041s + 18.85) (s^2^ + 16.63s + 150) |
|  | 9 | 1.5696e-08 (s+2.334e07) (s+9.594e06) (s+2.974e04) (s+50) (s+20.69) (s+18.98) (s+15.08) (s+3.786) (s+0.03044) (s^2^ + 9.803s + 95.24) /  (s+9.594e06) (s+2.974e04) (s+50) (s+20.69) (s+15.08) (s+0.003341)  (s^2^ + 0.07064s + 0.03265) (s^2^ + 6.837s + 15.35) (s^2^ + 9.804s + 95.24) |
|  | 10* | 1.2334e-08 (s+3.056e07) (s+9.594e06) (s+2.974e04) (s+4715) (s+50) (s+20.69) (s+15.08) (s+3.809) (s+0.111) (s^2^ + 9.803s + 95.24) /  (s+9.594e06) (s+2.974e04) (s+50) (s+20.69) (s+15.08) (s+3.541) (s+0.2731) (s+0.04935) (s^2^ + 12.52s + 78.44) (s^2^ + 0.3272s + 50.04) |
|  | 11 | -1.2815e-08 (s-3.273e07) (s+9.594e06) (s-4.243e04) (s+2.974e04) (s+50) (s+20.69) (s+15.08) (s+3.815) (s-1.034) (s^2^ + 9.803s + 95.24) /  (s+9.594e06) (s+2.974e04) (s+50) (s+20.69) (s+15.08) (s+0.08326) (s^2^ + 0.003256s + 22.73) (s^2^ + 3.299e-05s + 38.62) (s^2^ + 16.63s + 200.9) |
| **Pathway** | **Sub#** | **System identification using experimental data** |
| 2: Astrocytic Current channel → vessel circumference | 1 | -5.3726e-06 (s+9.594e06) (s-6.453e05) (s+2.974e04) (s+917.7) (s+50) (s+20.69) (s+15.08) (s+0.6542) (s^2^ + 9.785s + 95.61) /  (s+9.594e06) (s+2.974e04) (s+50) (s+20.69) (s+15.08) (s+11.63) (s+0.03122) (s^2^ + 0.07727s + 24.06) (s^2^ + 4.578s + 93.38) |
|  | 2 | 5.5342e-05 (s+9.594e06) (s+2.974e04) (s+50) (s+20.69) (s+15.08) (s+1.443) (s^2^ + 9.779s + 95.52) (s^2^ + 5.64e04s + 6.499e10) /  (s+9.594e06) (s+2.974e04) (s+50) (s+20.69) (s+15.08) (s+8.423) (s+0.1029) (s^2^ + 0.002347s + 90.08) (s^2^ + 7.79s + 1.131e04) |
|  | 3* | 3.6864e-07 (s+1.048e07) (s+9.594e06) (s+2.974e04) (s-3365) (s+50) (s+20.69) (s+15.08) (s-1.143) (s^2^ + 9.808s + 95.37) /  (s+9.594e06) (s+2.974e04) (s+50) (s+20.69) (s+16.15) (s+15.08) (s+0.1192) (s^2^ + 0.03886s + 8.511) (s^2^ + 4.124e-08s + 159.9) |
|  | 4* | -2.0486e-06 (s+9.594e06) (s-1.855e06) (s+2.974e04) (s+2.471e04) (s+50) (s+20.69) (s+15.08) (s+0.004899) (s^2^ + 9.799s + 95.46) /  (s+9.594e06) (s+2.974e04) (s+50) (s+20.69) (s+16.12) (s+15.09) (s+0.002483) (s^2^ + 0.1811s + 13.56) (s^2^ + 1.997e-05s + 153.9) |
|  | 5 | -5.8294e-06 (s+9.594e06) (s-6.845e05) (s+2.974e04) (s+5377) (s+50) (s+20.69) (s+15.08) (s+0.07949) (s^2^ + 9.786s + 95.6) /  (s+9.594e06) (s+2.974e04) (s+50) (s+20.69) (s+15.08) (s+12.24) (s+0.03593) (s^2^ + 4.03s + 6.88) (s^2^ + 0.003999s + 115.6) |
|  | 6 | -4.6967e-06 (s+9.594e06) (s-7.084e05) (s+2.974e04) (s+909.7) (s+50) (s+20.69) (s+15.08) (s+0.04692) (s^2^ + 9.787s + 95.59) /  (s+9.594e06) (s+2.974e04) (s+50) (s+20.69) (s+15.08) (s+13.11) (s+2.997) (s+0.1655) (s+0.03738) (s^2^ + 1.265e-07s + 130) |
|  | 7 | -6.7038e-06 (s+9.594e06) (s-5.678e05) (s+2.974e04) (s-1000) (s+50) (s+20.69) (s+15.08) (s-0.77) (s^2^ + 9.781s + 95.64) /  (s+9.594e06) (s+2.974e04) (s+50) (s+20.69) (s+0.02738)  (s^2^ + 31.21s + 243.8) (s^2^ + 0.1505s + 36.5) (s^2^ + 0.006018s + 46.08) |
|  | 8 | -5.2665e-06 (s+9.594e06) (s-7.883e05) (s+2.974e04) (s-1217) (s+50) (s+20.69) (s+15.08) (s-4.209) (s^2^ + 9.789s + 95.57) /  (s+9.594e06) (s+2.974e04) (s+50) (s+20.69) (s+15.96) (s+15.05) (s+0.104)  (s^2^ + 4.776e-07s + 13.07) (s^2^ + 0.2827s + 161.4) |
|  | 9 | -4.1814e-06 (s+9.594e06) (s-8.233e05) (s+2.974e04) (s-4117) (s+50) (s+20.69) (s+15.08) (s-0.4122) (s^2^ + 9.79s + 95.56) /  (s+9.594e06) (s+2.974e04) (s+50) (s+20.69) (s+16.11) (s+15.09) (s+0.0637) (s^2^ + 0.1327s + 11.1) (s^2^ + 0.0002993s + 157) |
|  | 10 | -4.7184e-06 (s+9.594e06) (s-7.556e05) (s+2.974e04) (s-5823) (s+50) (s+20.69) (s+15.08) (s-2.034) (s^2^ + 9.788s + 95.58) /  (s+9.594e06) (s+2.974e04) (s+50) (s+20.69) (s+15.87) (s+15.01) (s+0.1194) (s^2^ + 0.0389s + 29.51) (s^2^ + 0.3488s + 154.2) |
|  | 11 | -5.7775e-06 (s+9.594e06) (s-7.224e05) (s+2.974e04) (s+271.3) (s+50)  (s+20.69) (s+15.08) (s+0.03232) (s^2^ + 9.787s + 95.58) /  (s+9.594e06) (s+2.974e04) (s+50) (s+20.69) (s+15.08) (s+0.1171) (s+0.02904) (s^2^ + 8.167e-09s + 11.07) (s^2^ + 16.17s + 158.9) |
| **Pathway** | **Sub#** | **System identification using experimental data** |
| 3: Perivascular Potassium → vessel circumference | 1 | -0.0001314 (s+9.594e06) (s-6.287e05) (s+2.974e04) (s+50) (s+24.3) (s+20.69) (s^2^ + 9.784s + 95.62) /  (s+9.594e06) (s+2.974e04) (s+50) (s+20.69) (s+10.07) (s+3.396) (s+0.1023) (s^2^ + 0.7806s + 104.5) |
|  | 2 | 7.1761e-06 (s+9.594e06) (s+7.948e06) (s+2.974e04) (s+50) (s+20.69) (s+0.2589) (s^2^ + 9.798s + 95.27) /  (s+9.594e06) (s+2.974e04) (s+50) (s+20.69) (s+0.01201)  (s^2^ + 1.818e-07s + 1.392) (s^2^ + 14.34s + 184.3) |
|  | 3* | -6.4407e-06 (s-1.418e07) (s+9.594e06) (s+2.974e04) (s+50) (s+20.69)  (s-0.0002072) (s^2^ + 9.806s + 95.39) /  (s+9.594e06) (s+2.974e04) (s+50) (s+20.69) (s+1.85e-07)  (s^2^ + 0.7226s + 0.1593) (s^2^ + 13.63s + 134.2) |
|  | 4* | -4.0486e-06 (s-2.217e07) (s+9.594e06) (s+2.974e04) (s+50) (s+20.69)  (s+0.2873) (s^2^ + 9.807s + 95.39) /  (s+9.594e06) (s+2.974e04) (s+50) (s+20.69) (s+0.1526)  (s^2^ + 0.9892s + 0.334) (s^2^ + 13.21s + 128.8) |
|  | 5 | -0.00036085 (s+9.594e06) (s-6.958e05) (s+2.974e04) (s+50) (s+20.71)  (s+6.755) (s^2^ + 10.47s + 95.46) /  (s+9.594e06) (s+2.974e04) (s+50) (s+20.7) (s+0.004357)  (s^2^ + 1.647s + 5.839) (s^2^ + 12.69s + 1.236e04) |
|  | 6 | -0.00011381 (s+9.594e06) (s-6.888e05) (s+2.974e04) (s+50) (s+20.69) (s+0.01007) (s^2^ + 9.786s + 95.59) /  (s+9.594e06) (s+2.974e04) (s+50) (s+20.69) (s+10.94) (s+0.07898) (s+0.0003096) (s^2^ + 3.334s + 106.9) |
|  | 7 | 3.979e-05 (s+9.594e06) (s-5.539e06) (s+2.974e04) (s+50) (s+20.69)  (s-26.62) (s^2^ + 9.78s + 95.01) /  (s+9.594e06) (s+2.974e04) (s+50) (s+20.7) (s+0.0299)  (s^2^ + 4.234s + 61.46) (s^2^ + 10.08s + 866.8) |
|  | 8 | -0.00012905 (s+9.594e06) (s-7.575e05) (s+2.974e04) (s+50) (s+20.69) (s+15.37) (s^2 + 9.788s + 95.58) /  (s+9.594e06) (s+2.974e04) (s+50) (s+20.69) (s+0.07885)  (s^2^ + 11.39s + 38.1)(s^2^ + 2.882s + 72.64) |
|  | 9 | -0.00010241 (s+9.594e06) (s-7.936e05) (s+2.974e04) (s+50) (s+20.69)  (s+0.02554) (s^2^ + 9.789s + 95.57) /  (s+9.594e06) (s+2.974e04) (s+50) (s+20.69) (s+7.556) (s+0.08572) (s+0.008279) (s^2^ + 6.699s + 92.43) |
|  | 10* | 4.2886e-05 (s+9.594e06) (s+5.559e05) (s+2.974e04) (s+50) (s+20.69) (s+0.3596) (s^2^ + 9.773s + 95.01) /  (s+9.594e06) (s+2.974e04) (s+50) (s+20.69) (s+0.006075)  (s^2^ + 0.3037s + 1.635) (s^2^ + 14.04s + 198.6) |
|  | 11 | 0.00065547 (s+9.594e06) (s-2.297e05) (s+2.974e04) (s+50) (s+20.69)  (s-0.2695) (s^2^ + 9.713s + 95.37) /  (s+9.594e06) (s+2.974e04) (s+50) (s+20.69) (s+9.175) (s+0.3927) (s+0.01357) (s^2^ + 4.764s + 192.3) |
| **Pathway** | **Sub#** | **System identification using experimental data** |
| 4: Voltage gated ion channel on smooth muscle cell → vessel circumference | 1 | -1384.1 (s+9.637e06) (s+47.75) (s+19.28) (s-1.495) (s^2^ - 1.579s + 5.417)/  (s+9.594e06) (s+50) (s+20.69) (s+3.306) (s+0.2176)  (s^2^ + 9.804s + 95.24) |
|  | 2 | -442.07 (s+9.782e06) (s+42.82) (s+16.87) (s-41.28) (s^2^ - 1.584s + 1.571)/  (s+9.594e06) (s+50) (s+20.69) (s+3.307) (s+0.214)  (s^2^ + 9.804s + 95.24) |
|  | 3* | -237.98 (s+9.853e06) (s-59.05) (s+43.7) (s+17.73) (s^2^ - 1.018s + 1.018)/  (s+9.594e06) (s+50) (s+20.69) (s+3.305) (s+0.221)  (s^2^ + 9.804s + 95.24) |
|  | 4* | -63371 (s+9.596e06) (s+49.84) (s+20.57) (s+5.68) (s-0.2409) (s+0.1248)/  (s+9.594e06) (s+50) (s+20.69) (s+3.323) (s+0.1037)  (s^2^ + 9.803s + 95.23) |
|  | 5 | -788.75 (s+9.678e06) (s+45.97) (s+18.37) (s-11.58) (s^2^ - 1.587s + 1.712)/  (s+9.594e06) (s+50) (s+20.69) (s+3.306) (s+0.2184)  (s^2^ + 9.804s + 95.24) |
|  | 6 | -164.87 (s+1.005e07) (s-117) (s+41.65) (s+16.78) (s^2^ - 1.243s + 1.207)/  (s+9.594e06) (s+50) (s+20.69) (s+3.306) (s+0.217) (s^2^ + 9.804s + 95.24) |
|  | 7 | -583.73 (s+9.718e06) (s+44.6) (s+17.73) (s-22.51) (s^2^ - 1.488s + 1.519)/  (s+9.594e06) (s+50) (s+20.69) (s+3.306) (s+0.2169)  (s^2^ + 9.804s + 95.24) |
|  | 8 | -71.969 (s+1.066e07) (s-282.5) (s+40.61) (s+16.47) (s^2^ - 1.234s + 1.185)/  (s+9.594e06) (s+50) (s+20.69) (s+3.306) (s+0.2166)  (s^2^ + 9.804s + 95.24) |
|  | 9 | -88213 (s+9.595e06) (s+49.86) (s+20.58) (s+5.842) (s+0.191) (s-0.2035)/  (s+9.594e06) (s+50) (s+20.69) (s+3.301) (s+0.2129)  (s^2^ + 9.803s + 95.23) |
|  | 10* | -338.73 (s+9.796e06) (s-43.91) (s+43.7) (s+17.54) (s^2^ - 1.222s + 1.212) /  (s+9.594e06) (s+50) (s+20.69) (s+3.306) (s+0.2188)  (s^2^ + 9.804s + 95.24) |
|  | 11 | -492.03 (s+9.747e06) (s+43.88) (s+17.42) (s-30.58) (s^2^ - 1.463s + 1.471)/  (s+9.594e06) (s+50) (s+20.69) (s+3.306) (s+0.2164)  (s^2^ + 9.804s + 95.24) |
