# Supplementary material for: Grey-box modeling and hypothesis testing of functional near-infrared spectroscopy-based cerebrovascular reactivity to anodal high-definition tDCS in healthy humans
Source: PLoS Comput Biol. 2021 Oct 6;17(10):e1009386. doi: 10.1371/journal.pcbi.1009386 (PMC8494321; doi:10.1371/journal.pcbi.1009386)
Supplement: S4 Table — * subjects with >-0.5 Correlation Coefficient between Oxy-Hb & Dxy-Hb. (DOCX) [file pcbi.1009386.s010.docx]

| **Sub #** | **Pathway 1 (Synaptic Potassium → vessel circumference)** | **Pathway 2 (Astrocytic membrane potential→ vessel circumference)** | **Pathway 3 (Perivascular Potassium → vessel circumference)** | **Pathway 4**  **(Voltage-gated ion channel on SMC → vessel circumference)** |
| --- | --- | --- | --- | --- |
| 1 | 0.03194 | 0.05235 | 0.2203 | 0.1321 |
| 2 | 0.1128 | 0.1422 | 0.004508 | 0.07534 |
| 3* | 0.08671 | 0.1937 | 0.05579 | 0.1598 |
| 4* | 0.01857 | 0.04674 | 0.07505 | 0.0679 |
| 5 | 0.06443 | 0.08512 | 0.005981 | 0.1466 |
| 6 | 0.008047 | 0.1361 | 0.008353 | 0.08054 |
| 7 | 0.06033 | 0.02859 | 0.02679 | 0.08053 |
| 8 | 0.09873 | 0.2548 | 0.3909 | 0.1242 |
| 9 | 0.00289 | 0.03508 | 0.01706 | 0.04097 |
| 10* | 0.05032 | 0.1326 | 0.00422 | 0.08931 |
| 11 | 0.05409 | 0.2009 | 0.004483 | 0.08612 |
