# Supplementary material for: Grey-box modeling and hypothesis testing of functional near-infrared spectroscopy-based cerebrovascular reactivity to anodal high-definition tDCS in healthy humans
Source: PLoS Comput Biol. 2021 Oct 6;17(10):e1009386. doi: 10.1371/journal.pcbi.1009386 (PMC8494321; doi:10.1371/journal.pcbi.1009386)
Supplement: Attachment — Submitted filename: Review_comments.pdf [file pcbi.1009386.s014.pdf]

Review

"Grey-box modeling and hypothesis testing of functional near-infrared spectroscopy based cerebrovascular reactivity to anodal high-definition tDCS in healthy humans"

The article describes a complete study about the effect of electrical stimulation on hemodynamics, more precisely vessel circumference and total hemoglobin, by means of a model –data comparison of 11 human participants. The original published physiological model include neuronal, astrocyte, perivascular and smooth muscle cells compartments tightly coupled.

The innovation of the study (apart from new data compared to the published pilot study) lies in the methodological part: The interesting biological point is that the authors highlight the four pathways by which electrical stimulation affects the cerebral blood flow via temporal dynamics of the vessel circumference. In order to better identify and understand these contributions separately, the authors choose to linearize and reduce the physiological model to transfer functions to obtain a grey-box linear model, and then compare it to individual participant's data and to the average.

In my opinion the definition of these four pathways and above all the model linearization and reduction are crucial points that make the strength of the idea and thus an interesting study to publish. Nonetheless, these methodological points are difficult to understand under the present form of the article: on one hand because of the organization of the document and on the other hand because of a lack precision about how are implemented the pathways (equations) and how is realized the post –processing of the model (linearization and reduction (Fig. 9B and 10), as indicated in the three first sections of this review.

I specify here that my review concerns essentially the model computing part of the article as I'm an expert neither in brain electrical stimulation nor experimental protocols.

Major issue #1: Reorganization of the manuscript

I know that guidelines of the journal encourage to write the results part before the methods one, but in this article, doing it the classical way would greatly improve the direct understanding of the framework imagined by par the authors. At the moment we begin to understand the approach only with Fig. 10 at first reading of the document.

In a new organization, I would keep content of Fig. 1 in the beginning with methods as I understood were acquired in a preceding pilot study and are thus here inputs to the questioning. Apart from the first §, sub-section A in methods section is the acquisition protocol and the most important part are the data used for the estimation/identification (Fig.1) thus the details of this section may be placed in the Suppl. Mat. to only keep the essentials parts in the main text, except details of processing concerning frequency matters. Also sub-section C

« physiologically detailed model linearization for grey-box analysis of experimental data » in results section contains in my opinion a majority of methodological content except the ultimate sentence so that its position in the manuscript should be reviewed.

If the authors want to keep the present organization, I strongly recommend to add a scheme of the whole framework in order to clarify the different parts of the manuscript.

Major issue #2: How is obtained Fig. 2B

Reading the 17 equations of the physiological model, I found that the effects contribute to vessel circumference x via the $\left[Ca^{2+}\right]$ concentration (or membrane potential V_m) in the SMC compartment. Therefore I don't catch how the authors created the separated contributions of the four pathways in Fig. 2B as they mentioned:

- First order function for each pathway in Fig. 9B leading to contribution variables ($\left[K^+\right]_S$, V_k, $\left[K^+\right]_P$, V_m) but a joined vessel function for x (as I suppose);
- "vasoactive signals for the pathways" in the ultimate § of sub-section (B) of the Results but without any reference to an equation or specification in the corresponding Fig. 2B caption;
- Confusing sentences in the section B of Suppl. Mat., highlighting the pathways: for instance "I_{KV} postulated to be driven by current density" means to me $I_{KV} = function\left(J_{tDCS}\right)$ and is coherent with the ultimate sentence about solving the model. Then how to obtain different contributions to variable x knowing that all finish in V_m equation with ($\left[K^+\right]_S$, V_k, $\left[K^+\right]_P$, V_m) variables strongly coupled?

  Another explanation relies later in the sentence as "a first order transfer function for its vasoactive signal means" which means to me $v_i = TF\left(J_{tDCS}\right)$. In this case, how are defined the different contributions $x_i = TF\left(v_i\right)$ of Fig. 2B?

I highly recommend to specify these points because it's a reproduction issue. Also Fig. 2B should be improved in terms of temporal resolution.

The question arising then if the four contributions lead to the same dimension, why making the reduction of the model and not just compare the MSE values to conclude about pathway 3 as the more important contribution ? This issue is also link to the improvement of methodological figures, as suggested in the next section.

Major issue #3: Clarification of Figures 9B and 10,

where a first order transfer function is mentioned both in each pathway without specifying if it's the same one or four different ones in Fig. 9B and *one* vasoactive signal applied to all pathways in Fig. 10. In the objective to help the reader to catch directly their idea, an illustrative figure would be a combination of these two figures which would reduce

discrepancies of series and parallel arrows, by adding the four intermediate variables names. Adding the variables names on the arrows and specify name or equations of function in the squares which would get rid of, among other, the discrepancy between the parallel aspect of Fig. 9B, the parallel one of Fig. 10 and the serial of the physiological model in Fig. 10.

The accompanied text should contain these equations i.e. Sub-Section B « pathways equations or models » of the Suppl. Mat. is important to better understand the approach because they highlight the link between “stimulation” variables and studied pathways variables. Thus, this section should be incorporated in the corresponding method section.

Major issue #4: section (D) participant-specific grey-box linear model dynamics

The use of the system identification toolbox of Matlab must be better explained in this section by specifying the variables obtained in the grey-box model ; And in the following section the variables ‘tfest’ obtained by the reduction named ‘balred’.

Minor issues: Overall review

The authors could improve the overall aspect and coherence of the manuscript by

- Reviewing typos : at the end of section titles, spaces after commas and full stops ;
- Choosing meaningful variable names as, for instance, $\left[Ca^{2+}\right]_A$ instead of $\left[Ca^{2+}\right]$ and keep it through the document. Another example is V_{SMC} instead of V_m which is generally taken for the membrane potential of neurons and not at the level of vessels;
- Also referring more often to the equations number helps the reader to follow the developed idea of the text and facilitate the possible jumps between linked sections, *including* in the figures captions;
- Adding pages number and lines facilitates reviewing and response to review.

Minor issue #1: Figures improvement

- Please increase the police size of labels if possible.
- Colors of Fig. 8 may be more segregating.
- Fig. 4 would benefit spatially and therefore gain sharpness to be organized (A and B) vertically instead of horizontally

Minor issue #2: Text improvement – example of sub-section C of the Methods section

A certain number of sentences could be rethink to be more pedagogical in the direction of *organization* of ideas inside a sub-section which also will avoid repetition. An example is the sentence "the TDCS current density in the brain's neurovascular tissue was assumed to be proportional…" and the same one "transcranial electrical stimulation induced current density … arbitrary gain" (around Eq. 1).

- Improvement of the vocabulary *precision*

  For remarkable example, the different occurrences of the term model (physiologically detailed model or physiological model, pathway models or grey-box model, …) to discriminate the steps of the study's framework should be chosen once and kept throughout the manuscript to strengthen the understanding of this crucial point of hypothesis testing). One occurrence in "Figure 10 shows …"

- Explanation of the Biological criteria in "We identified four nested ways …"
- *Structure* of sentences: be sure that sentences contain a verb (example "In pathway 1, the vasoactive …")

Minor issue #3: Table 3

The authors did a real effort in the bibliography of existing models summarized in this table. Still it can be easily improved by the following specifications:

- Replacing potassium by K+ everywhere ease to catch similarities across studies.
- NVU system 1: output = cerebral blood volume and cerebral blood flow
- NVU system 3: output = arteriolar radius
- NVU system 4: input = synaptic current, output = vessel radius via EET signaling
- NVU system 10: input = EPSP and FR from …
- NVU system 14: and astrocytes

Model number 9 in this table appears not interesting here to me as it doesn't have output at the level of vessels. I however suggest these references as models incorporating astrocytes and a neurovascular coupling input-output link:

- Riera, J et al. "Fusing EEG and fMRI based on a bottom-up model: inferring activation and effective connectivity in neural masses." *Philosophical transactions of the Royal Society of London. Series B, Biological sciences* vol. 360,1457 (2005): 1025-41.
- Riera JJ, Wan X, Jimenez JC, Kawashima R. Nonlinear local electrovascular coupling. I: A theoretical model. Hum Brain Mapp. 2006 Nov;27(11):896-914.

Without astrocytes:

- Banaji M, Mallet A, Elwell CE, Nicholls P, Cooper CE. A model of brain circulation and metabolism: NIRS signal changes during physiological challenges. PLoS Comput Biol. 2008 Nov;4(11):e1000212.

Minor issues #4: References

References to literature could be added throughout the manuscript. For instance

- First sentence of the abstract
- First § of the introduction about the three mechanisms of CBF regulation
- Second § of the introduction about effects of the synaptic projections, astrocytes and SMC on vessels after stimulation (done with perivascular compartment)
- Third § of the introduction for the sentence "as evident from studies"

Minor issues #5: Supplementary materials (Suppl. Mat.)

- Eqs. 21 and 26 and 41 of pathways variables should include the stimulation variables as in sub-section B (like in Eq. 1) with an accompanied sentence specifying them, to avoid confusion created by two different equations of the same variable
- Clarify the role of variable ω of Eq. 50 and that q is a power in Eq. 51, even if it's not specified as such in the original publication, as these variables make the link between vessels variable of interest x and the upstream dynamics
- Specify w_m of Eq. 55
- The sentence "and x = u + y " at the end of Eq. 55 is a complementary specification to Eq. 52, thus it should be reformulated to help understanding the link between x and ψ via Eq. 59
- No number is given to the "transcranial electrical stimulation" section. Besides, I don't see the reference of this section in the main text neither in the corresponding § of sub-section B of Suppl. Mat.
- The normalization taken for data *deoxy-Hb*, *oxy-Hb* and *tHb* in Table S2 should be explained.
- Table S1 could be organized in sub-tables (by compartment for instance) to facilitate the search of a particular parameter value
