# Supplementary material for: Grey-box modeling and hypothesis testing of functional near-infrared spectroscopy-based cerebrovascular reactivity to anodal high-definition tDCS in healthy humans
Source: PLoS Comput Biol. 2021 Oct 6;17(10):e1009386. doi: 10.1371/journal.pcbi.1009386 (PMC8494321; doi:10.1371/journal.pcbi.1009386)
Supplement: Attachment — Submitted filename: Review-PCOMPBIOL.docx [file pcbi.1009386.s013.docx]

**PLOS Computational Biology -Review**

**Grey-box modeling and hypothesis testing of functional near-infrared spectroscopybased cerebrovascular reactivity to anodal high-definition tDCS in healthy humans**

The authors have developed a grey-box linear model for an existing multicompartmental neurovascular unit model and used it to evaluate acute effects of anodal HD-tDCS for 11 healthy humans. Establishing theoretical frameworks to study experimental results is vital to understand the underlying physiological mechanisms. The authors have done a good job in simplifying complex models to investigate fNIRS based measure of blood volume particularly in response to HD-tDCS. Though the results are specific to the model of NVU chosen by the authors, the framework could be useful to test other hypotheses.

To understand the effects of tDCS on CVR, the oscillations in the frequency range 0.05 – 0.2 Hz must be considered in future studies. The authors could include some discussion on alternative approaches to the current framework to make that happen.

The article is well written and the method of using complex physiological models to understand experimental data has the potential to transform tDCS into a useful therapeutic technique. So, I recommend this paper to be accepted with minor revision.

**Fig 8:** what is the duration and method of stimulation in these three models? Based on these responses, how different do the authors think that the results will be if the model linearization and comparison is applied to the other two models.
