# Supplementary material for: Grey-box modeling and hypothesis testing of functional near-infrared spectroscopy-based cerebrovascular reactivity to anodal high-definition tDCS in healthy humans
Source: PLoS Comput Biol. 2021 Oct 6;17(10):e1009386. doi: 10.1371/journal.pcbi.1009386 (PMC8494321; doi:10.1371/journal.pcbi.1009386)
Supplement: Attachment — Submitted filename: Reviewers_response_PLOS_Final.docx [file pcbi.1009386.s015.docx]

**Reply to comments of Reviewer 1 (Review-PCOMPBIOL.docx)**

The authors have developed a grey-box linear model for an existing multi-compartmental neurovascular unit model and used it to evaluate acute effects of anodal HD-tDCS for 11 healthy humans. Establishing theoretical frameworks to study experimental results is vital to understand the underlying physiological mechanisms. The authors have done a good job in simplifying complex models to investigate fNIRS based measure of blood volume particularly in response to HD-tDCS. Though the results are specific to the model of NVU chosen by the authors, the framework could be useful to test other hypotheses.

-------------------------------------------------------------------------------------------------------

We thank the reviewer for his/her positive response. The point wise replies to the comments are given below:

**1. To understand the effects of tDCS on CVR, the oscillations in the frequency range 0.05 – 0.2 Hz must be considered in future studies. The authors could include some discussion on alternative approaches to the current framework to make that happen.**

***Authors.*** *Thanks, we have now included the discussion on this topic.*

*“The relation of the ∼0.1 Hz oscillatory vessel response vis-à-vis neuronal response may be related to the cortical excitability changes to anodal tDCS^98^ due to the involvement of calcium dynamics that needs further investigation using tACS. Unlike tDCS, tACS can lead to physiological entrainment at the frequency of stimulation for system identification that can provide physiological insights based on a physiologically detailed model (see the equations 48-49 in the Text S1). Also, longer duration tDCS is postulated to elevate extracellular K+ that can decrease calcium activity mediated by the inward rectifying potassium channel (see equations 36, 41 in the Text S1) in the mural cells where a combination of tDCS and tACS can be used for system identification. Besides inward rectifying potassium channel, voltage-dependent potassium channel, calcium activated potassium channel, ATP-activated potassium channels are also present in the mural cells that can interact with dilatory stress-induced calcium transients in the mural cells. Therefore, tES effects on the contractile mural cells that encircle the precapillary sphincter [28] at the transition between the penetrating arteriole and the first order capillary may be crucial for intracerebral neurocapillary modulation by tDCS^15^. These can be elucidated with multimodal optical imaging of neuronal, astrocytic calcium, and the hemodynamic neurovascular changes in an animal model for system identification.”*

**2. The article is well written and the method of using complex physiological models to understand experimental data has the potential to transform tDCS into a useful therapeutic technique. So, I recommend this paper to be accepted with minor revision.**

***Authors.*** *We thank the reviewer for their positive response.*

**3. Fig8: what is the duration and method of stimulation in these three models? Based on these responses, how different do the authors think that the results will be if the model linearization and comparison is applied to the other two models.**

***Authors.*** *Thanks for your concern. The normalized vessel response for the three models was simulated using the ‘ode23tb’ solver in Simulink (MathWorks, Inc., USA). For all the three models, a neuronal stimulus (model input) of duration 30 to 70 sec as depicted by shaded region in Fig 8 was considered.*

*We selected the physiologically detailed model by Witthoft and Karniadakis^38^ since their study showed the bidirectional communication between cerebral astrocytes and microvessels, which was relevant due to experimental results that astrocytes are susceptible to small variations in their membrane potential^92^, and their long processes are sensitive to polarization by tDCS^93-95^. Moreover, Witthoft and Karniadakis^38^ model had a quick start of the vasodilation comparable to Mathias et al.^83^ model that extended Kenny et al.^82^ model. It also captured the after-effect of the neuronal stimulation on the normalized vessel radius.*

*The model linearization of our model based on Witthoft and Karniadakis^38^ model generated a 11^th^ order transfer function for conventional neurovascular coupling pathway with quadratic terms with complex conjugate poles. For the other two models, the linearization would include reduced order transfer functions obtained by cascading various first order transfer functions depending on various state variables of the compartments. The model response obtained by implementing the equations in these models in MATLAB (Mathworks, Inc., USA) is given below.*

(a) *Kenny et al. model response during neuronal stimulus (start time: 30 second; end time: 70 second).*

(b) *Mathias et al., 2018 model response during neuronal stimulus (start time: 30 second; end time: 70 second).*

----------------------------------------------------------------------------------------------------------------

**Reply to comments of Reviewer 2 (View Letter.pdf)**

The author proposes a comprehensive review of NVC computational model and the evidence of modulatory consequences of tDCS on blood vessels. The paper is well written and easy to read, however, there are some points that need to be resolved. Here are some comments which may help the author to further improve the manuscript.

----------------------------------------------------------------------------------------------------------------

We thank the reviewer for his/her positive response. The point wise replies to the comments are given below:

**General Comments:**

**1- The study of NVC mechanisms requires concomitant measurements of changes in both neuronal activity and hemodynamic signals in response to a stimulus. In this respect, pyramidal cells have been identified as “neurogenic hubs” of NVC in the somatosensory cortex [1], and astrocytes have emerged as key players in the communication between activated neurons and blood vessels as recently reviewed [2]. tDCS-induced electric field effects on the hemodynamics via neuronal, astrocyte, and cerebral blood vessels, composed of pericytes, smooth muscle cells and endothelial cells.**

**[1] Lecrux, C., Toussay, X., Kocharyan, A., Fernandes, P., Neupane, S., Levesque, M., Plaisier, F., Shmuel, A., Cauli, B., Hamel, E., 2011. Pyramidal neurons are “neurogenic hubs” in the neurovascular coupling response to whisker stimulation. J. Neurosci. 31, 9836–9847.**

**[2] Cauli, B., Hamel, E., 2018. Brain perfusion and astrocytes. Trends Neurosci. 41, 409–413.**

**The authors postulate “that the immediate hemodynamic response^9^ based on CVR to short duration tDCS can provide a marker of blood vessels' capacity to dilate that can be hampered in various cerebrovascular diseases^36,37^. However, longer duration (>9 min) tDCS will induce neuroplastic changes^24^ where non-linear bidirectional neurovascular interactions^38^ can make the mechanistic models too complex for fNIRS data fitting and hypothesis testing.”**

**To which compartment would this short duration tDCS be linked?**

***Authors.*** *Thanks for your insights. Indeed, pyramidal cells have been identified as “neurogenic hubs” and we have now clarified this in the Introduction section:*

*“An increase in oxy-Hb immediately following tDCS may be explained through a feedforward "neurogenic hypothesis," whereby the direct neuronal modulation by tDCS causes a discharge of various vasoactive agents and an increase in oxy-Hb^49^. Here, autonomic and sensory nerves from cranial ganglia and intrinsic innervation of the cerebral microcirculation[7] can be affected by tDCS. We now know that the nerve fibers from the ganglia belonging to the sympathetic, parasympathetic, and sensory nervous systems innervate the intracranial blood vessels[7]. Then, intracerebral blood vessels[7] are surrounded by astrocytes[8] that can communicate tDCS effects[9] on the pyramidal neurons[4] (“neurogenic hubs” of the NVU) to the blood vessels where this neurovascular coupling can be elucidated with fNIRS combined with EEG[10]. So, the “neurogenic hypothesis" can be applied to the transmural electrical stimulation of the perivascular nerves^27^ (e.g., neuropeptide Y is an important vasoconstrictor [11] of* [*sympathetic innervation*](https://www.sciencedirect.com/topics/medicine-and-dentistry/sympathetic-innervation)*, parasympathetic innervation for vasodilation), and nNOS expressing interneuron (e.g. NO for vasodilation [12]) in the intracranial blood vessels [7] viz. pial arteries and arterioles^26^ that can have a complex compounding effect to the blood volume (deoxy-Hb+oxy-Hb) and CVR including initial dip in the blood volume due to vasoconstriction[11]. Here, initial dip in the blood volume captured with fNIRS total haemoglobin concentration (tHb) based CVR to tDCS was found in some healthy subjects in prior work^48^. Here, tDCS effects on the blood vessels is postulated to be via transmural stimulation of perivascular neurotransmitters that can also promote noradrenaline release. Also, tDCS may affect the sensory fibers involved in cranial pain syndromes that stores calcitonin gene-related peptide (CGRP), substance P, neurokinin A, NOS, and nociceptin, inter alia [7]. Somatic afferents[13], [14] including extrinsic perivascular innervation [15] may also be responsible since tingling sensation (70.6%) and light itching sensation under the stimulus electrode (30.4%) are quite common adverse events that were observed even with short-duration sham tDCS [2], viz. trigeminal nerve stimulation with supraorbital tDCS electrode possibly due to the electrode edge-effects [16].”*

*However, besides direct effects on the neurons, tDCS can directly affect other constituents of the neurovascular unit including astrocyte and cerebral blood vessels, composed of pericytes, smooth muscle cells and endothelial cells, so, we perform hypothesis testing using grey-box model to answer “****To which compartment would this short duration tDCS be linked****”. We have clarified this in the Introduction section:*

*“**The signaling pathways modulating vascular response to the electric field in the perivascular space, astrocytes, and vascular SMCs were also evaluated in addition to the neuronal pathway [3] and compared using grey-box linear systems' transfer function analysis in eleven healthy subjects from our prior experimental studies^46,48^. In our previous works, Muthalib et al.^48^ found greater increase in oxy-Hb "within" than "outside" the spatial extent of the 4 × 1 high-definition (HD)-tDCS electrode. In the current study, we used that experimental data^46,48^ in conjunction with our novel grey-box linear model to investigate tHb as the hemodynamic correlate of the current density effects “within" the spatial extent (called the “targeted-region”) as well as "outside" the spatial extent (called the “nontargeted-region”) of the 4 × 1 HD-tDCS electrode. At both the targeted-region and the nontargeted-region, the increase in the blood volume during HD-tDCS was considered to be caused by a mix of metabolic and neurogenic factors that were mathematically formulated for grey-box linear systems identification. Here, grey-box linear systems identification was performed following model linearization of a physiologically detailed NVU model to find appropriate model fit (mean square error) and model complexity (Akaike information criterion[17]) to the tDCS-evoked change in the fNIRS-tHb data.”*

*So, in our study, we have considered a lumped model of neurovascular unit having 4 compartments: vascular smooth muscle, perivascular space, synaptic space, and astrocyte glial cell; and captured tDCS effects via each compartment on the blood vessel circumference to perform hypothesis testing using grey-box model to answer “****To which compartment would this short duration tDCS be linked****”.*

**2- The model selection is based on the fitting error which need to be well justified in the manuscript, especially considering the minimization of fitting error will lead to overfitting. How do authors consider overfitting?**

***Authors.*** *Thanks for your concerns. Indeed, overfitting is a concern and we addressed that by finding a model with the appropriate complexity (Akaike information criterion) for our fNIRS data set as listed in Table 3 in our manuscript.*

Table 3. Minimal realization transfer functions were obtained through Model Reducer (MATLAB, MathWorks, Inc., USA) for four model pathways from the detailed physiological model for the neurovascular unit compartment model.

| **Pathway transfer functions (TF)** | **Transfer functions from the compartmental neurovascular coupling model that were used for the initial parameterization of the four pathways** | **Parameterization** |
| --- | --- | --- |
| **TF1.**  **Synaptic Potassium → vessel circumference** | $TF1(s)= \frac{1}{(s+0.4)}TF2(s)$ | 11 poles, 3 zeros |
| **TF2.**  **Astrocytic membrane potential → vessel circumference** | $TF2\left( s \right)=\frac{\left( s+46.5 \right)}{\left( s+1.966 \right)\left( s+15.08 \right)} TF3(s)$ | 10 poles, 3 zeros |
| **TF3.**  **Perivascular Potassium → vessel circumference** | $TF3(s)=\frac{\left( s+2.371e07 \right)}{\left( s+2.974e04 \right)\left( s+1 \right)} TF4(s)$ | 8 poles, 2 zeros |
| **TF4.**  **Voltage gated ion channel on SMC → vessel circumference** | $TF4\left( s \right)=\frac{(s+2.962)}{(s+9.594e06)(s+20.69)(s+3.3)(s+0.2446)(s2 + 9.804s + 95.24)}$ | 6 poles, 1 zero |

*The lowest order TF4 had 6 poles and 1 zero and the highest order TF1 has 11 poles and 3 zeros. Here, fNIRS is a slow signal (<0.1 Hz) and so complex physiologically realistic model may lead to overfitting. More complex models may be fitted to invasive recordings for example in an animal model. So, our model selection addressed the model overfitting issue through: model linearization and model reduction (minimal realization transfer functions) from detailed complex physiological model (see Text S1), duration of fits (limited to the short duration tDCS when non-linear neuroplastic effects not expected), and residual checks (autocorrelation curves of the residuals and the cross-correlation curves between input and the residuals). For the grey-box analysis of the fNIRS tHb (normalized) as output to the input tDCS current waveform (normalized), model linearization of the four pathways from the physiologically detailed model was performed using Simulink's linear analysis tool (MathWorks, Inc., USA). This tool allows the linearization of complex nonlinear models at different operating points. The grey-box linear model with identifiable free parameters) was updated using the "Refine Existing Model'' approach in the System Identification Toolbox (MathWorks, Inc., USA) that uses prediction-error minimization algorithm (‘pem’ in MATLAB) to update the parameters of the initial model to improve the fit to the estimation data, thereby finding a balance between bias and variance. The model evaluation was based on the properties of the prediction-error cost function for each pathway (i.e., our 4 hypotheses) and the residuals for each participant, i.e., pathway-specific and participant-specific mean squared error (MSE) and Akaike information criterion (AIC). Then, residuals checks were performed based on the autocorrelation curves of the residuals and the cross-correlation curves between input and the residuals that were better for 150sec fits compared to 10-min fits – see Figure S3. These residual checks are important for validating model performance based on the whiteness test and the independence test.*

*We have now clarified these at the very beginning in the Introduction section:*

*“Here, grey-box linear systems identification was performed following model linearization of a physiologically detailed NVU model to find appropriate model fit (mean square error) and model complexity (Akaike information criterion[17]) to the tDCS-evoked change in the fNIRS-tHb data.”*

**Other comments**

**Abstract:**

**Context:**

1--

We found that the grey-box model pathway from perivascular potassium to the vessel circumference (Pathway 3) presented the best fit to fNIRS tHb time series with the least mean square error (MSE, median < 2.5%). Then, minimal realization transfer function with reduced-order approximations of the grey-box model pathways was fitted to the average tHb time series. Pathway 3, with nine poles and two zeros (all free parameters), provided the best Chi-Square Goodness of Fit of 0.0078.

2--

Therefore, our study provided a sound systems biology approach to investigate the hemodynamic response to tDCS that needs further investigation in health, aging, and disease. Future studies can leverage transcranial alternating current stimulation for frequency-dependent physiological entrainment of relevant pathways, e.g., oscillations driven by nonlinear calcium dynamics, that can provide additional insights based on our grey-box modeling approach.

-----------------------------------------------

**Comment 1: The logic from the two paragraphs (1 and 2) can be applied to any pathway, meaning if we replace Pathway 3 by Pathway N, the contribution of the study claimed by the red sentence will always work. Please describe the contribution of finding on Pathway 3 explicitly. In other words, how do authors justify that it is indeed by finding Pathway 3 which best fit the tHb so that leading the conclusion about their model is a sound systems biology approach to investigate the hemodynamic responses to tDCS?**

***Authors.*** *Thank you for your concerns. In our system biology approach, “model fitting” is used for hypothesis testing of the four pathways on whether the model is parsimonious (less complex model better) and conform to the data (goodness of fit). Under-fitting is known to induce bias while over-fitting induces variability so we used Akaike information criterion (AIC) to find balance (lowest AIC selected) – a concept from systems biology. Here, models are nested, i.e., some coordinates of more complex model are fixed in the less complex model, as shown in the Table 3 from manuscript. We have now clarified these at the very beginning in the Abstract:*

*“Here, grey-box linear systems identification was performed following model linearization of a physiologically detailed NVU model to find appropriate model fit (mean square error) and model complexity (Akaike information criterion[17]) to the tDCS-evoked change in the fNIRS-tHb data.”*

**Introduction**

**Comment 2: Authors said “Data-driven black-box systems approaches provide a correlate of neural and hemodynamic response at an abstract level under the assumption of neurovascular coupling at the cellular level; however, such black-box systems approaches do not aim to explicitly capture the underlying cellular mechanisms of action.”**

**I agree with the claim and love the idea of using an explicit generative model. However, using a more complex model(s) with biological meaningful parameters and relationships is not free. Sophisticated model requires more sophisticated evaluation methods, not just on how well the simulated data fit with real data. As stated by authors, “a deeper understanding of the signaling pathways inside the NVU is essential for a mechanistic understanding”, this means the goal is to infer the understanding of the pathway, not to select which pathway provides the best fit. There is no best model, and each model is assumed and justified based on our current knowledge. Therefore, if the goal is to achieve a mechanistic understanding, we may not want to select the best model, but assume these models are all reasonable and then compare the models’ behaviour on data fitting to understand the research question from the comparison itself instead of selecting the “best model”. We can step back a bit by saying, let us believe pathway 3 is the best, then how do authors justify the parameters value and, more importantly, to understand the contribution of each parameter to the final data fitting?**

***Authors.*** *Thank you for your concerns. Yes, we agree that the goal is hypothesis testing via model selection using systems biology approach so we removed “best fit” text and clarified our approach better which is to determine the reduced dimension model for our data set by finding the lowest Akaike information criterion (AIC). So, in this study, “model fitting” is used for hypothesis testing of the four pathways on whether the model is parsimonious (less complex model better) and conform to the data (goodness of fit). Under-fitting is known to induce bias while over-fitting induces variability so we used AIC to find a balance (lowest AIC selected) – a concept from systems biology. Here, models are nested, i.e., some coordinates of more complex models are fixed in the less complex model, as shown in the Table 3 in the manuscript. Also, the goodness of fit was based on the properties of the prediction-error cost function for each pathway (i.e., our hypotheses) and the residuals for each participant, i.e., pathway-specific and participant-specific mean squared error (MSE).*

*We have now clarified these points in the Introduction section:*

*“Here, grey-box linear systems identification was performed following model linearization of a physiologically detailed NVU model to find appropriate model fit (mean square error) and model complexity (Akaike information criterion[17]) to the tDCS-evoked change in the fNIRS-tHb data.”*

**The authors conducted excellent works on reviewing the models that are available from literature and justified the parameters selection and provided the final values in Supplementary. However, if the parameter values can be fixed from the literature review, then it means we have already understood enough this these pathways, so then how do we justify the motivation of this study? In other words, how do authors justify the potential bias on parameter value selection on the final fitting quantification?**

***Authors.*** *Thank you for your concerns. We would like to clarify that not all parameter values were fixed but we had to greatly reduce the complexity of the detailed physiological model that are complex non-linear models with many parameters whereas our data is fNIRS with slow signal changes. Therefore, in this study, we have used a reduced order grey-box linear model (as shown in the Table 3 in the manuscript) that were constrained by the physiology of the respective* *four tDCS perturbation pathways and compartments in the physiologically detailed NVU model. The grey-box linear model (with identifiable free parameters) was updated using the "Refine Existing Model" approach in the System Identification Toolbox (MathWorks, Inc., USA) that uses the prediction-error minimization (PEM) algorithm (‘pem’ in MATLAB) to update the parameters of the initial complex model to improve the fit to the estimation data. PEM technique considers the accuracy of the predictions computed for the observations and most tightly connected to systems theory as it explicitly exploits the dynamical structure of the studied system. We have clarified this in the section:* ***(F) Participant-specific Grey-box Linear Model Dynamics***

*“PEM technique considered the accuracy of the predictions computed for the observations and most tightly connected to systems theory as it explicitly exploits the dynamical structure of the studied system [23]. So, the subject-specific model evaluation in PEM was based on the properties of the prediction-error cost function for each pathway (i.e., hypothesis) for each subject constrained by the physiology of the respective tDCS perturbation pathways (see Text S1 and Table S1). We computed subject-specific MSE and AIC for each pathway (i.e., hypothesis) for grey-box linear model fitting.”*

**Comment 3: The authors talked about the initial dip in the last paragraph of the introduction. However, what is the relationship to this study?**

***Authors.*** *Thanks. We would like to clarify that initial dip in the oxy-hemoglobin concentration is well-known, for example, Muthalib et al. [6] found the group averaged initial dip in oxy-Hb within the first 15s of high-definition tDCS. However, initial dip in both the oxy-hemoglobin concentration and deoxy-hemoglobin concentration over a longer duration of 0-150s in few of our subjects (see Figure 11) indicated vasoconstriction (that we postulate is related to* *transmural electrical stimulation of the perivascular nerves during tDCS). We have now clarified this in the Introduction section:*

*“So, the “neurogenic hypothesis" can be applied to the transmural electrical stimulation of the perivascular nerves^27^ (e.g., neuropeptide Y is an important vasoconstrictor [11] of* [*sympathetic innervation*](https://www.sciencedirect.com/topics/medicine-and-dentistry/sympathetic-innervation)*, parasympathetic innervation for vasodilation), and nNOS expressing interneuron (e.g. NO for vasodilation [12]) in the intracranial blood vessels [7] viz. pial arteries and arterioles^26^ that can have a complex compounding effect to the blood volume (deoxy-Hb+oxy-Hb) and CVR including initial dip in the blood volume due to vasoconstriction[11]. Here, initial dip in the blood volume captured with fNIRS total haemoglobin concentration (tHb) based CVR to tDCS was found in some healthy subjects in prior work^48^. Here, tDCS effects on the blood vessels is postulated to be via transmural stimulation of perivascular neurotransmitters that can also promote noradrenaline release.”*

**Throughout the manuscript, the duration of interest for fNIRS signal was 0-150s and the time scale of the plot was 0 – 10 minutes, how can we see the dips?**

***Authors.*** *Apologies for the confusion. We analyzed the response of the physiologically detailed neurovascular coupling (NVC) model for the whole 10 minutes (see Table S4). Then, we simplified the physiologically detailed complex model using prediction-error minimization (PEM) algorithm (‘pem’ in MATLAB) to update the parameters to improve the fit fNIRS data set with slow signal changes. The grey-box linear model was fitted to the initial 120sec + 30sec ramp-up time = 150sec since 3 minutes following the start of tDCS the aftereffects starts where neuroplastic changes are induced [Nitsche, M. A. & Paulus, W. Excitability changes induced in the human motor cortex by weak transcranial direct current stimulation. The Journal of physiology 527, 633-639 (2000).] Since we did not aim to model the nonlinear neuroplastic changes with our grey-box linear model, where neuroplastic changes are possible due to calcium accumulation that we did not capture in grey-box linear model, so we restricted model fitting to less than 150sec (Figure 9). Moreover, residuals checks were performed based on the autocorrelation curves of the residuals and the cross-correlation curves between input and the residuals that were better for 150 sec fits – see Figure S3. This is clarified in the manuscript in section* ***(B) Physiologically detailed model linearization for grey-box analysis using subject-specific fNIRS-tHb data****:*

*“**We limited the fNIRS-tHb data within the lower frequency band (0.01–0.05Hz) to capture the initial transient response (not steady-state vessel oscillations)^28^ during first 150sec (30sec ramp-up+120sec steady-state) of tDCS perturbation.”*

*Also, we have clarified (and highlighted) the initial dip in Figure 11 (see below) and section* ***(C) Reduced dimension grey-box linear model analysis of averaged fNIRS-tHb data:***

*“**The average tHb time series during initial transient 150 sec of HD-tDCS from eight subjects with anti-correlated oxy-Hb & deoxy-Hb for 600 sec (i.e., correlation coefficient <-0.5 between oxy-Hb & deoxy-Hb at targeted-region – see Table S2) was used for model fitting of the minimal realization transfer function. Figure 11 shows the subjects P3, P4, and P10 who were rejected due to poor anti-correlated oxy-Hb & deoxy-Hb. Figure 11 also shows an initial dip (0-150sec) in the tHb in these subjects that is also present in oxy-Hb & deoxy-Hb timeseries.”*

Figure 11. Oxy-hemoglobin (Oxy-Hb), deoxy-hemoglobin (Dxy-Hb), and total-hemoglobin (tHb) time series at the targeted-region during anodal HD-tDCS for the subjects P3, P4, P10 who were rejected due to poor anti-correlated Oxy-Hb & Dxy-Hb measures of -0.0692, -0.1900, and -0.3768 respectively. These subjects also demonstrated an initial dip in the tHb (also, Oxy-Hb & Dxy-Hb) time series that is highlighted with a black ellipse in the Oxy-Hb & Dxy-Hb time series.

**Results**

**Comment 4: Authors claimed that “In most of the participants, the time course of changes in tHb in the region within the boundary of the HD-tDCS montage showed a steep initial increase compared to the contralateral non-stimulated sensorimotor area that showed a decrease in some participants, which may be related to inter-hemispheric inhibition.”**

**1) We understand the variability of fNIRS data, but “most of the participants” is not clear. Please state the portion, even if it is based on visual inspection.**

***Authors.*** *Apologies for the confusion. We have now removed that statement and presented Figure 11 that shows subjects who have an initial dip (also, they did not present a steep increase within 0-150sec). We have also plotted the tHb from the targeted-region and the nontargeted-region together in Figure 3 so that the difference is visible. The average tHb time series at the targeted-region and the nontargeted-region together also showed the difference across all subjects. Figure 3 presented below:*

(A)

(B)

(C)

**Figure 3.** (A) Normalized time course of changes in the total hemoglobin concentration during anodal HD-tDCS (2mA current for 600 seconds, with ramp-up period of 30 seconds) at the targeted-region (red) and the nontargeted-region (blue) of eleven healthy subjects (P1-Hbt to P11-Hbt). (B) Ensemble averaged HD-tDCS evoked changes in total hemoglobin concentration (mean±standard deviation) at the targeted-region. (C) Ensemble averaged HD-tDCS evoked changes in total hemoglobin concentration (mean±standard deviation) at the nontargeted-region.

**2) How do authors justify it is within the boundary? From Figure 6 and the method section, the boundary channels are not even included in the data analysis. (please see the detailed comments in the method section).**

***Authors.*** *Thank you for your concerns. The data in this study was from prior work [Muthalib M, Besson P, Rothwell J, Perrey S. Focal Hemodynamic Responses in the Stimulated Hemisphere During High-Definition Transcranial Direct Current Stimulation. Neuromodulation. 2018 Jun;21(4):348-354. doi: 10.1111/ner.12632. Epub 2017 Jul 17. PMID: 28714545.] where focal responses were shown. We have clarified this in the manuscript:*

*“**Muthalib et al^48^ showed that anodal HD-tDCS induced increases in oxy-Hb during 10min of stimulation (at 2mA) in the sensorimotor cortex region within the vicinity of the 4 x 1 HD-tDCS montage compared to the region outside this boundary. At the same time, there were minimal oxy-Hb changes in the contralateral non-targeted sensorimotor cortex region.”*

*In this study on grey-box model evaluation, we considered the local effect of HD-tDCS, i.e. the targeted-region as well as the contralateral nontargeted-region. Figure 6 has been updated and it now depicts the fNIRS channels used for the analysis, as shown below.*

(A)

(B)(C)

Figure 1. (A) HD-tDCS-fNIRS montage on a 10/10 EEG system. The fNIRS time series data from the transmitter-receiver optode combinations in the vicinity of 4x1 HD-tDCS electrodes (fNIRS channels encircled in red, left hemisphere: C5-CP3, C1-CP3, C5-FC3, and C1-FC3), i.e. the targeted-region, as well as the non-targeted region in the contralateral hemisphere (fNIRS channels encircled in blue, right hemisphere: C2-FC4, C2-CP4, C6-FC4, and C6-CP4) were used for the grey-box modeling. (B) Measurement sensitivity of the fNIRS channels at the grey matter on the left hemisphere along with the HD-tDCS electrodes in the Atlas Viewer open-source package (<https://github.com/BUNPC/AtlasViewer>). The sensitivity values are displayed logarithmically by the color scale. (C) Measurement sensitivity of the fNIRS channels at the grey matter on the right hemisphere in the Atlas Viewer open source package (<https://github.com/BUNPC/AtlasViewer>). The sensitivity values are displayed logarithmically by the color scale.

**3) Most importantly, in Figure 1,**

**a) each tHb time course started from a different value. Did authors perform baseline correction? If not, how to justify the statement of decrease and increase? Since fNIRS is a relative measurement, please clarify what the reference for these changes is. This is also seen in Supplement D, both HbO and HbR started from different values, and no baseline is shown.**

***Authors.*** *Yes, baseline correction was performed using the average of resting data before the tDCS perturbation. We have now clarified this in* **(B) fNIRS-tHb response during HD-tDCS(experimental data):**

*“Figure 3 shows the change in the normalized tHb from baseline (mean of 2 min before tDCS perturbation) obtained during anodal HD-tDCS stimulation from fNIRS channels around the 4x1 HD-tDCS anode, i.e., the targeted-region and from the nontargeted-region at the contralateral sensorimotor cortex (shown by the montage in Figure 1). ”*

*However, since the average of pre-tDCS baseline data did not match the first time point of the post-tDCS data so HbO and HbR started from different values.*

**b) since the time course is averaged among subjects, what is the variance? Could the authors please show the error bar?**

***Authors.*** *Apologies for the oversight. We have now rectified the Figure 3, as shown below.*

(B)

(C)

**Figure 3.** (B) Ensemble averaged HD-tDCS evoked changes in total hemoglobin concentration (mean±standard deviation) at the targeted-region. (C) Ensemble averaged HD-tDCS evoked changes in total hemoglobin concentration (mean±standard deviation) at the nontargeted-region.

**c) it is nice to compare with the non-stimulated region, however, why not plot them in the same figure for better visual comparison?**

***Authors.*** *Thanks for your advice. We have now plotted them together in Figure 3A, as shown below.*

(A)

**Figure 3.** (A) Normalized time course of changes in the total hemoglobin concentration during anodal HD-tDCS (2mA current for 600 seconds, with ramp-up period of 30 seconds) at the targeted-region (red) and the nontargeted-region (blue) of eleven healthy subjects (P1-Hbt to P11-Hbt).

**4) could the authors indicate in each figure where is the increase and decrease or simply mention the duration of them in the caption? Because the signal just fluctuates and there is an increase/decrease everywhere.**

***Authors.*** *Apologies for the confusion. We have clarified that the increase and decrease in the first 150sec can be observed in the averaged tHb plots in Figure 3(B) and 3(C) respectively in section* **(B) fNIRS-tHb response during HD-tDCS (experimental data):**

*“The 0-150sec time course of changes in tHb at the targeted-region showed a steeper increase compared to the nontargeted-region that is more evident in Figure 3B and 3C that show ensemble averaged time courses of tHb changes across subjects at the targeted-region and at the nontargeted-region respectively.”*

**Comment 5: I might be wrong for this point, but the authors stated, “Figure 2(B) shows that physiologically detailed neurovascular coupling pathways generated low-frequency oscillations in the frequency range of 0.05 – 0.2 Hz, driven by nonlinear calcium dynamics in various compartments, during the time course of the vessel response”**

**Looking at this figure, I counted 3, 16, 5, 7 periodic components for pathways 1 to 4, respectively. Within 10 minutes, the corresponding frequency is 0.005, 0.026, 0.008 and 0.012Hz, only pathway 2 and 4 is within filter band 0.05 – 0.2Hz or the band of interest 0.01-0.05Hz explained later in method. Could the authors please clarify this?**

***Authors.*** *Apologies for the confusion, we indicated physiologically detailed non-linear Witthoft and Karniadakis, 2012 model (Figure 4 below) and not the grey-box linear model.*

Figure 4. Normalized vessel radius changed from the neurovascular unit models during neuronal stimulation (30 to 70 sec) shown by the shaded region. The period from 0 to 30 sec shows numerical transients where the model (c) settles to a non-zero vessel radius from zero initial condition. Model (c) also captured the after-effect of the neuronal stimulation on the normalized vessel radius. Three relevant physiological models’ simulation from Table 3: Model (a) Kenny et al., 2018, Model (b) Mathias et al., 2018, and Model (c) Witthoft and Karniadakis, 2012.

*We have now added the simulation of the physiologically detailed non-linear model in Figure 8B as shown below.*

(B)

Figure 8. (B). Pathway 1: tDCS current density perturbing vessel circumference through synaptic potassium pathway, Pathway 2: tDCS current density perturbing vessel circumference through the astrocytic pathway, Pathway 3: tDCS current density perturbing vessel circumference through perivascular potassium pathway, and Pathway 4: tDCS current density perturbing vessel circumference through smooth muscle cell voltage-gated ion current channels pathway. Shown in (B), all the tDCS perturbation pathways has an initial transient response and then generated steady-state vessel oscillations.

**If these fluctuations are not the frequency of interest, then what is the difference between 4 pathways after ignoring these components?**

***Authors.*** *In this study, we were not interested in the steady-state vessel oscillation but the initial transient response i.e. CVR to tDCS within 0-150sec of HD-tDCS application. We have now clarified this as well as the difference in the four tDCS perturbation pathways as shown in Figure 6. The following text is from the section:* **(D) Physiologically detailed Neurovascular Compartmental Dynamics**

*“Our goal was mechanistic grey-box modeling for hypothesis testing, where the hypotheses are formulated as a set of mathematical equations for data fitting to identify core predictions from biological criteria^81^. We identified four nested NVU compartmental pathways (see Figure 6) where tDCS perturbed a state variable at each of the four NVU compartments (see Table 2 for details on compartmental state variables) for hypothesis testing of the initial (0-150sec) tDCS effects on the NVU leading to blood vessel (circumference) volume changes. Figure 6 shows the nested model for each of the four pathways starting from tDCS scalp current density that perturbed synaptic potassium (K+) released from active neurons (∆*$J_{K_{s}}$*) for Pathway 1, astrocytic transmembrane current (∆*$I_{T}$*) for Pathway 2, perivascular potassium (K+) concentration (*${\Delta\left[ K^{+} \right]}_{T}$*) for Pathway 3, and voltage-gated ion channel current (∆*$I_{KV}$*) on the smooth muscle cell (SMC) for Pathway 4.”*

**Comment 6: In Figure 1, all time courses in As started from 0 value, but all in Bs started from 0.4. Could the authors please explain why?**

**In the caption, “The average fNIRS-tHb response across all participants is also shown with a dashed line.” Is this a dashed line representing the real data? Or it is averaged from all color coded curves from simulation?**

***Authors.*** *Apologies for the confusion. To determine fNIRS-tHb response, the mean value from the baseline (before HD-tDCS) was subtracted and then the time course was normalized with the maximum (the maximum value gets transformed into a 1) for the changes in total hemoglobin concentration changes (fNIRS-tHb response). So, all time courses are normalized that we have now clarified in the section:* ***(B) fNIRS-tHb response during HD-tDCS(experimental data):***

*“Figure 3 shows the change in the normalized tHb from baseline (mean of 2 min before tDCS perturbation) obtained during anodal HD-tDCS stimulation from fNIRS channels around the 4x1 HD-tDCS anode, i.e., the targeted-region and from the nontargeted-region at the contralateral sensorimotor cortex (shown by the montage in Figure 1). The 0-150sec time course of changes in tHb at the targeted-region showed a steeper increase compared to the nontargeted-region that is more evident in the ensemble averaged time courses of tHb (Figure 3B and 3C) at the targeted-region and at the nontargeted-region respectively across subjects.”*

*Since the average of pre-tDCS baseline data did not match the first time point of the post-tDCS data so HbO and HbR started from different values for the targeted-region and the nontargeted region. The averaged data is now shown separately in Figure 3B and 3C as shown below.*

(B)(C)

**Figure 3.** (B) Ensemble averaged HD-tDCS evoked changes in total hemoglobin concentration (mean±standard deviation) at the targeted-region. (C) Ensemble averaged HD-tDCS evoked changes in total hemoglobin concentration (mean±standard deviation) at the nontargeted-region.

*Then, in Figure 9, the initial condition for the grey-box linear model were also fitted to the fNIRS-tHb data so they followed the ensemble averaged values shown in Figure 3(B) and 3(C), i.e., close to zero for the targeted-region and 0.4 for the nontargeted-region. This initial condition of grey-box linear model fits for 0-150sec is evident from the ensemble average fNIRS-tHb response data across all subjects as shown with a dashed line (they are same as the mean time series in Figure 3B and 3Cbut for 0-150sec). We have now clarified this in the section* ***(B) Physiologically detailed model linearization for grey-box analysis using subject-specific fNIRS-tHb data:***

*“Figure 9 also shows the ensemble averaged fNIRS-tHb response data for 0-150sec across all subjects with a dashed line (from Figure 3B and 3C).”*

**It was a bit confusing since in the legend, dashed line just followed each subject’s line, it led me to think dashed line is just averaged from all individuals.**

***Authors.*** *Apologies for the confusion. Yes, you are right. In Figure 9, the initial condition for the grey-box linear model fits were also fitted to the fNIRS-tHb data so they followed the ensemble averaged values shown in Figure 3(B) and 3(C), i.e., close to zero for the targeted-region and 0.4 for the nontargeted-region. This initial condition of grey-box linear model fits for 0-150sec is evident from the ensemble average fNIRS-tHb response data across all subjects as shown with a dashed line (they are same as the mean time series in Figure 3B and 3C but for 0-150sec). We have now clarified this in the section* ***(B) Physiologically detailed model linearization for grey-box analysis using subject-specific fNIRS-tHb data:***

*“Figure 9 also shows the ensemble averaged fNIRS-tHb response data for 0-150sec across all subjects with a dashed line (from Figure 3B and 3C).”*

**Comment 7: MSE is shown in Table1A and Table1B for stimulation and non- stimulation side.**

**1) please report the error of the mean (i.e. sd) even if one can find it from figure 4.**

***Authors.*** *Thanks for your advice. We have now reported the median, mean, and the standard deviation from the boxplots – shown below (see Table S5).*

| **Targeted-region** | **Pathway 1** | **Pathway 2** | **Pathway 3** | **Pathway 4** |
| --- | --- | --- | --- | --- |
| **P1** | 0.07055 | 0.2295 | 0.0422 | 0.09602 |
| **P2** | 0.02375 | 0.05435 | 0.001509 | 0.03862 |
| **P3** | 0.02323 | 0.05801 | 0.02595 | 0.05892 |
| **P4** | 0.01579 | 0.08174 | 0.05264 | 0.01266 |
| **P5** | 0.006357 | 0.01926 | 0.00382 | 0.02457 |
| **P6** | 0.02273 | 0.03176 | 0.01555 | 0.01968 |
| **P7** | 0.06432 | 0.1134 | 0.06047 | 0.1084 |
| **P8** | 0.02715 | 0.02585 | 0.03409 | 0.03307 |
| **P9** | 0.02488 | 0.00156 | 0.004376 | 0.001343 |
| **P10** | 0.009925 | 0.05779 | 0.01008 | 0.02365 |
| **P11** | 0.03855 | 0.04509 | 0.01875 | 0.05301 |
| **MEAN** | 0.029748 | 0.065301 | 0.024494 | 0.042722 |
| **STDEV** | 0.020568 | 0.062488 | 0.020434 | 0.033871 |
| **MEDIAN** | 0.02375 | 0.05435 | 0.01875 | 0.03307 |
| **Nontargeted-region** | **Pathway 1** | **Pathway 2** | **Pathway 3** | **Pathway 4** |
| **P1** | 0.1229 | 0.2741 | 0.162 | 0.1898 |
| **P2** | 0.04355 | 0.06401 | 0.07775 | 0.01848 |
| **P3** | 0.0901 | 0.347 | 0.08637 | 0.09435 |
| **P4** | 0.04326 | 0.217 | 0.2944 | 0.1237 |
| **P5** | 0.0245 | 0.1864 | 0.2464 | 0.02318 |
| **P6** | 0.0593 | 0.02059 | 0.1225 | 0.01465 |
| **P7** | 0.07142 | 0.0689 | 0.195 | 0.0683 |
| **P8** | 0.02989 | 0.05707 | 0.02207 | 0.03159 |
| **P9** | 0.04824 | 0.09063 | 0.004094 | 0.01612 |
| **P10** | 0.01748 | 0.01321 | 0.01051 | 0.02899 |
| **P11** | 0.02444 | 0.02422 | 0.07726 | 0.03184 |
| **MEAN** | 0.05228 | 0.123921 | 0.118032 | 0.058273 |
| **STDEV** | 0.032041 | 0.113997 | 0.096696 | 0.056343 |
| **MEDIAN** | 0.04355 | 0.0689 | 0.08637 | 0.03159 |

*We have also presented the results in the section* ***(B) Physiologically detailed model linearization for grey-box analysis using subject-specific fNIRS-tHb data:***

*“Then, Figure 10 shows the boxplot of the mean square error (MSE) and the Akaike information criterion (AIC) across 11 subjects from subject-specific grey-box analysis after physiologically detailed model linearization. Notches display the variability of the median between samples. The width of a notch is computed so that the boxes whose notches do not overlap have different medians at the 5% significance level. Here, for MSE of the four pathways for the targeted-region, the mean are 0.029, 0.065, 0.024, 0.042, the standard deviation are 0.020, 0.062, 0.020, 0.033, and the median are 0.023, 0.054, 0.018, 0.033 respectively. Then, the MSE of the four pathways for the nontargeted-region, the means are 0.052, 0.123, 0.118, 0.058, the standard deviation are 0.032, 0.113, 0.096, 0.056, and the median are 0.043, 0.068, 0.086, 0.031. Then, for AIC of the four pathways for the targeted-region, the means are -0.459, -0.216, -1.743, -1.748, the standard deviation are 0.862, 1.496, 1.433, 1.173, and the median are -0.606, -0.162, -1.726, -1.659. Then, for AIC of the four pathways for the nontargeted-region, the means are 1.386, 1.313, 0.478, -0.747, the standard deviation are 0.599, 1.264, 1.346, 0.966, and the median are 1.366, 1.261, 0.814, -0.915. Figure 10A shows that the tDCS perturbation Pathway 3, from the perivascular K+ to vessel circumference, presented the least MSE (median <2.5%) across all subjects at the targeted-region, followed by the Pathway 1. Figure 10B shows that the tDCS perturbation Pathway 4 gave lowest median MSE and medial AIC across all subjects for the grey-box linear model fits at the contralateral nontargeted-region. Here, over-fitting induces variability while under-fitting is known to induce bias so we used Akaike information criterion (AIC) to find a balance (lowest AIC selected). Figure 10C shows that the tDCS perturbation Pathway 3 presented the lowest AIC (median -1.726) across all subjects for the grey-box linear model fits at the targeted-region while Figure 10D shows that the tDCS perturbation Pathway 4 gave the lowest AIC (median -0.915) across all subjects at the contralateral nontargeted-region. Residuals checks were performed based on the autocorrelation curves of the residuals and the cross-correlation curves between input and the residuals – see Figure S3. Therefore, based on MSE and AIC, tDCS perturbation Pathway 3 was selected for the grey-box linear model fits at the targeted-region while Pathway 4 was selected for the contralateral nontargeted-region. For MSE comparison with long-term (0-600sec) tDCS effects, grey-box modeling of the complete 10 min of tHb data at the targeted-region was performed (see Figure S1) where the perturbation Pathway 3 again resulted in the lowest MSE (median <0.5%) – see Table S5 and Figure S2.”*

**2) The smallest MSE in the stimulation side is Pathway 3 which is the core result of this manuscript. Let us first ignore the justification of using MSE to select the model (which may lead to overfitting), the smallest MSE in non-stimulation case is Pathway 1, and the value is similar to Pathway 2 and 4 in simulated case. Then how do we infer the fact that Pathway1 is the best fit in non-stimulation case?**

***Authors.*** *Apologies for the confusion. In our systems biology approach, the core result include the complete analysis of the response of the physiologically detailed neurovascular coupling (NVC) model for various tDCS perturbation pathways. Here, we simplified the physiologically detailed complex model using prediction-error minimization (PEM) algorithm (‘pem’ in MATLAB) to update the parameters to improve the fit fNIRS data set with slow signal changes. Then, the grey-box linear model was fitted to the initial 120sec+ 30sec ramp-up time = 150sec since 3 minutes following the start of tDCS the aftereffects starts where neuroplastic changes are induced [Nitsche, M. A. & Paulus, W. Excitability changes induced in the human motor cortex by weak transcranial direct current stimulation. The Journal of physiology 527, 633-639 (2000).]*

*Here, our goal is hypothesis testing via model selection using systems biology approach, so, in this study, “model fitting” is used for hypothesis testing of the four pathways on whether the model is parsimonious (less complex model better) and conform to the data (goodness of fit). Under-fitting is known to induce bias while over-fitting induces variability so we used Akaike information criterion (AIC) to find balance (lowest AIC selected) – a concept from systems biology. Here, models are nested, i.e., some coordinates of more complex model are fixed in the less complex model, as shown in the Table 3 in manuscript. Here, Pathway 3 was selected that consistently performed on whether the model is parsimonious (less complex model better) and conform to the data (goodness of fit).We have now clarified these at the very beginning in the Introduction section:*

*“Here, grey-box linear systems identification was performed following model linearization of a physiologically detailed NVU model to find appropriate model fit (mean square error) and model complexity (Akaike information criterion[17]) to the tDCS-evoked change in the fNIRS-tHb data.”*

*The AIC values are presented below from the Table S6*

| **Targeted-region** | **Pathway 1** | **Pathway 2** | **Pathway 3** | **Pathway 4** |
| --- | --- | --- | --- | --- |
| **P1** | 0.3485 | 1.278 | -0.9154 | -0.5932 |
| **P2** | -0.7401 | -0.1623 | -4.2463 | -1.504 |
| **P3** | 0.7376 | 1.2779 | -0.2768 | -1.0815 |
| **P4** | 0.3514 | 1.6208 | 0.4307 | -1.7442 |
| **P5** | -2.0581 | -1.1999 | -3.3176 | -1.9562 |
| **P6** | -0.7842 | -0.6997 | -1.9137 | -2.1783 |
| **P7** | 0.256 | 0.573 | -0.5556 | -0.4724 |
| **P8** | -0.6062 | -0.9055 | -1.1288 | -1.659 |
| **P9** | -0.6938 | -3.7131 | -3.1817 | -4.8628 |
| **P10** | -1.6127 | -0.1009 | -2.3469 | -1.9945 |
| **P11** | -0.2558 | -0.349 | -1.7263 | -1.1872 |
| **MEAN** | -0.45976 | -0.21643 | -1.74349 | -1.74848 |
| **STDEV** | 0.862721 | 1.496699 | 1.433759 | 1.173804 |
| **MEDIAN** | -0.6062 | -0.1623 | -1.7263 | -1.659 |
| **Nontargeted-region** | **Pathway 1** | **Pathway 2** | **Pathway 3** | **Pathway 4** |
| **P1** | 2.4033 | 2.8306 | 1.5551 | 0.9633 |
| **P2** | 1.3661 | 0.0012 | -0.3042 | -2.2412 |
| **P3** | 2.0931 | 3.0666 | 0.9259 | 0.2643 |
| **P4** | 1.3595 | 2.5973 | 2.1521 | 0.5347 |
| **P5** | 0.7909 | 2.4453 | 1.9741 | -1.1395 |
| **P6** | 1.6804 | 0.242 | 1.2756 | -1.5986 |
| **P7** | 1.8609 | 0.075 | 0.6153 | -0.9339 |
| **P8** | 0.9899 | 1.2615 | -0.4386 | -0.83 |
| **P9** | 1.4685 | 1.724 | -2.1234 | -1.5029 |
| **P10** | 0.4532 | -0.2016 | -1.1802 | -0.9158 |
| **P11** | 0.7884 | 0.4045 | 0.8144 | -0.8219 |
| **MEAN** | 1.386745 | 1.313309 | 0.478736 | -0.74741 |
| **STDEV** | 0.599439 | 1.264959 | 1.346802 | 0.966945 |
| **MEDIAN** | 1.3661 | 1.2615 | 0.8144 | -0.9158 |

*Then, MSE and Chi-Square Goodness-of-Fit (that determines the quality of fit [Cedersund, G. & Roll, J. Systems biology: model based evaluation and comparison of potential explanations for given biological data. The FEBS journal 276, 903-922 (2009)] for such nested models) provided whether the model conform to the data (goodness of fit). Here, not just in terms of MSE but reduced dimension grey-box linear model for the Pathway 3 with 9 poles and 2 zeros (all free parameters) provided also the best Chi-Square Goodness-of-Fit of 0.0078.*

*We have clarified and discussed this further in the Results* section **(B) Physiologically detailed model linearization for grey-box analysis using subject-specific fNIRS-tHb data:**

*“Figure 10A shows that the tDCS perturbation Pathway 3, from the perivascular K+ to vessel circumference, presented the least MSE (median <2.5%) across all subjects at the targeted-region, followed by the Pathway 1. Figure 10B shows that the tDCS perturbation Pathway 4 gave lowest median MSE and medial AIC across all subjects for the grey-box linear model fits at the contralateral nontargeted-region. Here, over-fitting induces variability while under-fitting is known to induce bias so we used Akaike information criterion (AIC) to find a balance (lowest AIC selected). Figure 10C shows that the tDCS perturbation Pathway 3 presented the lowest AIC (median -1.726) across all subjects for the grey-box linear model fits at the targeted-region while Figure 10D shows that the tDCS perturbation Pathway 4 gave the lowest AIC (median -0.915) across all subjects at the contralateral nontargeted-region. Residuals checks were performed based on the autocorrelation curves of the residuals and the cross-correlation curves between input and the residuals – see Figure S3. Therefore, based on MSE and AIC, tDCS perturbation Pathway 3 was selected for the grey-box linear model fits at the targeted-region while Pathway 4 was selected for the contralateral nontargeted-region. For MSE comparison with long-term (0-600sec) tDCS effects, grey-box modeling of the complete 10 min of tHb data at the targeted-region was performed (see Figure S1) where the perturbation Pathway 3 again resulted in the lowest MSE (median <0.5%) – see Figure S2.”*

**Comment 8: Authors claimed that “The grey-box model simulated output of all the four pathways demonstrated an initial dip in the tHb concentration (related to the vessel circumference in the physiologically detailed model) which was based on the experimental data (see Supplementary materials, section D: Oxy and Deoxy-Hemoglobin Changes at the Stimulated Region).”**

**1) I could not find the initial dip in the tHb results in Supplementary D. Could authors please indicate them in the figure? I do see the dips in simulations.**

***Authors.*** *Apologies for the confusion. We have now included Figure 11 that shows initial dip in 0-150 sec of tHb time series in subjects P3, P4, P10.*

Figure 11. Oxy-hemoglobin (Oxy-Hb), deoxy-hemoglobin (Dxy-Hb), and total-hemoglobin (tHb) time series at the targeted-region during anodal HD-tDCS for the subjects P3, P4, P10 who were rejected due to poor anti-correlated Oxy-Hb & Dxy-Hb measures of -0.0692, -0.1900, and -0.3768 respectively. These subjects also demonstrated an initial dip in the tHb (also, Oxy-Hb & Dxy-Hb) time series that is highlighted with a black ellipse in the Oxy-Hb & Dxy-Hb time series.

**2) please show the error bar (Supplementary D) in the time course plot since it is averaged among channels.**

***Authors.*** *Thanks for the advice. We have included error bars in the ensemble averaged plots in Figure 3B and 3C, as shown below.*

(B)(C)

**Figure 3.** (B) Ensemble averaged HD-tDCS evoked changes in total hemoglobin concentration (mean±standard deviation) at the targeted-region. (C) Ensemble averaged HD-tDCS evoked changes in total hemoglobin concentration (mean±standard deviation) at the nontargeted-region.

**Comment 9: there is no statistics comparison of boxplot in figure 4. I am not insisting that authors have to show significant comparison but at least some level of statistics comparison are necessary.**

**1) Especially, pathway 1 has a bit high MSE but clearly a much smaller variance than pathway 3. Then can we choose pathway 1 instead of 3?**

***Authors.*** *Thanks for the advice. We have now shown in the boxplot the width of a notch that is computed so that boxes whose notches do not overlap have different medians at the 5% significance level, as clarified in section* ***(B) Physiologically detailed model linearization for grey-box analysis using subject-specific fNIRS-tHb data:***

*“Then, Figure 10 shows the boxplot of the mean square error (MSE) and the Akaike information criterion (AIC) across 11 subjects from subject-specific grey-box analysis after physiologically detailed model linearization. Notches display the variability of the median between samples. The width of a notch is computed so that the boxes whose notches do not overlap have different medians at the 5% significance level.”*

*(A)*

*(B)*

*(C)*

*(D)*

Figure 10. Boxplot across 11 subjects of the mean square error (MSE) and Akaike Information Criterion (AIC) of grey-box linear model fits for the four tDCS perturbation pathways. Pathway 1: tDCS perturbing vessel response through synaptic potassium pathway, Pathway 2: tDCS perturbing vessel response through the astrocytic pathway, Pathway 3: tDCS perturbing vessel response through perivascular potassium pathway, and Pathway 4: tDCS perturbing vessel response through the SMC pathway. On each box, the central mark indicates the median, and the bottom and top edges of the box indicate the 25th and 75th percentiles, respectively. If the notches in the box plot do not overlap, one can conclude, with 95% confidence that the true medians do differ. The whiskers extend to the most extreme data points not considered outliers, and the outliers are plotted individually using the red '+' symbol. The mean is also shown with a black '+' symbol. (A) MSE for targeted-region. (B) MSE for nontargeted-region. (C) AIC for targeted-region. (D) AIC for nontargeted-region.

*We have also clarified in the section* ***(C) Reduced dimension grey-box linear model analysis of averaged fNIRS-tHb data:*** *that this is a nested model analysis so Pathway 1 is most complex that incorporated other models (as shown in the Table 3) so can overfit if we only look at MSE. Here, not just in terms of MSE but reduced dimension grey-box linear model for the Pathway 3 with 9 poles and 2 zeros (all free parameters) provided also the best Chi-Square Goodness-of-Fit of 0.0078 ([Cedersund, G. & Roll, J. Systems biology: model based evaluation and comparison of potential explanations for given biological data. The FEBS journal 276, 903-922 (2009)] good for such nested models). We have clarified this further in the Section* **(*C) Reduced dimension grey-box linear model analysis of averaged fNIRS-tHb data*** *as given below:*

*“Fitting of the least complex (model order) Pathway 4 model, TF4, with 7 poles and 1 zero (all free parameters) provided an MSE of 0.0031 and Chi-Square Goodness of Fit of 0.0104. Then, the next complex (model order) Pathway 3 model, TF3, with 9 poles and 2 zeros (all free parameters) provided an MSE of 0.0025 and Chi-Square Goodness of Fit of 0.0078. Then, the next complex (model order) Pathway 2 model, TF2, with 11 poles and 3 zeros (all free parameters) provided an MSE of 0.0025 and Chi-Square Goodness of Fit of 0.0085. The most complex (model order) Pathway 1 model, TF1, with 12 poles and 3 zeros (all free parameters) provided an MSE of 0.0264 and Chi-Square Goodness of Fit of 0.0647. Here, more parameters in more complex (model order) models worsened the Chi-Square Goodness of Fit even with similar MSE, e.g., TF2 higher model order than TF3. Therefore, TF3 for the tDCS perturbation Pathway 3 provided a balance in terms of Chi-Square difference test for nested pathways^90^ for the HD-tDCS-induced initial transient tHb changes within 0.01 and 0.05 Hz.”*

**2) Moreover, in the non-stimulation side, pathway 1 also provided good MSE. If the authors prefer involving the non-stimulation side as a control and it might be fair to select pathway3 since it did not provide low MSE in the non-stimulation side as pathway1. However, how do we justify the use of the contralateral side as a control?**

***Authors.*** *Thanks for your concerns. Yes, we have removed the description of the contralateral side as control and compared the targeted-region and the contralateral nontargeted-region. We have clarified the selection of Pathway 4 for the nontargeted-region in the section* ***(B) Physiologically detailed model linearization for grey-box analysis using subject-specific fNIRS-tHb data*** *as shown below.*

*“Figure 10B shows that the tDCS perturbation Pathway 4 gave lowest median MSE and medial AIC across all subjects for the grey-box linear model fits at the contralateral nontargeted-region. Here, over-fitting induces variability while under-fitting is known to induce bias so we used Akaike information criterion (AIC) to find a balance (lowest AIC selected). Figure 10C shows that the tDCS perturbation Pathway 3 presented the lowest AIC (median -1.726) across all subjects for the grey-box linear model fits at the targeted-region while Figure 10D shows that the tDCS perturbation Pathway 4 gave the lowest AIC (median -0.915) across all subjects at the contralateral nontargeted-region”*

**3) We know M1 and S1 both have connections to the contralateral side M1 and S1. In early fNIRS/TMS studies, due to hardware difficulties of stimulating and detecting on the same side. People did stimulate M1 and apply fNIRS to probe the contralateral M1, and used the connectivity to rationalize the investigation [3]. In contrast, in this manuscript, the authors use the contralateral side as a control. Then how do we justify the eligibility of the contralateral side as a control? Maybe using other regions for control is better (see comments for method).**

**[3] Chiang TC, Vaithianathan T, Leung T, Lavidor M, Walsh V, Delpy DT. Elevated haemoglobin levels in the motor cortex following 1 Hz transcranial magnetic stimulation: A preliminary study. Exp Brain Res 2007;181:555–60.** [**https://doi.org/10.1007/s00221-007-0952-x**](https://doi.org/10.1007/s00221-007-0952-x)**.**

***Authors.*** *Thanks for the references. We read the paper from Chiang and agree that the contralateral hemisphere cannot be called a control. We modified our manuscript to study the tDCS effects using fNIRS on the targeted and non-targeted hemispheres. Since our fNIRS montage is limited to sensorimotor cortex so we should have all our channels in the contralateral hemisphere affected by inter-hemispheric connectivity. We have clarified the selection of Pathway 3 for the targeted-region and the Pathway 4 for the nontargeted-region in the section* ***(B) Physiologically detailed model linearization for grey-box analysis using subject-specific fNIRS-tHb data*** *as shown below.*

*“Figure 10B shows that the tDCS perturbation Pathway 4 gave lowest median MSE and medial AIC across all subjects for the grey-box linear model fits at the contralateral nontargeted-region. Here, over-fitting induces variability while under-fitting is known to induce bias so we used Akaike information criterion (AIC) to find a balance (lowest AIC selected). Figure 10C shows that the tDCS perturbation Pathway 3 presented the lowest AIC (median -1.726) across all subjects for the grey-box linear model fits at the targeted-region while Figure 10D shows that the tDCS perturbation Pathway 4 gave the lowest AIC (median -0.915) across all subjects at the contralateral nontargeted-region. Residuals checks were performed based on the autocorrelation curves of the residuals and the cross-correlation curves between input and the residuals – see Figure S3. Therefore, based on MSE and AIC, tDCS perturbation Pathway 3 was selected for the grey-box linear model fits at the targeted-region while Pathway 4 was selected for the contralateral nontargeted-region.”*

**Comment 10: Authors listed the parameters for different models in both main context and supplementary. As one can see different pathway has a different number of parameters. How do authors justify the influence of the number of parameters on model fitting results? As we know, this factor influences a lot the fitting performance (more parameters tend to overfit).**

***Authors.*** *Thanks for your concerns. The models are nested and get more complex going from TF4 (Pathway 4) to TF1 (Pathway 1) as shown below in the Table 3. Here, for a nested model analysis, the Pathway 1 is most complex that incorporated other models (as shown in the Table below) so can overfit if we only look at MSE. Here, reduced dimension grey-box linear model for the Pathway 3 with 9 poles and 2 zeros (all free parameters) provided also the best Chi-Square Goodness-of-Fit of 0.0078 that takes into account nested models ([Cedersund, G. & Roll, J. Systems biology: model based evaluation and comparison of potential explanations for given biological data. The FEBS journal 276, 903-922 (2009)]). Here, Pathway 3 was selected for the targeted-region that consistently performed on whether the model is parsimonious (less complex model better) and conform to the data (goodness of fit) while Pathway 4 was selected for the contralateral nontargeted-region. We have now clarified and discussed this further in the section* ***(C) Reduced dimension grey-box linear model analysis of averaged fNIRS-tHb data*** *as given below:*

*“Fitting of the least complex (model order) Pathway 4 model, TF4, with 7 poles and 1 zero (all free parameters) provided an MSE of 0.0031 and Chi-Square Goodness of Fit of 0.0104. Then, the next complex (model order) Pathway 3 model, TF3, with 9 poles and 2 zeros (all free parameters) provided an MSE of 0.0025 and Chi-Square Goodness of Fit of 0.0078. Then, the next complex (model order) Pathway 2 model, TF2, with 11 poles and 3 zeros (all free parameters) provided an MSE of 0.0025 and Chi-Square Goodness of Fit of 0.0085. The most complex (model order) Pathway 1 model, TF1, with 12 poles and 3 zeros (all free parameters) provided an MSE of 0.0264 and Chi-Square Goodness of Fit of 0.0647. Here, more parameters in more complex (model order) models worsened the Chi-Square Goodness of Fit even with similar MSE, e.g., TF2 higher model order than TF3. Therefore, TF3 for the tDCS perturbation Pathway 3 provided a balance in terms of Chi-Square difference test for nested pathways^90^ for the HD-tDCS-induced initial transient tHb changes within 0.01 and 0.05 Hz.”*

Table 3. Minimal realization transfer functions were obtained through Model Reducer (MATLAB, MathWorks, Inc., USA) for four model pathways from the detailed physiological model for the neurovascular unit compartment model.

| **Pathway transfer functions (TF)** | **Transfer functions from the compartmental neurovascular coupling model that were used for the initial parameterization of the four pathways** | **Parameterization** |
| --- | --- | --- |
| **TF1.**  **Synaptic Potassium → vessel circumference** | $TF1(s)= \frac{1}{(s+0.4)}TF2(s)$ | 11 poles, 3 zeros |
| **TF2.**  **Astrocytic membrane potential → vessel circumference** | $TF2\left( s \right)=\frac{\left( s+46.5 \right)}{\left( s+1.966 \right)\left( s+15.08 \right)} TF3(s)$ | 10 poles, 3 zeros |
| **TF3.**  **Perivascular Potassium → vessel circumference** | $TF3(s)=\frac{\left( s+2.371e07 \right)}{\left( s+2.974e04 \right)\left( s+1 \right)} TF4(s)$ | 8 poles, 2 zeros |
| **TF4.**  **Voltage gated ion channel on SMC → vessel circumference** | $TF4\left( s \right)=\frac{(s+2.962)}{(s+9.594e06)(s+20.69)(s+3.3)(s+0.2446)(s2 + 9.804s + 95.24)}$ | 6 poles, 1 zero |

**Discussion**

**Comment 11: Authors mentioned: “We found that Pathway 3 presented the least MSE, as shown in Figure 4, so the fNIRS-tHb signal is postulated to have a significant representation from the superficial pial vessels.”**

**Regardless of the justification of statistics significant test on evaluating a hypothesis, please avoid saying “significant” without doing a test in Figure 4.**

***Authors.*** *Apologies for the confusion. We have removed the statements of significance and showed the notches in the boxplot (Figure 10 below) to display the variability between samples. The width of a notch is computed so that boxes whose notches do not overlap have different medians at the 5% significance level.*

*(A)*

*(B)*

*(C)*

*(D)*

Figure 10. Boxplot across 11 subjects of the mean square error (MSE) and Akaike Information Criterion (AIC) of grey-box linear model fits for the four tDCS perturbation pathways. Pathway 1: tDCS perturbing vessel response through synaptic potassium pathway, Pathway 2: tDCS perturbing vessel response through the astrocytic pathway, Pathway 3: tDCS perturbing vessel response through perivascular potassium pathway, and Pathway 4: tDCS perturbing vessel response through the SMC pathway. On each box, the central mark indicates the median, and the bottom and top edges of the box indicate the 25th and 75th percentiles, respectively. If the notches in the box plot do not overlap, one can conclude, with 95% confidence that the true medians do differ. The whiskers extend to the most extreme data points not considered outliers, and the outliers are plotted individually using the red '+' symbol. The mean is also shown with a black '+' symbol. (A) MSE for targeted-region. (B) MSE for nontargeted-region. (C) AIC for targeted-region. (D) AIC for nontargeted-region.

*Also, the representation of the superficial pial vessels in the fNIRS signal is based on prior work by [Gagnon L, Yücel MA, Dehaes M, Cooper RJ, Perdue KL, Selb J, Huppert TJ, Hoge RD, Boas DA. Quantification of the cortical contribution to the NIRS signal over the motor cortex using concurrent NIRS-fMRI measurements. Neuroimage. 2012 Feb 15;59(4):3933-40. doi: 10.1016/j.neuroimage.2011.10.054. Epub 2011 Oct 21.]. We have now clarified the statement in the Discussion section as follows*

*“Here, fNIRS-tHb signal without short separation regression may also have a representation from the superficial pial vessels since back-reflection geometry of the fNIRS measurement renders the signal sensitive to pial vasculature[30]. This need further investigation in the future since DOT methods may be able to delineate tDCS effects on the pial vessels from the penetrating arteriole and the first order capillary that may be crucial for investigating the spatiotemporal aspects of the perivascular modulation by tDCS^15^ of the intracerebral versus intracranial blood vessels in the humans.”*

**Comment 12: The authors stated that: “In this study, our goal was to investigate the role of various neuronal and non-neuronal pathways leading to the CVR to tDCS, as demonstrated experimentally by our prior works^42,60,61^, through an objective physiologically constrained grey-box model”.**

**First of all, the authors indeed conduct detailed model selection and explanation based on literature. The models involved in the manuscript also covered the concept of neuronal and non-neuronal pathways. However, the tHb data from fNIRS, is not purely neuronal related signal. Although the authors used PCA to remove the physiological part of the fNIRS signal, but it is not guaranteed to clean all of them. We admit there is no way in continuous-wave fNIRS to remove the physiological noise completely, but such confounding plays a big role in justifying the results and rationale of fitting to fNIRS data for model checking, especially the criteria is MSE. Please also see the details in the comments of the method section.**

***Authors.*** *Thanks for your advice. In fact, the core of the paper is model reduction, model selection, and hypothesis testing based on our data and system biology approach. In our systems biology approach, the core results included the analysis of the response of the physiologically detailed neurovascular coupling (NVC) model. Then, we simplified the physiologically detailed complex model using prediction-error minimization (PEM) algorithm (‘pem’ in MATLAB) to update the parameters to improve the fit fNIRS data set with slow signal changes (<0.1 Hz). Therefore, the grey-box linear model was fitted to the initial 120sec + 30sec ramp-up time = 150sec since 3 minutes following the start of tDCS the aftereffects starts where neuroplastic changes are induced [Nitsche, M. A. & Paulus, W. Excitability changes induced in the human motor cortex by weak transcranial direct current stimulation. The Journal of physiology 527, 633-639 (2000).]*

*Here, the goal is hypothesis testing via model selection using systems biology approach, so, in this study, “model fitting” is used for hypothesis testing of the four pathways on whether the model is parsimonious (less complex model better) and conform to the data (goodness of fit). Under-fitting is known to induce bias while over-fitting induces variability so we used Akaike information criterion (AIC) to find balance (lowest AIC selected) – a concept from systems biology.Therefore, MSE and Chi-Square Goodness-of-Fit (that determines the quality of fit [Cedersund, G. & Roll, J. Systems biology: model based evaluation and comparison of potential explanations for given biological data. The FEBS journal 276, 903-922 (2009)] for such nested models) provided whether the reduced dimension model conform to the data (goodness of fit). Here, Pathway 3 was selected that consistently performed on whether the model is parsimonious (less complex model better) and conform to the data (goodness of fit).*

*Indeed, there is no way to completely remove systemic noise that was more prominent (from PCA) in the non-targeted hemisphere. However, fNIRS signal quality can be determined based on negative correlation between oxygenated and deoxygenated hemoglobin dynamics [Cui X, Bray S, Reiss AL. Functional near infrared spectroscopy (NIRS) signal improvement based on negative correlation between oxygenated and deoxygenated hemoglobin dynamics. Neuroimage. 2010;49(4):3039-3046. doi:10.1016/j.neuroimage.2009.11.050]. We followed this method and rejected 3 subjects before reduced dimension model fitting, as shown below in Table S2.*

*Supplementary Table S2: Oxy-hemoglobin (oxy-Hb), Deoxy-hemoglobin (deoxy-Hb) and Total Hemoglobin (tHb) obtained from NIRS channel in tDCS stimulated region for all the 11 participants. *subjects with >-0.5 Correlation Coefficient between Oxy-Hb & Dxy-Hb*

| Sub # | Normalized oxy-hemoglobin and deoxy-hemoglobin | Correlation Coefficient between oxy-Hb & deoxy-Hb | Total Hemoglobin, tHb (Normalized) |
| --- | --- | --- | --- |
| 1 |  | -0.9007 |  |
| 2 |  | -0.7923 |  |
| 3* |  | -0.0692 |  |
| 4* |  | -0.1900 |  |
| 5 |  | -0.6735 |  |
| 6 |  | -0.8683 |  |
| 7 |  | -0.9759 |  |
| 8 |  | -0.8390 |  |
| 9 |  | -0.8542 |  |
| 10* |  | -0.3768 |  |
| 11 |  | -0.8691 |  |

___________________________________________________________________________

**Method**

**Comment 13: Could authors please check figure 6 carefully? There are several confusions:**

**1) for each hemisphere, A has 6 detectors and 2 sources, but in results and data analysis and figure caption, only 2 detectors**

**were used. Could the authors please explain why the other 4 detectors (F2, F6, P2 and P6 on right hemi) were not used? We understand that they may not give a good response since they might be far from to stimulation site, but then please do not mention them directly in the figure.**

***Authors.*** *Apologies for the confusion. We have improved the Figure 1A as shown below.*

(A)

Figure 1. (A) HD-tDCS-fNIRS montage on a 10/10 EEG system. The fNIRS time series data from the transmitter-receiver optode combinations in the vicinity of 4x1 HD-tDCS electrodes (fNIRS channels encircled in red, left hemisphere: C5-CP3, C1-CP3, C5-FC3, and C1-FC3), i.e. the targeted-region, as well as the non-targeted region in the contralateral hemisphere (fNIRS channels encircled in blue, right hemisphere: C2-FC4, C2-CP4, C6-FC4, and C6-CP4) were used for the grey-box modeling.

**2) how come the sensitivity value was negative? The color bar has the highest value of 0**

***Authors****. Apologies for confusion. The sensitivity values are displayed logarithmically with a default range of 0.01 to 1, or −2 to 0 in log10 units [Aasted CM, Yücel MA, Cooper RJ, et al. Anatomical guidance for functional near-infrared spectroscopy: AtlasViewer tutorial. Neurophotonics. 2015;2(2):020801. doi:10.1117/1.NPh.2.2.020801]. We have now clarified this in the manuscript as shown below in Figure 1B and 1C.*

(B)(C)

Figure 1. (B) Measurement sensitivity of the fNIRS channels at the grey matter on the left hemisphere along with the HD-tDCS electrodes in the Atlas Viewer open-source package (<https://github.com/BUNPC/AtlasViewer>). The sensitivity values are displayed logarithmically by the color scale. (C) Measurement sensitivity of the fNIRS channels at the grey matter on the right hemisphere in the Atlas Viewer open source package (<https://github.com/BUNPC/AtlasViewer>). The sensitivity values are displayed logarithmically by the color scale.

**Comment 14: Authors mentioned, “Due to the lack of short-separation channels to perform short source-detector regression to remove extra-cerebral hemodynamics, we performed data-driven principal component analysis (PCA) to identify the signal components that explained the greatest amount of covariance across all the spatially symmetrically distributed 16 channels”**

**How 16 channels are counted? The previous paragraph clearly said 4 channels on each side. If all channels in Figure 6 (A) were used, then they are indeed 16 channels, but the rest channels are not involved in the results inferences. Could the authors please clarify this?**

***Authors.*** *Apologies for the confusion. Data-driven PCA was used to identify the signal components that explained the greatest amount of covariance across all the spatially symmetrically distributed 16 channels (i.e., based on all NIRS optodes in the montage). This was done as a part of pre-processing for all the channels. However, for model analysis, we considered targeted-region and nontargeted-region, and therefore selected the four channels from each hemisphere following pre-processing for all the channels as shown below in Figure 1A.*

(A)

Figure 1. (A) HD-tDCS-fNIRS montage on a 10/10 EEG system. The fNIRS time series data from the transmitter-receiver optode combinations in the vicinity of 4x1 HD-tDCS electrodes (fNIRS channels encircled in red, left hemisphere: C5-CP3, C1-CP3, C5-FC3, and C1-FC3), i.e. the targeted-region, as well as the non-targeted region in the contralateral hemisphere (fNIRS channels encircled in blue, right hemisphere: C2-FC4, C2-CP4, C6-FC4, and C6-CP4) were used for the grey-box modeling.

**It is always good to find a way to remove extra-cerebral hemodynamics even if no short-separation channels are available from the hardware side. Considering the importance of the potential confounding from extra-cerebral hemodynamics on the justification of the model evaluation of this manuscript, it would be nice if the authors could compare the results with/without PCA, to show at least there is a difference on the model evaluation side. For instance, without PCA procedure, the results on model evaluation should be different, or the difference between pathways should be diluted since extra-cerebral hemodynamics are involved.**

**Alternatively, the authors could have performed analysis on the component from PCA that was believed containing extra-cerebral hemodynamics, and then compare it with the current results. It has been shown in [4], without careful analysis, TMS induced hemodynamics measured on the scalp could have been very similar to the one when stimulating the shoulder.**

**A recent review [5] of TMS/fNIRS also addressed the necessity of using short-channel to remove the extra-cerebral hemodynamics for more reliable results. We understand tDCS may not induce fNIRS signal changes in the scalp (by contracting muscles) as high as TMS. Pleases note that this does not mean we believe authors have to have short-channels and does not mean we believe that short-channel is the best approach, but it means no matter what approach is used to remove the confounding, it is necessary to prove it worked on the final results, meaning with/without it would lead to different results that are as expected.**

**[4] Näsi T, Mäki H, Kotilahti K, Nissilä I, Haapalahti P, Ilmoniemi RJ. Magnetic-stimulation-related physiological artifacts in hemodynamic near-infrared spectroscopy signals. PLoS One 2011;6. https://doi.org/10.1371/journal.pone.0024002.**

**[5] Curtin A, Tong S, Sun J, Wang J, Onaral B, Ayaz H. A systematic review of integrated functional near-infrared spectroscopy (fNIRS) and transcranial magnetic stimulation (TMS) studies. Front Neurosci 2019;13.** [**https://doi.org/10.3389/fnins.2019.00084**](https://doi.org/10.3389/fnins.2019.00084)**.**

***Authors.*** *Thanks for your concerns. We checked without the PCA approach based on the Correlation Coefficient between Oxy-Hb & Dxy-Hb which significantly deteriorated. This is based on the rationale that if the measured oxy-Hb and dxy-Hb signals are not strongly negatively correlated, it’s likely that the signals contain substantial noise [Cui X, Bray S, Reiss AL. Functional near infrared spectroscopy (NIRS) signal improvement based on negative correlation between oxygenated and deoxygenated hemoglobin dynamics. Neuroimage. 2010 Feb 15;49(4):3039-46. doi: 10.1016/j.neuroimage.2009.11.050]. Subjects with greater than -0.5 Correlation Coefficient between Oxy-Hb & Dxy-Hb are now highlighted in the manuscript as well as the supplementary materials.*

| Sub # | Correlation Coefficient with PCA | Correlation Coefficient without PCA |
| --- | --- | --- |
| 1 | -0.90 | -0.32 |
| 2 | -0.79 | -0.28 |
| 3* | -0.07 | -0.04 |
| 4* | -0.19 | -0.07 |
| 5 | -0.67 | -0.23 |
| 6 | -0.87 | -0.34 |
| 7 | -0.98 | -0.47 |
| 8 | -0.84 | -0.36 |
| 9 | -0.85 | -0.31 |
| 10* | -0.38 | -0.16 |
| 11 | -0.87 | -0.38 |

*Since our experimental set-up lacked short-separation channels to perform short source-detector regression, therefore, we have performed data-driven principal component analysis (PCA) to identify the signal components that explain the greatest amount of covariance across all the spatially symmetrically distributed 16 channels. However, there is no way to completely remove systemic noise. We have now clarified this limitation and discussed this further in the Discussion section as given below:*

*“Due to the lack of short-separation channels to perform short source-detector regression to remove extra-cerebral hemodynamics, we performed a data-driven principal component analysis to identify the extra-cerebral signal components that explained the most amount of covariance across all the 16 spatially symmetrically distributed fNIRS channels. We also adopted the anti-correlation method[29] (see Table S2) to confirm fNIRS signal quality since the short-separation channels were not available.”*

**Comment 15: The authors said “The neurovascular and neurometabolic coupling-related hemodynamic response should lead to an initial increase in deoxy-Hb and an equal decrease in oxy-Hb, as shown by Devor et al.^10^. Then, the blood volume should start to increase about 600ms following the neural stimulus10. Such differential activation of oxy-Hb and deoxy-Hb was found in the vicinity of 4x1 HD-tDCS electrodes: C5-CP3, C1-CP3, C5-FC3, and C1-FC3, that indicated neurovascular coupling related hemodynamic response by HD-tDCS current density at the ipsilateral primary motor cortex.”**

**We do not understand why always mention the initial dip, and we did not see the dip in fNIRS results. How can we see 600ms changes in a figure that has a time scale of 10 minutes? Could the authors please show a zoom-in figure to prove the finding of an initial dip in fNIRS?**

***Authors.*** *Apologies for the confusion. Initial dip in tHb (related to blood volume) was found in the 0-150sec in few subjects as shown below in the new Figure 11 of the manuscript:*

*Figure 11. Oxy-hemoglobin (Oxy-Hb), deoxy-hemoglobin (Dxy-Hb), and total-hemoglobin (tHb) time series at the targeted-region during anodal HD-tDCS for the subjects P3, P4, P10 who were rejected due to poor anti-correlated Oxy-Hb & Dxy-Hb measures of -0.0692, -0.1900, and -0.3768 respectively. These subjects also demonstrated an initial dip in the tHb (also, Oxy-Hb & Dxy-Hb) time series that is highlighted with a black ellipse in the Oxy-Hb & Dxy-Hb time series.*

**Comment 16: In general, the font in the figures is a bit small. Please make them larger if not too much work. Otherwise, they are readable after zooming.**

***Authors*.** *Thanks for your advice. We have tried to improve the readability of all the figures.*

--------------------------------------------------------------

**Reply to comments of Reviewer 3 (Review_comments.pdf)**

**Summary: The article describes a complete study about the effect of electrical stimulation on hemodynamics, more precisely vessel circumference and total hemoglobin, by means of a model –data comparison of 11 human participants. The original published physiological model include neuronal, astrocyte, perivascular and smooth muscle cells compartments tightly coupled.**

**The innovation of the study (apart from new data compared to the published pilot study) lies in the methodological part: The interesting biological point is that the authors highlight the four pathways by which electrical stimulation affects the cerebral blood flow via temporal dynamics of the vessel circumference. In order to better identify and understand these contributions separately, the authors choose to linearize and reduce the physiological model to transfer functions to obtain a grey-box linear model, and then compare it to individual participant’s data and to the average.**

**In my opinion the definition of these four pathways and above all the model linearization and reduction are crucial points that make the strength of the idea and thus an interesting study to publish. Nonetheless, these methodological points are difficult to understand under the present form of the article: on one hand because of the organization of the document and on the other hand because of a lack precision about how are implemented the pathways (equations) and how is realized the post –processing of the model (linearization and reduction (Fig. 9B and 10), as indicated in the three first sections of this review.**

**I specify here that my review concerns essentially the model computing part of the article as I’m an expert neither in brain electrical stimulation nor experimental protocols.**

**Major issue #1: Reorganization of the manuscript**

**I know that guidelines of the journal encourage to write the results part before the methods one, but in this article, doing it the classical way would greatly improve the direct understanding of the framework imagined by par the authors. At the moment we begin to understand the approach only with Fig. 10 at first reading of the document.**

**In a new organization, I would keep content of Fig. 1 in the beginning with methods as I understood were acquired in a preceding pilot study and are thus here inputs to the questioning. Apart from the first §, sub-section A in methods section is the acquisition protocol and the most important part are the data used for the estimation/identification (Fig.1) thus the details of this section may be placed in the Suppl. Mat. to only keep the essentials parts in the main text, except details of processing concerning frequency matters. Also sub-section C**

**« physiologically detailed model linearization for grey-box analysis of experimental data » in results section contains in my opinion a majority of methodological content except the ultimate sentence so that its position in the manuscript should be reviewed.**

**If the authors want to keep the present organization, I strongly recommend to add a scheme of the whole framework in order to clarify the different parts of the manuscript.**

***Authors.*** *Thank you for your suggestions. We have reorganized the contents in the updated manuscript. We have placed Methods section in the beginning after Introduction, then followed by Results and Discussion.*

**Major issue #2: How is obtained Fig. 2B Reading the 17 equations of the physiological model, I found that the effects contribute to vessel circumference *x* via the *[Ca^2+^]* concentration (or membrane potential V_m_) in the SMC compartment. Therefore I don’t catch how the authors created the separated contributions of the four pathways in Fig. 2B as they mentioned:**

** First order function for each pathway in Fig. 9B leading to contribution variables *([K^+^]_s_, V_k_, [K^+^]_p_, V_m_*) but a joined vessel function for *x* (as I suppose);**

***Authors.*** *Apologies for the confusion. tDCS effects are perturbation in each of the four compartments. The table below depicts the state equations for different compartments. The detailed equations based on the literature studies are given in Supplementary Materials.*

*The NVU compartmental pathways are nested that we have clarified now in the section:* **(D) Physiologically detailed Neurovascular Compartmental Dynamics**

*“Our goal was mechanistic grey-box modeling for hypothesis testing, where the hypotheses are formulated as a set of mathematical equations for data fitting to identify core predictions from biological criteria^81^. We identified four nested NVU compartmental pathways (see Figure 6) where tDCS perturbed a state variable at each of the four NVU compartments (see Table 2 for details on compartmental state variables) for hypothesis testing of the initial (0-150sec) tDCS effects on the NVU leading to blood vessel (circumference) volume changes. Figure 6 shows the nested model for each of the four pathways starting from tDCS scalp current density that perturbed synaptic potassium (K+) released from active neurons (∆*$J_{K_{s}}$*) for Pathway 1, astrocytic transmembrane current (∆*$I_{T}$*) for Pathway 2, perivascular potassium (K+) concentration (*${\Delta\left[ K^{+} \right]}_{T}$*) for Pathway 3, and voltage-gated ion channel current (∆*$I_{KV}$*) on the smooth muscle cell (SMC) for Pathway 4.”*

*Here, each of the four perturbations are the input and the change in the vessel circumference ∆x is the output that is proportional to the vessel volume as shown in Figure 6 below.*

Figure 6. Schematic representation of the four pathways for tDCS current density effects (perturbation) on vessel volume function related to vessel circumference changes for hypothesis testing using experimental total hemoglobin (tHb) concentration changes (proportional to blood volume). The four simulated pathways are, Pathway 1: tDCS current density (input pulse) via first-order transfer function modulate vessel volume response (output) by perturbing synaptic potassium (K+) released from active neurons (∆$\boldsymbol{J}_{\boldsymbol{K}_{\boldsymbol{s}}}$), Pathway 2: tDCS current density (input pulse) via first-order transfer function modulate vessel volume response (output) by perturbing the astrocytic transmembrane current (∆$\boldsymbol{I}_{\boldsymbol{T}}$), Pathway 3: tDCS current density (input pulse) via first-order transfer function modulate vessel volume response (output) by perturbing perivascular potassium (K+) concentration (${\boldsymbol{\Delta}\left[ \boldsymbol{K}^{\boldsymbol{+}} \right]}_{\boldsymbol{T}}$), and Pathway 4: tDCS current density (input pulse) via first-order transfer function modulate vessel volume response (output) by perturbing voltage-gated ion channel current (∆$\boldsymbol{I}_{\boldsymbol{KV}}$) on the smooth muscle cell (SMC).

*The model was simulated considering the four inputs (tDCS perturbation) independently, corresponding to the proposed pathways of tDCS effects on the vessel response for model fitting (to fNIRS tHb changes). We have now clarified this as well as the four tDCS perturbation pathways as shown in Figure 6 above.*

** “vasoactive signals for the pathways” in the ultimate § of sub-section (B) of the Results but without any reference to an equation or specification in the corresponding Fig. 2B caption;**

***Authors.*** *Thank you for your suggestions. We considered tDCS perturbation in each of the four pathways as the vasoactive signal in the Witthoft and Karniadakis^42^ model. We have clarified this in the text,*

“So, the tDCS current density at the scalp ($I_{tdcs}$) was proportional to the current density ($J_{tDCS})$in the neurovascular brain tissue based on a lead field matrix (the forward solution; see Figure S4) leading to the vasoactive signal (state perturbation to NVU compartments) via first order transfer function,

$v_{i}= \frac{K_{i}}{s/\tau+1}I_{tdcs}$	*(i)*

where K_i_ is arbitrary gain from lead field matrix ($J_{tDCS}={K_{i}\cdot I}_{tdcs}$), and$\tau$is the time constant. Here, the state variables of various NVU compartments modeled in the study are listed in Table 2, while the detailed equations are provided in the supplementary materials (see Text S1).”

*Then, we have described with references the nature of tDCS’s vasoactive signal in each pathway as follows,*

**“(a) Pathway 1:** tDCS perturbation of synaptic potassium leading to vessel circumference changes

Studies have shown that K+ can act as a potent vasodilator signal that couples local neuronal activity to vasodilation in the brain, and have a major role in cerebrovascular mechanisms^83-86^. Studies have shown that the potassium pathway is responsible for the fast onset of vasodilation compared to the other mediators^59,60,87^. Synaptic activity was assumed to be modulated by tDCS current density^82^ that affected K+ release from active neurons into the synaptic space. Here, $J_{K_{s}}$ is the K+ released from active neurons that is considered to be perturbed by tDCS from its baseline condition such that $\Delta J_{K_{s}}=v_{1}= \frac{K_{1}}{s/\tau+1}I_{tdcs}$using equation (i). The potassium concentration in the synaptic space, $\left[ K^{+} \right]_{s}$ is then given as (see equation 1 in the Text S1):

$\frac{d{[K^{+}]}_{s}}{dt}=$ $J_{K_{s}}\boldsymbol{+}\frac{K_{1}}{s/\tau+1}I_{tdcs}- J_{\Sigma Kmax}k_{Na}\frac{{[K^{+}]}_{s}}{{[K^{+}]}_{s}+ {KKO}_{a}}$	*(ii)*

where $J_{K_{s}}$is the baseline flux of K+ in the synaptic space, $J_{\Sigma Kmax}$ is the maximum flux, $k_{Na}$ is a constant parameter that depends on extracellular sodium concentration, ${KKO}_{a}$is the threshold value for K+ concentration in the synaptic space, ${[K^{+}]}_{s}$.

**(b) Pathway 2:** tDCS perturbation of the astrocytic transmembrane current leading to vessel circumference changes

Astrocytes are susceptible to small variations in their membrane potential^71^ and their long processes are sensitive to polarization by tDCS^72-74^. Here, astrocytic transmembrane current ($I_{T}$) was perturbed by tDCS, $\Delta I_{T}=v_{2}= \frac{K_{2}}{s/\tau+1}I_{tdcs}$from equation (i) that was added to other transmembrane currents including $I_{BK}$ is current through Big Potassium (BK) channel, $I_{leak}$is leak current, $I_{TRPV}$is electrical current through the TRPV channel and $I_{\Sigma K}$ is the electrical current carried by the K^+^ influx at the perisynaptic process (equation 21 in the Text S1):

$\frac{dV_{k}}{dt}= \frac{1}{C_{astr}}\left( {-I}_{BK}-I_{leak}- I_{TRPV}- I_{\Sigma K}+\frac{K_{2}}{s/\tau+1}I_{tdcs} \right)$	*(iii)*

**(c) Pathway 3:** tDCS perturbation of perivascular potassium concentration leading to vessel circumference changes

Glial cells maintain extracellular K+ concentration by the imbalance in their membrane polarity and can affect K+ spatial buffering affecting tDCS modulation^36,77^. Astrocytic release of K+, via two potassium channels (BK and KIR), into the perivascular space can be perturbed by tDCS. Astrocytic role in neurovascular coupling may be related to the strength of stimulation where high strength can lead to vasoconstriction[19] (mediated via K+ and EET signalling[21]). Vasoconstriction can follow vasodilation when the astrocytic calcium concentration (or, perivascular K+ concentration) increase above a certain threshold. We assumed that low-intensity tDCS perturbation would not cross that threshold where ${\Delta\left[ K^{+} \right]}_{T}=v_{3}= \frac{K_{3}}{s/\tau+1}I_{tdcs}$. So, the perivascular potassium concentration,$\left[ K^{+} \right]_{P}$, is given as (equation 26, Text S1):

$\frac{d\left[ K^{+} \right]_{P}}{dt}= \frac{J_{BK}}{{VR}_{pa}}+ \frac{J_{KIR}}{{VR}_{ps}}- R_{decay}\left( \left[ K^{+} \right]_{P}- \left[ K^{+} \right]_{P,min} \right)+\frac{K_{3}}{s/\tau+1}I_{tdcs}$	*(iv)*

Here, ${[K^{+}]}_{P,min}$ is the resting state equilibrium K^+^ concentration in the perivascular space. The K+ flow from the astrocyte and SMC are $J_{BK}$ and $J_{KIR}$corresponding to BK and inward rectifying potassium (KIR) respectively. And, ${VR}_{pa}$ and ${VR}_{ps}$ are the volume ratios of perivascular space to astrocyte and SMC, respectively. $R_{decay}$ is the rate at which perivascular K^+^ concentration decays to its baseline state.

**(d) Pathway 4:** tDCS perturbation of voltage-gated ion channel current on the smooth muscle cell leading to vessel circumference changes

Smooth muscle cell (SMC) voltage-gated potassium (KV) channels and inwardly rectifying K+ channels are important in penetrating arterioles that control arterial diameter by exerting a major hyperpolarizing influence[22]. Therefore, tDCS electric field can perturb voltage gated potassium current ($\Delta I_{KV}=v_{4}= \frac{K_{4}}{s/\tau+1}I_{tdcs}$) that was added to other currents including I_L_, I_K_, I_Ca_ and I_KIR_ that represent leak, K+, Ca2+, and KIR channel currents respectively in the SMC compartment. Then, the SMC membrane potential, V_SMC_, is given by (equation 41, Text S1):

$\frac{dV_{SMC}}{dt}= \frac{1}{C_{SMC}}\left( -I_{L}- I_{K}-I_{Ca}-I_{KIR}- I_{KV}+\frac{K_{4}}{s/\tau+1}I_{tdcs} \right)$	*(v)*

Here, the four tDCS perturbation pathways are nested, i.e., the pathway 1 is represented by seventeen ordinary differential equations starting from synaptic K+ (see Text S1) that nested other tDCS perturbation pathways 2-4 – see Figure 6.”

** Confusing sentences in the section B of Suppl. Mat., highlighting the pathways: for instance “*I_KV_* postulated to be driven by current density” means to me *I_KV_* = *function (J_tDCS_)* and is coherent with the ultimate sentence about solving the model. Then how to obtain different contributions to variable *x* knowing that all finish in *V_m_* equation with *([K^+^]_s_, V_k_, [K^+^]_p_, V_m_*) variables strongly coupled?**

**Another explanation relies later in the sentence as “a first order transfer function for its vasoactive signal means” which means to me *v_i_* = *TF (J_tDCS_)*. In this case, how are defined the different contributions *x_i_* = *TF (v_i_)* of Fig. 2B?**

**I highly recommend to specify these points because it’s a reproduction issue. Also Fig. 2B should be improved in terms of temporal resolution.**

***Authors.*** *Apologies for the confusion. The model was simulated considering the four tDCS perturbations independently in the four compartments, corresponding to the proposed pathways for evaluating the vessel response. The neurovascular system is based on the published literature (Witthoft and Karniadakis^42^ model). For systems analysis, the tDCS perturbation in the four different compartments was considered input and the nested pathways were simulated for the output (vessel response). This is now clarified in* ***Methods section D:*** *Physiologically detailed Neurovascular Compartmental Dynamics as:*

“*Our goal was mechanistic grey-box modeling for hypothesis testing, where the hypotheses are formulated as a set of mathematical equations for data fitting to identify core predictions from biological criteria^92^. We identified four nested NVU compartmental pathways (see Figure 6) where tDCS perturbed a state variable at each of the four NVU compartments (see Table 2 for details on compartmental state variables) for hypothesis testing of the initial (0-150sec)tDCS effects on the NVU leading to blood vessel (circumference) volume changes. Figure 6 shows the nested model for each of the four pathways starting from tDCS scalp current density that perturbed synaptic potassium (K+) released from active neurons (∆*$J_{K_{s}}$*) for Pathway 1, astrocytic transmembrane current (∆*$I_{T}$*) for Pathway 2, perivascular potassium (K+)concentration (*${\Delta\left[ K^{+} \right]}_{T}$*) for Pathway 3, and voltage-gated ion channel current (∆*$I_{KV}$*) on the smooth muscle cell (SMC) for Pathway 4.”*

*We have also added the description of pathway specific model equations in* ***Methods section D:*** *Physiologically detailed Neurovascular Compartmental Dynamics.*

*“So, the tDCS current density at the scalp (*$I_{tdcs}$*) was proportional to the current density (*$J_{tDCS})$*in the neurovascular brain tissue based on a lead field matrix (the forward solution; see Text S2) leading to the vasoactive signal (state perturbation to NVU compartments) via first order transfer function,*

$v_{i}= \frac{K_{i}}{s/\tau+1}I_{tdcs}$	*(i)*

*where K_i_ is arbitrary gain from lead field matrix (*$J_{tDCS}={K_{i}\cdot I}_{tdcs}$*), and*$\tau$*is the time constant. Here, the state variables of various NVU compartments modeled in the study are listed in Table 2, while the detailed equations are provided in the supplementary materials (see Text S1).*

***(a) Pathway 1:*** *tDCS perturbation of synaptic potassium leading to vessel circumference changes*

*Studies have shown that K+ can act as a potent vasodilator signal that couples local neuronal activity to vasodilation in the brain, and have a major role in cerebrovascular mechanisms^83-86^. Studies have shown that the potassium pathway is responsible for the fast onset of vasodilation compared to the other mediators^59,60,87^. Synaptic activity was assumed to be modulated by tDCS current density^82^ that affected K+ release from active neurons into the synaptic space. Here,* $J_{K_{s}}$ *is the K+ released from active neurons that is considered to be perturbed by tDCS from its baseline condition such that* $\Delta J_{K_{s}}=v_{1}= \frac{K_{1}}{s/\tau+1}I_{tdcs}$*using equation (i). The potassium concentration in the synaptic space,* $\left[ K^{+} \right]_{s}$ *is then given as (see equation 1 in Text S1):*

$\frac{d{[K^{+}]}_{s}}{dt}=$ $J_{K_{s}}\boldsymbol{+}\frac{K_{1}}{s/\tau+1}I_{tdcs}- J_{\Sigma Kmax}k_{Na}\frac{{[K^{+}]}_{s}}{{[K^{+}]}_{s}+ {KKO}_{a}}$	*(ii)*

*where* $J_{K_{s}}$*is the baseline flux of K+ in the synaptic space,* $J_{\Sigma Kmax}$ *is the maximum flux,* $k_{Na}$ *is a constant parameter that depends on extracellular sodium concentration,* ${KKO}_{a}$*is the threshold value for K+ concentration in the synaptic space,* ${[K^{+}]}_{s}$*.*

***(b) Pathway 2:*** *tDCS perturbation of the astrocytic transmembrane current leading to vessel circumference changes*

*Astrocytes are susceptible to small variations in their membrane potential^71^ and their long processes are sensitive to polarization by tDCS^72-74^. Here, astrocytic transmembrane current (*$I_{T}$*) was perturbed by tDCS,* $\Delta I_{T}=v_{2}= \frac{K_{2}}{s/\tau+1}I_{tdcs}$*from equation (i) that was added to other transmembrane currents including* $I_{BK}$ *is current through Big Potassium (BK) channel,* $I_{leak}$*is leak current,* $I_{TRPV}$*is electrical current through the TRPV channel and* $I_{\Sigma K}$ *is the electrical current carried by the K^+^ influx at the perisynaptic process (equation 21 in the Text S1):*

$\frac{dV_{k}}{dt}= \frac{1}{C_{astr}}\left( {-I}_{BK}-I_{leak}- I_{TRPV}- I_{\Sigma K}+\frac{K_{2}}{s/\tau+1}I_{tdcs} \right)$	*(iii)*

***(c) Pathway 3:*** *tDCS perturbation of perivascular potassium concentration leading to vessel circumference changes*

*Glial cells maintain extracellular K+ concentration by the imbalance in their membrane polarity and can affect K+ spatial buffering affecting tDCS modulation^36,77^. Astrocytic release of K+, via two potassium channels (BK and KIR), into the perivascular space can be perturbed by tDCS. Astrocytic role in neurovascular coupling may be related to the strength of stimulation where high strength can lead to vasoconstriction[19] (mediated via K+ and EET signalling[21]). Vasoconstriction can follow vasodilation when the astrocytic calcium concentration (or, perivascular K+ concentration) increase above a certain threshold. We assumed that low-intensity tDCS perturbation would not cross that threshold where* ${\Delta\left[ K^{+} \right]}_{T}=v_{3}= \frac{K_{3}}{s/\tau+1}I_{tdcs}$*. So, the perivascular potassium concentration,*$\left[ K^{+} \right]_{P}$*, is given as (equation 26, Text S1):*

$\frac{d\left[ K^{+} \right]_{P}}{dt}= \frac{J_{BK}}{{VR}_{pa}}+ \frac{J_{KIR}}{{VR}_{ps}}- R_{decay}\left( \left[ K^{+} \right]_{P}- \left[ K^{+} \right]_{P,min} \right)+\frac{K_{3}}{s/\tau+1}I_{tdcs}$	*(iv)*

*Here,* ${[K^{+}]}_{P,min}$ *is the resting state equilibrium K^+^ concentration in the perivascular space. The K+ flow from the astrocyte and SMC are* $J_{BK}$ *and* $J_{KIR}$*corresponding to BK and inward rectifying potassium (KIR) respectively. And,* ${VR}_{pa}$ *and* ${VR}_{ps}$ *are the volume ratios of perivascular space to astrocyte and SMC, respectively.* $R_{decay}$ *is the rate at which perivascular K^+^ concentration decays to its baseline state.*

***(d) Pathway 4:*** *tDCS perturbation of voltage-gated ion channel current on the smooth muscle cell leading to vessel circumference changes*

*Smooth muscle cell (SMC) voltage-gated potassium (KV) channels and inwardly rectifying K+ channels are important in penetrating arterioles that control arterial diameter by exerting a major hyperpolarizing influence[22]. Therefore, tDCS electric field can perturb voltage gated potassium current (*$\Delta I_{KV}=v_{4}= \frac{K_{4}}{s/\tau+1}I_{tdcs}$*) that was added to other currents including I_L_, I_K_, I_Ca_ and I_KIR_ that represent leak, K+, Ca2+, and KIR channel currents respectively in the SMC compartment. Then, the SMC membrane potential, V_SMC_, is given by (equation 41, Text S1):*

$\frac{dV_{SMC}}{dt}= \frac{1}{C_{SMC}}\left( -I_{L}- I_{K}-I_{Ca}-I_{KIR}- I_{KV}+\frac{K_{4}}{s/\tau+1}I_{tdcs} \right)$	*(v)*

*Here, the four tDCS perturbation pathways are nested, i.e., the pathway 1 is represented by seventeen ordinary differential equations starting from synaptic K+ (see Text S1) that nested other tDCS perturbation pathways 2-4 – see Figure 6.”*

*Thank you for your suggestion. We have updated Figure 2(B) (Figure 8 now) with the simulation of the physiologically detailed neurovascular coupling model (as recommended by Reviewer 2 too).*

(A)(B)

Figure 8. Physiologically detailed neurovascular coupling model showing the input HD-tDCS current (A) and the output vessel circumference response for the four simulated pathways for duration 0-150sec (B). Pathway 1: tDCS current density perturbing vessel circumference through synaptic potassium pathway, Pathway 2: tDCS current density perturbing vessel circumference through the astrocytic pathway, Pathway 3: tDCS current density perturbing vessel circumference through perivascular potassium pathway, and Pathway 4: tDCS current density perturbing vessel circumference through smooth muscle cell voltage-gated ion current channels pathway. Shown in (B), all the tDCS perturbation pathways has an initial transient response, and then generated steady-state vessel oscillations.

**The question arising then if the four contributions lead to the same dimension, why making the reduction of the model and not just compare the MSE values to conclude about pathway 3 as the more important contribution? This issue is also link to the improvement of methodological figures, as suggested in the next section.**

***Authors.*** *Thank you for your concerns. The four pathways lead to different dimensions from individual fitting as shown in Table S3: System identification using experimental data (total hemoglobin changes from stimulated hemisphere) from 11 subjects (Sub). * subjects with >-0.5 Correlation Coefficient between Oxy-Hb & Dxy-Hb.*

| **Pathway** | **Sub#** | **System identification using experimental data** |
| --- | --- | --- |
| 1: Synaptic Potassium → vessel circumference | 1 | 7.2083e-09 (s+5.055e07) (s+9.594e06) (s+2.974e04) (s+1648) (s+50) (s+20.69)(s+15.08) (s+3.836) (s+0.003298) (s^2^ + 9.803s + 95.24) /  (s+9.594e06) (s+2.974e04) (s+50) (s+20.69) (s+15.08) (s+6.852) (s+0.05234)(s+0.005084) (s^2^ + 2.415e-08s + 15.47)(s^2^ + 9.805s + 95.26) |
|  | 2 | 4.822e-09 (s+9.796e07) (s+9.594e06) (s+2.974e04) (s-1237) (s+50) (s+20.69) (s+15.08) (s+3.857) (s-0.7089) (s^2^ + 9.803s + 95.24) /  (s+9.594e06) (s+2.974e04) (s+50) (s+20.69) (s+15.08) (s+0.1084)  (s^2^ + 6.595s + 11.29) (s^2^ + 0.2092s + 2.423) (s^2^ + 9.802s + 95.29) |
|  | 3* | 5.9575e-08 (s+9.594e06) (s+7.211e06) (s+2.974e04) (s+5472) (s+50) (s+20.69) (s+15.08) (s+3.588) (s+2.638) (s^2^ + 9.804s + 95.24) /  (s+9.594e06) (s+2.974e04) (s+50) (s+20.69) (s+15.08) (s+0.07776)  (s^2^ + 0.01736s + 12.99) (s^2^ + 1.421s + 44.43) (s^2^ + 15.2s + 103.7) |
|  | 4* | 4.6202e-08 (s+9.594e06) (s+9.058e06) (s+2.974e04) (s+596) (s+50) (s+20.69) (s+15.08) (s+3.648) (s+0.01155) (s^2^ + 9.804s + 95.24) /  (s+9.594e06) (s+2.974e04) (s+50) (s+20.69) (s+15.08) (s+0.002497)  (s^2^ + 8.485e-07s + 0.4953) (s^2^ + 6.908s + 15.38) (s^2^ + 9.804s + 95.24) |
|  | 5 | 1.0846e-08 (s+3.876e07) (s+9.594e06) (s+2.974e04) (s+80.58) (s+50) (s+20.69) (s+15.08) (s+3.823) (s-7.563e-05) (s^2^ + 9.803s + 95.24) /  (s+9.594e06) (s+2.974e04) (s+50) (s+20.69) (s+15.08) (s+1.722e-05)  (s^2^ + 0.276s + 0.02161) (s^2^ + 6.635s + 14.04) (s^2^ + 9.804s + 95.24) |
|  | 6 | 8.4064e-09 (s+4.164e07) (s+9.594e06) (s+2.974e04) (s+507.6) (s+50) (s+20.69) (s+15.08) (s+3.828) (s+0.009477) (s^2^ + 9.803s + 95.24) /  (s+9.594e06) (s+2.974e04) (s+50) (s+20.69) (s+15.08) (s+6.532) (s+0.06082) (s+0.0003971) (s^2^ + 0.4731s + 22.34)(s^2^ + 9.649s + 85.98) |
|  | 7 | 8.2796e-10 (s+4.772e08) (s+9.594e06) (s+2.974e04) (s+1616) (s+50) (s+20.69) (s+15.08) (s+3.875) (s+0.04567) (s^2^ + 9.803s + 95.24) /  (s+9.594e06) (s+2.974e04) (s+50) (s+20.69) (s+15.08) (s+6.809)  (s^2^ + 0.09585s + 0.002608) (s^2^ + 2.217e-08s + 14.98) (s^2^ + 9.81s + 95.48) |
|  | 8 | 1.1834e-08 (s+3.708e07) (s+9.594e06) (s+2.974e04) (s-4498) (s+50) (s+20.69) (s+15.08) (s+3.8) (s-1.758) (s^2 + 9.803s + 95.24) / (s+9.594e06) (s+2.974e04) (s+50) (s+20.69) (s+15.08) (s+0.07725)  (s^2^ + 0.007259s + 12.8) (s^2^ + 0.001041s + 18.85) (s^2^ + 16.63s + 150) |
|  | 9 | 1.5696e-08 (s+2.334e07) (s+9.594e06) (s+2.974e04) (s+50) (s+20.69) (s+18.98) (s+15.08) (s+3.786) (s+0.03044) (s^2^ + 9.803s + 95.24) /  (s+9.594e06) (s+2.974e04) (s+50) (s+20.69) (s+15.08) (s+0.003341)  (s^2^ + 0.07064s + 0.03265) (s^2^ + 6.837s + 15.35) (s^2^ + 9.804s + 95.24) |
|  | 10* | 1.2334e-08 (s+3.056e07) (s+9.594e06) (s+2.974e04) (s+4715) (s+50) (s+20.69) (s+15.08) (s+3.809) (s+0.111) (s^2^ + 9.803s + 95.24) /  (s+9.594e06) (s+2.974e04) (s+50) (s+20.69) (s+15.08) (s+3.541) (s+0.2731) (s+0.04935) (s^2^ + 12.52s + 78.44) (s^2^ + 0.3272s + 50.04) |
|  | 11 | -1.2815e-08 (s-3.273e07) (s+9.594e06) (s-4.243e04) (s+2.974e04) (s+50) (s+20.69) (s+15.08) (s+3.815) (s-1.034) (s^2^ + 9.803s + 95.24) /  (s+9.594e06) (s+2.974e04) (s+50) (s+20.69) (s+15.08) (s+0.08326) (s^2^ + 0.003256s + 22.73) (s^2^ + 3.299e-05s + 38.62) (s^2^ + 16.63s + 200.9) |
| **Pathway** | **Sub#** | **System identification using experimental data** |
| 2: Astrocytic Current channel → vessel circumference | 1 | -5.3726e-06 (s+9.594e06) (s-6.453e05) (s+2.974e04) (s+917.7) (s+50) (s+20.69) (s+15.08) (s+0.6542) (s^2^ + 9.785s + 95.61) /  (s+9.594e06) (s+2.974e04) (s+50) (s+20.69) (s+15.08) (s+11.63) (s+0.03122) (s^2^ + 0.07727s + 24.06) (s^2^ + 4.578s + 93.38) |
|  | 2 | 5.5342e-05 (s+9.594e06) (s+2.974e04) (s+50) (s+20.69) (s+15.08) (s+1.443) (s^2^ + 9.779s + 95.52) (s^2^ + 5.64e04s + 6.499e10) /  (s+9.594e06) (s+2.974e04) (s+50) (s+20.69) (s+15.08) (s+8.423) (s+0.1029) (s^2^ + 0.002347s + 90.08) (s^2^ + 7.79s + 1.131e04) |
|  | 3* | 3.6864e-07 (s+1.048e07) (s+9.594e06) (s+2.974e04) (s-3365) (s+50) (s+20.69) (s+15.08) (s-1.143) (s^2^ + 9.808s + 95.37) /  (s+9.594e06) (s+2.974e04) (s+50) (s+20.69) (s+16.15) (s+15.08) (s+0.1192) (s^2^ + 0.03886s + 8.511) (s^2^ + 4.124e-08s + 159.9) |
|  | 4* | -2.0486e-06 (s+9.594e06) (s-1.855e06) (s+2.974e04) (s+2.471e04) (s+50) (s+20.69) (s+15.08) (s+0.004899) (s^2^ + 9.799s + 95.46) /  (s+9.594e06) (s+2.974e04) (s+50) (s+20.69) (s+16.12) (s+15.09) (s+0.002483) (s^2^ + 0.1811s + 13.56) (s^2^ + 1.997e-05s + 153.9) |
|  | 5 | -5.8294e-06 (s+9.594e06) (s-6.845e05) (s+2.974e04) (s+5377) (s+50) (s+20.69) (s+15.08) (s+0.07949) (s^2^ + 9.786s + 95.6) /  (s+9.594e06) (s+2.974e04) (s+50) (s+20.69) (s+15.08) (s+12.24) (s+0.03593) (s^2^ + 4.03s + 6.88) (s^2^ + 0.003999s + 115.6) |
|  | 6 | -4.6967e-06 (s+9.594e06) (s-7.084e05) (s+2.974e04) (s+909.7) (s+50) (s+20.69) (s+15.08) (s+0.04692) (s^2^ + 9.787s + 95.59) /  (s+9.594e06) (s+2.974e04) (s+50) (s+20.69) (s+15.08) (s+13.11) (s+2.997) (s+0.1655) (s+0.03738) (s^2^ + 1.265e-07s + 130) |
|  | 7 | -6.7038e-06 (s+9.594e06) (s-5.678e05) (s+2.974e04) (s-1000) (s+50) (s+20.69) (s+15.08) (s-0.77) (s^2^ + 9.781s + 95.64) /  (s+9.594e06) (s+2.974e04) (s+50) (s+20.69) (s+0.02738)  (s^2^ + 31.21s + 243.8) (s^2^ + 0.1505s + 36.5) (s^2^ + 0.006018s + 46.08) |
|  | 8 | -5.2665e-06 (s+9.594e06) (s-7.883e05) (s+2.974e04) (s-1217) (s+50) (s+20.69) (s+15.08) (s-4.209) (s^2^ + 9.789s + 95.57) /  (s+9.594e06) (s+2.974e04) (s+50) (s+20.69) (s+15.96) (s+15.05) (s+0.104)  (s^2^ + 4.776e-07s + 13.07) (s^2^ + 0.2827s + 161.4) |
|  | 9 | -4.1814e-06 (s+9.594e06) (s-8.233e05) (s+2.974e04) (s-4117) (s+50) (s+20.69) (s+15.08) (s-0.4122) (s^2^ + 9.79s + 95.56) /  (s+9.594e06) (s+2.974e04) (s+50) (s+20.69) (s+16.11) (s+15.09) (s+0.0637) (s^2^ + 0.1327s + 11.1) (s^2^ + 0.0002993s + 157) |
|  | 10 | -4.7184e-06 (s+9.594e06) (s-7.556e05) (s+2.974e04) (s-5823) (s+50) (s+20.69) (s+15.08) (s-2.034) (s^2^ + 9.788s + 95.58) /  (s+9.594e06) (s+2.974e04) (s+50) (s+20.69) (s+15.87) (s+15.01) (s+0.1194) (s^2^ + 0.0389s + 29.51) (s^2^ + 0.3488s + 154.2) |
|  | 11 | -5.7775e-06 (s+9.594e06) (s-7.224e05) (s+2.974e04) (s+271.3) (s+50)  (s+20.69) (s+15.08) (s+0.03232) (s^2^ + 9.787s + 95.58) /  (s+9.594e06) (s+2.974e04) (s+50) (s+20.69) (s+15.08) (s+0.1171) (s+0.02904) (s^2^ + 8.167e-09s + 11.07) (s^2^ + 16.17s + 158.9) |
| **Pathway** | **Sub#** | **System identification using experimental data** |
| 3: Perivascular Potassium → vessel circumference | 1 | -0.0001314 (s+9.594e06) (s-6.287e05) (s+2.974e04) (s+50) (s+24.3) (s+20.69) (s^2^ + 9.784s + 95.62) /  (s+9.594e06) (s+2.974e04) (s+50) (s+20.69) (s+10.07) (s+3.396) (s+0.1023) (s^2^ + 0.7806s + 104.5) |
|  | 2 | 7.1761e-06 (s+9.594e06) (s+7.948e06) (s+2.974e04) (s+50) (s+20.69) (s+0.2589) (s^2^ + 9.798s + 95.27) /  (s+9.594e06) (s+2.974e04) (s+50) (s+20.69) (s+0.01201)  (s^2^ + 1.818e-07s + 1.392) (s^2^ + 14.34s + 184.3) |
|  | 3* | -6.4407e-06 (s-1.418e07) (s+9.594e06) (s+2.974e04) (s+50) (s+20.69)  (s-0.0002072) (s^2^ + 9.806s + 95.39) /  (s+9.594e06) (s+2.974e04) (s+50) (s+20.69) (s+1.85e-07)  (s^2^ + 0.7226s + 0.1593) (s^2^ + 13.63s + 134.2) |
|  | 4* | -4.0486e-06 (s-2.217e07) (s+9.594e06) (s+2.974e04) (s+50) (s+20.69)  (s+0.2873) (s^2^ + 9.807s + 95.39) /  (s+9.594e06) (s+2.974e04) (s+50) (s+20.69) (s+0.1526)  (s^2^ + 0.9892s + 0.334) (s^2^ + 13.21s + 128.8) |
|  | 5 | -0.00036085 (s+9.594e06) (s-6.958e05) (s+2.974e04) (s+50) (s+20.71)  (s+6.755) (s^2^ + 10.47s + 95.46) /  (s+9.594e06) (s+2.974e04) (s+50) (s+20.7) (s+0.004357)  (s^2^ + 1.647s + 5.839) (s^2^ + 12.69s + 1.236e04) |
|  | 6 | -0.00011381 (s+9.594e06) (s-6.888e05) (s+2.974e04) (s+50) (s+20.69) (s+0.01007) (s^2^ + 9.786s + 95.59) /  (s+9.594e06) (s+2.974e04) (s+50) (s+20.69) (s+10.94) (s+0.07898) (s+0.0003096) (s^2^ + 3.334s + 106.9) |
|  | 7 | 3.979e-05 (s+9.594e06) (s-5.539e06) (s+2.974e04) (s+50) (s+20.69)  (s-26.62) (s^2^ + 9.78s + 95.01) /  (s+9.594e06) (s+2.974e04) (s+50) (s+20.7) (s+0.0299)  (s^2^ + 4.234s + 61.46) (s^2^ + 10.08s + 866.8) |
|  | 8 | -0.00012905 (s+9.594e06) (s-7.575e05) (s+2.974e04) (s+50) (s+20.69) (s+15.37) (s^2 + 9.788s + 95.58) /  (s+9.594e06) (s+2.974e04) (s+50) (s+20.69) (s+0.07885)  (s^2^ + 11.39s + 38.1)(s^2^ + 2.882s + 72.64) |
|  | 9 | -0.00010241 (s+9.594e06) (s-7.936e05) (s+2.974e04) (s+50) (s+20.69)  (s+0.02554) (s^2^ + 9.789s + 95.57) /  (s+9.594e06) (s+2.974e04) (s+50) (s+20.69) (s+7.556) (s+0.08572) (s+0.008279) (s^2^ + 6.699s + 92.43) |
|  | 10* | 4.2886e-05 (s+9.594e06) (s+5.559e05) (s+2.974e04) (s+50) (s+20.69) (s+0.3596) (s^2^ + 9.773s + 95.01) /  (s+9.594e06) (s+2.974e04) (s+50) (s+20.69) (s+0.006075)  (s^2^ + 0.3037s + 1.635) (s^2^ + 14.04s + 198.6) |
|  | 11 | 0.00065547 (s+9.594e06) (s-2.297e05) (s+2.974e04) (s+50) (s+20.69)  (s-0.2695) (s^2^ + 9.713s + 95.37) /  (s+9.594e06) (s+2.974e04) (s+50) (s+20.69) (s+9.175) (s+0.3927) (s+0.01357) (s^2^ + 4.764s + 192.3) |
| **Pathway** | **Sub#** | **System identification using experimental data** |
| 4: Voltage gated ion channel on smooth muscle cell → vessel circumference | 1 | -1384.1 (s+9.637e06) (s+47.75) (s+19.28) (s-1.495) (s^2^ - 1.579s + 5.417)/  (s+9.594e06) (s+50) (s+20.69) (s+3.306) (s+0.2176)  (s^2^ + 9.804s + 95.24) |
|  | 2 | -442.07 (s+9.782e06) (s+42.82) (s+16.87) (s-41.28) (s^2^ - 1.584s + 1.571)/  (s+9.594e06) (s+50) (s+20.69) (s+3.307) (s+0.214)  (s^2^ + 9.804s + 95.24) |
|  | 3* | -237.98 (s+9.853e06) (s-59.05) (s+43.7) (s+17.73) (s^2^ - 1.018s + 1.018)/  (s+9.594e06) (s+50) (s+20.69) (s+3.305) (s+0.221)  (s^2^ + 9.804s + 95.24) |
|  | 4* | -63371 (s+9.596e06) (s+49.84) (s+20.57) (s+5.68) (s-0.2409) (s+0.1248)/  (s+9.594e06) (s+50) (s+20.69) (s+3.323) (s+0.1037)  (s^2^ + 9.803s + 95.23) |
|  | 5 | -788.75 (s+9.678e06) (s+45.97) (s+18.37) (s-11.58) (s^2^ - 1.587s + 1.712)/  (s+9.594e06) (s+50) (s+20.69) (s+3.306) (s+0.2184)  (s^2^ + 9.804s + 95.24) |
|  | 6 | -164.87 (s+1.005e07) (s-117) (s+41.65) (s+16.78) (s^2^ - 1.243s + 1.207)/  (s+9.594e06) (s+50) (s+20.69) (s+3.306) (s+0.217) (s^2^ + 9.804s + 95.24) |
|  | 7 | -583.73 (s+9.718e06) (s+44.6) (s+17.73) (s-22.51) (s^2^ - 1.488s + 1.519)/  (s+9.594e06) (s+50) (s+20.69) (s+3.306) (s+0.2169)  (s^2^ + 9.804s + 95.24) |
|  | 8 | -71.969 (s+1.066e07) (s-282.5) (s+40.61) (s+16.47) (s^2^ - 1.234s + 1.185)/  (s+9.594e06) (s+50) (s+20.69) (s+3.306) (s+0.2166)  (s^2^ + 9.804s + 95.24) |
|  | 9 | -88213 (s+9.595e06) (s+49.86) (s+20.58) (s+5.842) (s+0.191) (s-0.2035)/  (s+9.594e06) (s+50) (s+20.69) (s+3.301) (s+0.2129)  (s^2^ + 9.803s + 95.23) |
|  | 10* | -338.73 (s+9.796e06) (s-43.91) (s+43.7) (s+17.54) (s^2^ - 1.222s + 1.212) /  (s+9.594e06) (s+50) (s+20.69) (s+3.306) (s+0.2188)  (s^2^ + 9.804s + 95.24) |
|  | 11 | -492.03 (s+9.747e06) (s+43.88) (s+17.42) (s-30.58) (s^2^ - 1.463s + 1.471)/  (s+9.594e06) (s+50) (s+20.69) (s+3.306) (s+0.2164)  (s^2^ + 9.804s + 95.24) |

*Then, the minimal realization transfer functions obtained for the proposed pathways fitted to average tHb changes are given in Table 3.*

Table 3. Minimal realization transfer functions were obtained through Model Reducer (MATLAB, MathWorks, Inc., USA) for four model pathways from the detailed physiological model for the neurovascular unit compartment model.

| **Pathway transfer functions (TF)** | **Transfer functions from the compartmental neurovascular coupling model that were used for the initial parameterization of the four pathways** | **Parameterization** |
| --- | --- | --- |
| **TF1.**  **Synaptic Potassium → vessel circumference** | $TF1(s)= \frac{1}{(s+0.4)}TF2(s)$ | 11 poles, 3 zeros |
| **TF2.**  **Astrocytic membrane potential → vessel circumference** | $TF2\left( s \right)=\frac{\left( s+46.5 \right)}{\left( s+1.966 \right)\left( s+15.08 \right)} TF3(s)$ | 10 poles, 3 zeros |
| **TF3.**  **Perivascular Potassium → vessel circumference** | $TF3(s)=\frac{\left( s+2.371e07 \right)}{\left( s+2.974e04 \right)\left( s+1 \right)} TF4(s)$ | 8 poles, 2 zeros |
| **TF4.**  **Voltage gated ion channel on SMC → vessel circumference** | $TF4\left( s \right)=\frac{(s+2.962)}{(s+9.594e06)(s+20.69)(s+3.3)(s+0.2446)(s2 + 9.804s + 95.24)}$ | 6 poles, 1 zero |

*Here, the lowest order TF4 had 6 poles and 1 zero and the highest order TF1 has 11 poles and 3 zeros. We have now improved the Figure 7 showing our methods,*

Figure 7. Grey-box model estimation from MODEL and DATA using System Identification Toolbox (Mathworks Inc., USA) for hypothesis testing based on mean squared error, Akaike information criterion, and Chi-square Goodness-of-fit

*“Figure 7 shows our model estimation work flow diagram using the System Identification Toolbox (Mathworks, Inc., USA). The grey-box linear model with identifiable free parameters (‘idgrey’ function) was updated using the "Refine Existing Model" approach (‘greyest’ function for ‘idgrey’ model) in the System Identification Toolbox (Mathworks, Inc., USA) that uses prediction-error minimization (PEM) algorithm (‘pem’ function) to update the parameters of an initial model (from Model Linearizer tool – Section E) to improve the fit to the estimation fNIRS data of each subject – so subject-specific grey-box linear modelling.* *PEM technique considered the accuracy of the predictions computed for the observations and most tightly connected to systems theory as it explicitly exploits the dynamical structure of the studied system[23]. So, the subject-specific model evaluation in PEM was based on the properties of the prediction-error cost function for each pathway (i.e., hypothesis) for each subject constrained by the physiology of the respective tDCS perturbation pathways (see Text S1). We computed subject-specific MSE and AIC for each pathway (i.e., hypothesis) for grey-box linear model fitting.”*

**Major issue #3: Clarification of Figures 9B and 10, where a first order transfer function is mentioned both in each pathway without specifying if it’s the same one or four different ones in Fig. 9B and *one* vasoactive signal applied to all pathways in Fig. 10. In the objective to help the reader to catch directly their idea, an illustrative figure would be a combination of these two figures which would reduce discrepancies of series and parallel arrows, by adding the four intermediate variables names. Adding the variables names on the arrows and specify name or equations of function in the squares which would get rid of, among other, the discrepancy between the parallel aspect of Fig. 9B, the parallel one of Fig. 10 and the serial of the physiological model in Fig. 10.**

***Authors.*** *Apologies for the confusion. We thank the reviewer for suggesting the clarification. We have removed Figure 9 (B) and have modified figure 10 (now Figure 6) along with the description in the updated manuscript.*

*Figure 6. Schematic representation of the four pathways for tDCS current density effects (perturbation) on vessel volume function related to vessel circumference changes for hypothesis testing using experimental total hemoglobin (tHb) concentration changes (proportional to blood volume). The four simulated pathways are, Pathway 1: tDCS current density (input pulse) via first-order transfer function modulate vessel volume response (output) by perturbing synaptic potassium (K+)released from active neurons (∆*$\boldsymbol{J}_{\boldsymbol{K}_{\boldsymbol{s}}}$*), Pathway 2: tDCS current density (input pulse) via first-order transfer function modulate vessel volume response (output) by perturbing the astrocytic transmembrane current (∆*$\boldsymbol{I}_{\boldsymbol{T}}$*), Pathway 3: tDCS current density (input pulse) via first-order transfer function modulate vessel volume response (output) by perturbing perivascular potassium (K+) concentration (*${\boldsymbol{\Delta}\left[ \boldsymbol{K}^{\boldsymbol{+}} \right]}_{\boldsymbol{T}}$*), and Pathway 4: tDCS current density (input pulse) via first-order transfer function modulate vessel volume response (output) by perturbing voltage-gated ion channel current (∆*$\boldsymbol{I}_{\boldsymbol{KV}}$*) on the smooth muscle cell (SMC).*

**The accompanied text should contain these equations i.e. Sub-Section B « pathways equations or models » of the Suppl. Mat. is important to better understand the approach because they highlight the link between “stimulation” variables and studied pathways variables. Thus, this section should be incorporated in the corresponding method section.**

***Authors.*** *Thank you for your suggestions. The important modeling details have been added to the updated manuscript, as listed below (in* ***Methods section D:*** *Physiologically detailed Neurovascular Compartmental Dynamics).*

*“So, the tDCS current density at the scalp (*$I_{tdcs}$*) was proportional to the current density (*$J_{tDCS})$*in the neurovascular brain tissue based on a lead field matrix (the forward solution; see Text S2) leading to the vasoactive signal (state perturbation to NVU compartments) via first order transfer function,*

$v_{i}= \frac{K_{i}}{s/\tau+1}I_{tdcs}$	*(i)*

*where K_i_ is arbitrary gain from lead field matrix (*$J_{tDCS}={K_{i}\cdot I}_{tdcs}$*), and*$\tau$*is the time constant. Here, the state variables of various NVU compartments modeled in the study are listed in Table 2, while the detailed equations are provided in the supplementary materials (see Text S1).*

***(a) Pathway 1:*** *tDCS perturbation of synaptic potassium leading to vessel circumference changes*

*Studies have shown that K+ can act as a potent vasodilator signal that couples local neuronal activity to vasodilation in the brain, and have a major role in cerebrovascular mechanisms^83-86^. Studies have shown that the potassium pathway is responsible for the fast onset of vasodilation compared to the other mediators^59,60,87^. Synaptic activity was assumed to be modulated by tDCS current density^82^ that affected K+ release from active neurons into the synaptic space. Here,* $J_{K_{s}}$ *is the K+ released from active neurons that is considered to be perturbed by tDCS from its baseline condition such that* $\Delta J_{K_{s}}=v_{1}= \frac{K_{1}}{s/\tau+1}I_{tdcs}$*using equation (i). The potassium concentration in the synaptic space,* $\left[ K^{+} \right]_{s}$ *is then given as (see equation 1 in the Text S1):*

$\frac{d{[K^{+}]}_{s}}{dt}=$ $J_{K_{s}}\boldsymbol{+}\frac{K_{1}}{s/\tau+1}I_{tdcs}- J_{\Sigma Kmax}k_{Na}\frac{{[K^{+}]}_{s}}{{[K^{+}]}_{s}+ {KKO}_{a}}$	*(ii)*

*where* $J_{K_{s}}$*is the baseline flux of K+ in the synaptic space,* $J_{\Sigma Kmax}$ *is the maximum flux,* $k_{Na}$ *is a constant parameter that depends on extracellular sodium concentration,* ${KKO}_{a}$*is the threshold value for K+ concentration in the synaptic space,* ${[K^{+}]}_{s}$*.*

***(b) Pathway 2:*** *tDCS perturbation of the astrocytic transmembrane current leading to vessel circumference changes*

*Astrocytes are susceptible to small variations in their membrane potential^71^ and their long processes are sensitive to polarization by tDCS^72-74^. Here, astrocytic transmembrane current (*$I_{T}$*) was perturbed by tDCS,* $\Delta I_{T}=v_{2}= \frac{K_{2}}{s/\tau+1}I_{tdcs}$*from equation (i) that was added to other transmembrane currents including* $I_{BK}$ *is current through Big Potassium (BK) channel,* $I_{leak}$*is leak current,* $I_{TRPV}$*is electrical current through the TRPV channel and* $I_{\Sigma K}$ *is the electrical current carried by the K^+^ influx at the perisynaptic process (equation 21 in the Text S1):*

$\frac{dV_{k}}{dt}= \frac{1}{C_{astr}}\left( {-I}_{BK}-I_{leak}- I_{TRPV}- I_{\Sigma K}+\frac{K_{2}}{s/\tau+1}I_{tdcs} \right)$	*(iii)*

***(c) Pathway 3:*** *tDCS perturbation of perivascular potassium concentration leading to vessel circumference changes*

*Glial cells maintain extracellular K+ concentration by the imbalance in their membrane polarity and can affect K+ spatial buffering affecting tDCS modulation^36,77^. Astrocytic release of K+, via two potassium channels (BK and KIR), into the perivascular space can be perturbed by tDCS. Astrocytic role in neurovascular coupling may be related to the strength of stimulation where high strength can lead to vasoconstriction[19] (mediated via K+ and EET signalling[21]). Vasoconstriction can follow vasodilation when the astrocytic calcium concentration (or, perivascular K+ concentration) increase above a certain threshold. We assumed that low-intensity tDCS perturbation would not cross that threshold where* ${\Delta\left[ K^{+} \right]}_{T}=v_{3}= \frac{K_{3}}{s/\tau+1}I_{tdcs}$*. So, the perivascular potassium concentration,*$\left[ K^{+} \right]_{P}$*, is given as (equation 26, Text S1):*

$\frac{d\left[ K^{+} \right]_{P}}{dt}= \frac{J_{BK}}{{VR}_{pa}}+ \frac{J_{KIR}}{{VR}_{ps}}- R_{decay}\left( \left[ K^{+} \right]_{P}- \left[ K^{+} \right]_{P,min} \right)+\frac{K_{3}}{s/\tau+1}I_{tdcs}$	*(iv)*

*Here,* ${[K^{+}]}_{P,min}$ *is the resting state equilibrium K^+^ concentration in the perivascular space. The K+ flow from the astrocyte and SMC are* $J_{BK}$ *and* $J_{KIR}$*corresponding to BK and inward rectifying potassium (KIR) respectively. And,* ${VR}_{pa}$ *and* ${VR}_{ps}$ *are the volume ratios of perivascular space to astrocyte and SMC, respectively.* $R_{decay}$ *is the rate at which perivascular K^+^ concentration decays to its baseline state.*

***(d) Pathway 4:*** *tDCS perturbation of voltage-gated ion channel current on the smooth muscle cell leading to vessel circumference changes*

*Smooth muscle cell (SMC) voltage-gated potassium (KV) channels and inwardly rectifying K+ channels are important in penetrating arterioles that control arterial diameter by exerting a major hyperpolarizing influence[22]. Therefore, tDCS electric field can perturb voltage gated potassium current (*$\Delta I_{KV}=v_{4}= \frac{K_{4}}{s/\tau+1}I_{tdcs}$*) that was added to other currents including I_L_, I_K_, I_Ca_ and I_KIR_ that represent leak, K+, Ca2+, and KIR channel currents respectively in the SMC compartment. Then, the SMC membrane potential, V_SMC_, is given by (equation 41, Text S1):*

$\frac{dV_{SMC}}{dt}= \frac{1}{C_{SMC}}\left( -I_{L}- I_{K}-I_{Ca}-I_{KIR}- I_{KV}+\frac{K_{4}}{s/\tau+1}I_{tdcs} \right)$	*(v)*

*Here, the four tDCS perturbation pathways are nested, i.e., the pathway 1 is represented by seventeen ordinary differential equations starting from synaptic K+ (see Text S1) that nested other tDCS perturbation pathways 2-4 – see Figure 6.”*

*The detailed physiological model equations are given in the Supplementary Materials.*

**Major issue #4: section (D) participant-specific grey-box linear model dynamics**

**The use of the system identification toolbox of Matlab must be better explained in this section by specifying the** **variables obtained in the grey-box model; And in the following section the variables ‘tfest’ obtained by the reduction named ‘balred’.**

***Authors.*** *Thank you for your suggestions. We have modified the figure 7 and added the description in* ***Methods Sections F and G***

*Figure 7. Grey-box model estimation from MODEL and DATA using System Identification Toolbox (Mathworks Inc., USA) for hypothesis testing based on mean squared error, Akaike information criterion, and Chi-square Goodness-of-fit*

*“Figure 7 shows our model estimation work flow diagram using the System Identification Toolbox (Mathworks, Inc., USA). The grey-box linear model with identifiable free parameters (‘idgrey’ function) was updated using the "Refine Existing Model" approach (‘greyest’ function for ‘idgrey’ model) in the System Identification Toolbox (Mathworks, Inc., USA) that uses prediction-error minimization (PEM) algorithm (‘pem’ function) to update the parameters of an initial model (from Model Linearizer tool – Section E) to improve the fit to the estimation fNIRS data of each subject – so subject-specific grey-box linear modelling. PEM technique considered the accuracy of the predictions computed for the observations and most tightly connected to systems theory as it explicitly exploits the dynamical structure of the studied system[23]. So, the subject-specific model evaluation in PEM was based on the properties of the prediction-error cost function for each pathway (i.e., hypothesis) for each subject constrained by the physiology of the respective tDCS perturbation pathways (see Text S1). We computed subject-specific MSE and AIC for each pathway (i.e., hypothesis) for grey-box linear model fitting.”*

***“(G) Investigation of a Reduced Dimension Grey-box Linear Model for averaged fNIRS data:***

*Linearized grey-box model's complexity (see Table S3) was simplified with reduced-order approximations using the Model Reducer app in the Control System Toolbox (Mathworks, Inc., USA). Simpler models can preserve model characteristics while discarding states that contribute relatively little to system dynamics (Balanced Truncation Model Reduction, ‘balred’ function). Simpler transfer function models can provide insights into the linear time-invariant model dynamics that were derived from the minimal realization transfer function for the four tDCS perturbation pathways. A generic reduced dimension grey-box linear model was derived (‘tfest’ function) using the nested linearized grey-box models for the initial parameterization then fitted to averaged fNIRS-tHb data – see Figure 7. We used Chi-Square Goodness-of-Fit for comparing the four hypotheses for the nested pathways (see Figure 6) where Chi-Square difference test for nested pathways^90^ determined the tDCS perturbation pathway model of the least order.”*

*The subject-specific transfer functions in ‘s’ domain obtained from system identification toolbox is given in Table S3: System identification using experimental data (total hemoglobin changes from stimulated hemisphere) from 11 subjects (Sub). * subjects with >-0.5 Correlation Coefficient between Oxy-Hb & Dxy-Hb.*

**Minor issues: Overall review**

**The authors could improve the overall aspect and coherence of the manuscript by**

** Reviewing typos: at the end of section titles, spaces after commas and full stops ;**

***Authors.*** *Apologies, we have tried to fix all the typos in the revised draft.*

** Choosing meaningful variable names as, for instance, *[Ca^2+^]_A_* instead of *[Ca^2+^]* and keep it through the document. Another example is *V_SMC_* instead of *V_m_* which is generally taken for the membrane potential of neurons and not at the level of vessels;**

***Authors.*** *Thank you for your suggestions, we have now changed relevant variable names in the main manuscript and supplementary material.*

** Also referring more often to the equations number helps the reader to follow the developed idea of the text and facilitate the possible jumps between linked sections, *including* in the figures captions;**

***Authors.*** *Thank you for your suggestions. We have referred to the equations as required in the various sections.*

** Adding pages number and lines facilitates reviewing and response to review.**

**Minor issue #1: Figures improvement**

** Please increase the police size of labels if possible.**

***Authors.*** *We have updated and arranged the figures vertically for a clear view in the updated manuscript.*

** Colors of Fig. 8 may be more segregating.**

***Authors.*** *Thank you for your suggestion. We have now updated Figure 8 that now has more segregating colors.*

** Fig. 4 would benefit spatially and therefore gain sharpness to be organized (A and B) vertically instead of horizontally**

***Authors.*** *Thank you for your suggestions. We have placed the two figures vertically in the updated manuscript.*

**Minor issue #2: Text improvement – example of sub-section C of the Methods section**

**A certain number of sentences could be rethink to be more pedagogical in the direction of *organization* of ideas inside a sub-section which also will avoid repetition. An example is the sentence “the TDCS current density in the brain’s neurovascular tissue was assumed to be proportional…” and the same one “transcranial electrical stimulation induced current density … arbitrary gain” (around Eq. 1).**

***Authors.*** *Thank you for your suggestions. We have reorganized the content in the manuscript and presented the details without repetition.*

** Improvement of the vocabulary *precision***

**For remarkable example, the different occurrences of the term model (physiologically detailed model or physiological model, pathway models or grey-box model, …) to discriminate the steps of the study’s framework should be chosen once and kept throughout the manuscript to strengthen the understanding of this crucial point of hypothesis testing). One occurrence in “Figure 10 shows …”**

***Authors.*** *Apologies, we have now fixed all the different occurrences of the term “* *physiologically detailed model” in the revised manuscript.*

** Explanation of the Biological criteria in “We identified four nested ways …”**

***Authors.*** *Thank you for your suggestions. We have modified figure 10 (now Figure 6) to depict the nested pathways. And, we have added the description in* ***Methods Section D*** *has been added as:*

*“* Our goal was mechanistic grey-box modeling for hypothesis testing, where the hypotheses are formulated as a set of mathematical equations for data fitting to identify core predictions from biological criteria^81^. We identified four nested NVU compartmental pathways (see Figure 6) where tDCS perturbed a state variable at each of the four NVU compartments (see Table 2 for details on compartmental state variables) for hypothesis testing of the initial (0-150sec) tDCS effects on the NVU leading to blood vessel (circumference) volume changes. Figure 6 shows the nested model for each of the four pathways starting from tDCS scalp current density that perturbed synaptic potassium (K+) released from active neurons (∆$J_{K_{s}}$) for Pathway 1, astrocytic transmembrane current (∆$I_{T}$) for Pathway 2, perivascular potassium (K+) concentration (${\Delta\left[ K^{+} \right]}_{T}$) for Pathway 3, and voltage-gated ion channel current (∆$I_{KV}$) on the smooth muscle cell (SMC) for Pathway 4.*”.*

***Structure* of sentences: be sure that sentences contain a verb (example “In pathway 1, the vasoactive …”)**

***Authors.*** *Apologies*, *we have tried to fix all these typos in the revised draft.*

**Minor issue #3: Table 3**

**The authors did a real effort in the bibliography of existing models summarized in this table. Still it can be easily improved by the following specifications:**

** Replacing potassium by K+ everywhere ease to catch similarities across studies.**

** NVU system 1: output = cerebral blood volume and cerebral blood flow**

** NVU system 3: output = arteriolar radius**

** NVU system 4: input = synaptic current, output = vessel radius via EET signaling**

** NVU system 10: input = EPSP and FR from …**

** NVU system 14: and astrocytes**

**Model number 9 in this table appears not interesting here to me as it doesn’t have output at the level of vessels. I however suggest these references as models incorporating astrocytes and a neurovascular coupling input-output link:**

** Riera, J et al. “Fusing EEG and fMRI based on a bottom-up model: inferring activation and effective connectivity in neural masses.” *Philosophical transactions of the Royal Society of London. Series B, Biological sciences* vol. 360,1457 (2005): 1025-41.**

** Riera JJ, Wan X, Jimenez JC, Kawashima R. Nonlinear local electrovascular coupling. I: A theoretical model. Hum Brain Mapp. 2006 Nov;27(11):896-914.**

**Without astrocytes:**

** Banaji M, Mallet A, Elwell CE, Nicholls P, Cooper CE. A model of brain circulation and metabolism: NIRS signal changes during physiological challenges. PLoS Comput Biol. 2008 Nov;4(11):e1000212.**

***Authors.*** *Thank you for your suggestions. We have included the suggested models and removed model number 9 in the updated draft. Also, the specific points in the table 1 have been addressed, as listed below.*

“Table 1. Summary of studies detailing mathematical models of the neurovascular unit (NVU) components and their input (I) and output (O)

| **S. No.** | **Study** | **NVU system** | **Description** |
| --- | --- | --- | --- |
| 1 | Riera et al., 2005^61^ | Synaptic space, neuron-astrocyte interface, capillary, post capillary; I: stimulus (afferent pathways); O: vessel volume | The study used a bottom-up physiological model for fusing EEG and fMRI data depicting the electro-vascular coupling. Synaptic activity and hemodynamics were governed by mesoscopic dynamic equations system. |
| 2 | Riera et al., 2006^62^ | Canonical neural mass component, electrovascular coupling component, vascular state dynamics component; I: excitatory synaptic inputs; O: Cerebral blood volume and cerebral blood flow | The study presented the cerebral architecture, electro-vascular coupling and energy considerations with respect to EEG and fMRI data fusion. The study extended balloon approach for modeling the vascular changes. |
| 3 | Bennett et al., 2008^56^ | Synaptic space, astrocyte, SMC; I: Glutamate released from glutamatergic synapse; O: Cerebral blood volume and cerebral blood flow | The study showed that the coupling between glutamatergic synapses to arteriolar SMC is mediated by astrocytic epoxyeicosatrienoic acids (EETs). Results depicted a linear rise in blood flow with an increase in numbers of activated astrocytes; however, the response was non-linear with respect to the release of glutamate. |
| 4 | Banaji et al., 2008^63^ | Cerebral circulation component, mitochondrial metabolism component; I: Blood pressure changes, changes in arterial oxygen and carbon-dioxide levels, functional activation; O: cerebral metabolic rate of oxygen (CMRO2), changes in oxidation level | The study modeled the brain circulation and metabolism to analyze the experimental fNIRS data in response to various stimuli. |
| 5 | Farr and David, 2011^57^ | Synaptic space, astrocyte, perivascular space, SMC, and endothelial cells; I: Glutamate and K^+^ in synaptic space; O: arteriolar diameter | The study showed that the coupling between neuronal activation (due to K^+^ and glutamate) to arteriolar dilation was mediated by astrocytic K^+^, EET, and Ca^2+^. |
| 6 | Witthoft and Karniadakis, 2012^42^ | Synaptic space, astrocyte, perivascular space, SMC; I: Glutamate and K^+^ in synaptic space; O: arteriolar radius | The study showed the bidirectional communication between cerebral astrocytes and the microvessels. Major signaling pathways considered were: neural synaptic K^+^ and glutamate to astrocytes, K^+^ signaling between astrocytes and microvasculature, and microvasculature to astrocytes via astrocyte perivascular endfoot. |
| 7 | Chander and Chakravarthy, 2012^64^ | Neuron, astrocyte, vessel, and interstitium; I: Synaptic current and Adenosine Triphosphate (ATP); O: vessel radius via EET signaling | The study presented a model for the neuron-astrocyte-vessel loop based on neuronal and metabolic activity. |
| 8 | Witthoft et al., 2013^65^ | Synaptic space, astrocyte, perivascular space, SMC; I: Glutamate and K^+^ in synaptic space; O: arteriole radius | Extended version of the model from Witthoft and Karniadakis, 2012^42^with K^+^ buffering across all components of NVU. |
| 9 | Chang et al., 2013^66^ | Soma, dendrite, extracellular space, vascular tree compartment, glial compartment; I: extracellular K^+^; O: vessel radius | The study demonstrated the coupling between the vascular diameters and neuronal activity mediated by K^+^ concentrations in extracellular space in the vicinity to dendritic processes that were assumed to be buffered through astrocytes. |
| 10 | Dormanns et al., 2015^67^ | Neuron, synaptic cleft, astrocyte, perivascular space, SMC, endothelial cell, and arteriolar lumen; I: synaptic K^+^; O: arteriole radius | The study used lumped model of the NVU for depicting the connection between a neuron and the perfusing arteriole through the astrocytic perivascular K^+^ signaling and the SMC’s Ca^2+^ dynamics. The study showed the significance of luminal agonists in flowing blood influencing the endothelial and SMC dynamics. |
| 11 | Dormannset al., 2016^58^ | Neuron, synaptic cleft, astrocyte, perivascular space, SMC, endothelial cell, and arteriolar lumen; I: synaptic K^+^; O: arteriole radius | An extended version of Dormannset al., 2015 model^67^with NO signaling pathway. The model considered the production of NO in the neuron and the endothelial cell compartments and its diffusion in the other compartments. |
| 12 | Blanchard et al., 2016^68^ | Pyramidal cells, interneurons, extracellular space, astrocytes, vessels; I: Excitatory postsynaptic potential (EPSP) and Firing rates (FR) from pyramidal cells and interneurons; O: local field potential and regional cerebral blood flow | The study demonstrated the connection between the neuronal activity and regional CBF via neuro-glio-vascular link at the population scale (voxel). The model evaluated the role of astrocytes in glutamate and GABA recycling, which then influences adjoining vessels |
| 13 | Mathias et al., 2017^69^ | Soma, dendrite, extracellular space, synaptic space, astrocyte, perivascular space, SMC, endothelial cell and lumen; I:neural activation through ion channels; O: fMRI BOLD signal | The study demonstrated the signaling method of neurovascular coupling through a model of pyramidal neurons and its analogous fMRI BOLD response. The study extended the NVU to include a complex neuron system with Na/K ATPase pump mechanism, which provides CBF and CMRO2. |
| 14 | Kenny et al., 2018^59^ | Neuron, synaptic cleft, astrocyte, perivascular space, endothelial cell, SMC, and lumen; I: Glutamate and K^+^ in synaptic space; O: arteriolar radius | The model used lumped parameter systems to depict the connection between a neuron and perfusing arteriole through the astrocytic perivascular K^+^ and the SMC’s Ca^2+^ dynamics mediated by astrocytic EETs and TRPV4. Results indicated that K^+^ mediated pathway drives the quick start of vaso-dilation compared to the NO-mediated pathway. |
| 15 | Mathias et al., 2018^60^ | Neuron (soma, dendrite), extracellular space, synaptic space, astrocyte, perivascular space, SMC, endothelial cell and lumen; I:neuronal current; O: fMRI BOLD signal | The model simulated NVU mechanisms and BOLD signal by extending the previous models by Mathias et al., 2017^69^ and Kenny et al., 2018^59^. The study included a transient sodium ion channel in the neuron compartment. |
| 16 | Sten et al., 2020^70^. | Pyramidal neuron, GABAergic interneuron, astrocyte, SMC, arteriole; I: neuronal pulse mediating vaso-agents; O: arteriolar diameter | The study modeled the interplay between pyramidal neurons and GABAergic interneurons in the NVU. The study evaluated the role of cell-specific contributions in NVU due to the effect of an anesthetic agent. |

**”**

**Minor issues #4: References**

**References to literature could be added throughout the manuscript. For instance**

** First sentence of the abstract**

** First § of the introduction about the three mechanisms of CBF regulation**

** Second § of the introduction about effects of the synaptic projections, astrocytes and SMC on vessels after stimulation (done with perivascular compartment)**

** Third § of the introduction for the sentence “as evident from studies”**

***Authors.*** *Thank you for your suggestions. We have included the references at the required places in our revised draft.*

**Minor issues #5: Supplementary materials (Suppl. Mat.)**

** Eqs. 21 and 26 and 41 of pathways variables should include the stimulation variables as in sub-section B (like in Eq. 1) with an accompanied sentence specifying them, to avoid confusion created by two different equations of the same variable**

***Authors.*** *Thank you for your suggestions. We have now changed the variable names in the main manuscript (Table 2) and supplementary material (Text S1). For pathway specific equations, we have given the representations in the main text and have given the description along with the necessary equations.*

** Clarify the role of variable ω of Eq. 50 and that *q* is a power in Eq. 51, even if it’s not specified as such in the original publication, as these variables make the link between vessels variable of interest *x* and the upstream dynamics**

***Authors.*** *Thank you for your suggestion. We have mentioned the role of ω in the revised draft. The mechanisms regarding myosin actin cross bridges and their mathematical representations are given in detail are given in the cited reference (Gonzalez-Fernandez, Jose M., and Bard Ermentrout. "On the origin and dynamics of the vasomotion of small arteries." Mathematical biosciences 119.2 (1994): 127-167.)*

** Specify *w_m_* of Eq. 55**

***Authors.*** *Apology for the typo. w_m_ was mistakenly written as w_u_ which is now corrected.*

** The sentence “and x = u + y “ at the end of Eq. 55 is a complementary specification to Eq. 52, thus it should be reformulated to help understanding the link between *x* and Ψ via Eq. 59**

***Authors.*** *Apology for the confusion. The text has been rephrased.*

** No number is given to the “transcranial electrical stimulation” section. Besides, I don’t see the reference of this section in the main text neither in the corresponding § of subsection B of Suppl. Mat.**

***Authors.*** *Thank you for your suggestion. We have numbered the section “transcranial electrical stimulation” in the supplementary materials, Text S2. Also, this section has been referred to in the main text.*

** The normalization taken for data *deoxy-Hb*, *oxy-Hb* and *tHb* in Table S2 should be explained.**

***Authors.*** *Thank you for your suggestion. The figures in Table S2 depicting deoxy-Hb*, *oxy-Hb* and *tHb were normalized for comparison during tDCS stimulation period, as clarified now.*

*“**Here, pre-tDCS baseline (mean value) was subtracted and then the time course was normalized with the maximum (the maximum value gets transformed into a 1) for the changes in the oxy, deoxy and total hemoglobin concentration during the entire stimulation. The normalized time courses are presented for comparison during tDCS stimulation period as the continuous wave-fNIRS system used in the experimental setup provided relative values.”*

** Table S1 could be organized in sub-tables (by compartment for instance) to facilitate the search of a particular parameter value**

***Authors.*** *Thank you for your suggestion. We have compartment wise organized the Table S1 as shown below.*

*“Supplementary Table S1: Model Parameters*

| Compartment | Parameter | Value | Unit |
| --- | --- | --- | --- |
| Synaptic Space | $J_{\Sigma Kmax}*k_{Na}$ | 0.6 | mM/s |
|  | ${KKO}_{a}$ | 1.5 | mM |
|  | $K_{G}$ | 8.82 | - |
|  | $\delta$ | 0.001235 | - |
| Astrocytic Intracellular Space | $r_{h}$ | 4.8 | μM |
|  | $k_{deg}$ | 1.25 | s^-1^ |
|  | $K_{Glu}$ | 1 | mM |
|  | $\beta$ | 0.0244 | - |
|  | $J_{max}$ | 2880 | μM/s |
|  | $K_{I}$ | 0.03 | μM |
|  | $K_{act}$ | 0.17 | μM |
|  | $V_{max}$ | 20 | μM/s |
|  | K_p_ | 0.24 | μM |
|  | $P_{L}$ | 5.2 | μM |
|  | $\left[ {Ca}^{2+} \right]_{ER}$ | 400 | μM |
|  | $C_{astr}$ | 40 | pF |
|  | $g_{TRPV}$ | 200 | pS |
|  | $v_{TRPV}$ | 6 | mV |
|  | $k_{on}$ | 2 | μM^-1^s^-1^ |
|  | $K_{inh}$ | 0.1 | μM |
|  | $\tau_{TRPV}$ | 0.9 | s^-1^ |
|  | $\varepsilon_{1/2}$ | 0.16 | - |
|  | $\kappa$ | 0.04 | - |
|  | $\gamma_{{Ca}_{i}}$ | 0.2 | μM |
|  | $\gamma_{{Ca}_{e}}$ | 0.2 | mM |
|  | $v_{1,TRPV}$ | 120 | mV |
|  | $v_{2,TRPV}$ | 13 | mV |
|  | $V_{EET}$ | 72 | s^-1^ |
|  | $\left[ {Ca}^{2+} \right]_{min}$ | 0.1 | μM |
|  | $k_{EET}$ | 7.1 | s^-1^ |
|  | $\Psi_{BK}$ | 2.664 | s^-1^ |
|  | $v_{4,BK}$ | 14.5 | mV |
|  | $v_{5,BK}$ | 8 | mV |
|  | $v_{6,BK}$ | -15 | mV |
|  | ${EET}_{shift}$ | 2 | mV/μM |
|  | ${Ca}_{3,BK}$ | 400 | nM |
|  | ${Ca}_{4,BK}$ | 150 | nM |
|  | $g_{BK}$ | 225.6 | pS |
|  | $v_{BK}$ | -95 | mV |
|  | $g_{leak}$ | 78.54 | pS |
|  | $v_{leak}$ | -70 | mV |
| Perivascular Space | $\left[ {Ca}^{2+} \right]_{P,min}$ | 2000 | μM |
|  | ${[K^{+}]}_{P,min}$ | 3 | mM |
|  | ${Ca}_{decay}$ | 0.5 | s^-1^ |
|  | $R_{decay}$ | 1 | s^-1^ |
|  | ${VR}_{pa}$ | 3.2e-5 | - |
| Arteriole Smooth Muscle Cell (SMC) Intracellular Space | $\tau$ | 0.2 | dyne/cm |
|  | $\Delta_{p}$ | 60 | mmHg |
|  | $w_{e}$ | 0.9 | - |
|  | A | 502.65 | μm^2^ |
|  | $S$ | 40000 | μm^2^ |
|  | $w_{m}$ | 0.7 | - |
|  | $\sigma_{0}^{\#}$ | 3e+6 | dyne/cm^2^ |
|  | $x_{0}$ | 188.5 | μm |
|  | $x_{1}^{'}$ | 1.2 | - |
|  | $x_{2}^{'}$ | 0.13 | - |
|  | $x_{3}^{'}$ | 2.2443 | - |
|  | $x_{4}^{'}$ | 0.71182 | - |
|  | $x_{5}^{'}$ | 0.8 | - |
|  | $x_{6}^{'}$ | 0.01 | - |
|  | $x_{7}^{'}$ | 0.32134 | - |
|  | $x_{8}^{'}$ | 0.88977 | - |
|  | $x_{9}^{'}$ | 0.0090463 | - |
|  | $u_{1}^{'}$ | 41.76 | - |
|  | $u_{2}^{'}$ | 0.047396 | - |
|  | $u_{3}^{'}$ | 0.0584 | - |
|  | $y_{0}^{'}$ | 0.928 | - |
|  | $y_{1}^{'}$ | 0.639 | - |
|  | $y_{2}^{'}$ | 0.35 | - |
|  | $y_{3}^{'}$ | 0.78847 | - |
|  | $y_{4}^{'}$ | 0.8 | - |
|  | $C_{SMC}$ | 19.635 | pF |
|  | $g_{KIR,0}$ | 145 | pS |
|  | $v_{KIR,1}$ | 57 | mV |
|  | $v_{KIR,2}$ | 130 | mV |
|  | $\gamma$ | 1970 | mV/μM |
|  | $g_{Ca}$ | 157 | pS |
|  | $v_{Ca}$ | 80 | mV |
|  | $v_{1}$ | -21 | mV |
|  | $v_{2}$ | 25 | mV |
|  | $\alpha_{KIR}$ | 1020 | S |
|  | $a_{v_{1}}$ | 18 | mV |
|  | $a_{v_{2}}$ | 10.8 | mV |
|  | $\beta_{KIR}$ | 26.9 | S |
|  | $b_{v_{1}}$ | 18 | mV |
|  | $b_{v_{1}}$ | 0.06 | mV |
|  | $g_{K}$ | 251.33 | pS |
|  | $v_{k}$ | -80 | mV |
|  | $g_{L}$ | 62.832 | pS |
|  | $v_{l}$ | -70 | mV |
|  | $\phi_{n}$ | 2.664 | - |
|  | ${Ca}_{3}$ | 400 | nM |
|  | ${Ca}_{4}$ | 150 | nM |
|  | $v_{4}$ | 14.5 | mV |
|  | $v_{5}$ | 8 | mV |
|  | $v_{6}$ | -15 | mV |
|  | $K_{d}$ | 1000 | nM |
|  | $B_{T}$ | 10000 | nM |
|  | $\alpha$ | 4.3987e+15 | nM/C |
|  | $k_{ca}$ | 1.3568e+2 | s^-1^ |
|  | $k_{\Psi}$ | 3.3 | - |
|  | Ca_m_ | 500 | nM |
|  | $[{Ca}^{2+}]_{SMC,ref}$ | 510 | nM |
|  | $\Psi_{m}$ | 0.3 | - |
|  | Q | 3 | - |
|  | $\sigma_{y_{0}}^{\#}$ | 2.6e+6 | dyne/cm^2^ |
|  | v_ref_ | 0.24 | - |
|  | $a^{'}$ | 0.28125 | - |
|  | $b^{'}$ | 5 | - |
|  | $c^{'}$ | 0.03 | - |
|  | $d^{'}$ | 1.3 | - |

*”*

********
